# MoO_2_‐Mediated Ni─Fe Bond Contraction and Electronic Modulation in Ni_3_Fe Alloy for Efficient Water Electrolysis at High‐Current‐Densities

**DOI:** 10.1002/adma.202512658

**Published:** 2025-09-30

**Authors:** Liancen Li, Haotian Xu, Guangfu Qian, Xinyu Cao, Jiawei Li, Yihao Xu, Ruyu Zhang, Douyong Min, Jinli Chen, Panagiotis Tsiakaras

**Affiliations:** ^1^ Guangxi Key Laboratory of Clean Pulp & Papermaking and Pollution Control School of Light Industry and Food Engineering Guangxi University Nanning 530004 China; ^2^ State Key Laboratory of Materials Processing and Die & Mould Technology School of Materials Science and Engineering Huazhong University of Science and Technology Wuhan 430074 China; ^3^ Laboratory of Alternative Energy Conversion Systems Department of Mechanical Engineering School of Engineering University of Thessaly 1 Sekeri Street Pedion Areos 38834 Greece

**Keywords:** carbonized wood, heterogeneous interface, hydrogen evolution reaction, oxygen evolution reaction, superaerophobicity

## Abstract

Ni_3_Fe alloy electrocatalysts show promising activity for water electrolysis but are limited by sluggish hydrogen/oxygen evolution reaction (HER/OER) kinetics, and inefficient gas‐liquid mass transfer under high‐current‐densities. Here, a superhydrophilic/superaerophobic 3D carbonized wood‐loaded Ni_3_Fe‐MoO_2_ (Ni_3_Fe/MoO_2_/CW) heterojunction is designed to address these challenges. X‐ray absorption fine structure (XAFS) and theoretical calculations reveal that the introduction of MoO_2_ shortens the Ni─Fe bond length, induces electron transfer from Ni_3_Fe to MoO_2_, and regulates the *d*‐band center of Ni/Fe. These optimized Ni─Fe bonds and electronic structure enhance H─OH bond dissociation and H* adsorption/desorption, thereby accelerating the HER Volmer‐Heyrovsky step. Simultaneously, for the OER adsorption evolution mechanism on Ni_3_Fe (1.462 eV), the strengthened Ni─O─Mo bond on Ni_3_Fe‐MoO_2_ heterojunction reduces the energy barrier (1.092 eV) of the rate‐determining step, significantly improving catalytic efficiency. Thus, Ni_3_Fe/MoO_2_/CW displays good activity (HER: *η*_−10/−750_ = 45/342 mV; OER: *η*_300/1000_ = 251/306 mV). Notably, the large specific area of Ni_3_Fe/MoO_2_/CW from its nanosheet‐particle structure enhances the electrolyte/bubble exchange at the gas‐liquid‐solid three‐phase interface, enabling stable operation at 1000 mA cm^−2^ for 24 h in an anion exchange membrane electrolyzer. This work demonstrates a MoO_2_‐driven strategy for electronic modulation and metal bond regulation to boost HER/OER kinetics, advancing Ni_3_Fe‐based catalysts toward practical high‐current‐densities water electrolysis.

## Graphical abstract

MoO_2_ effectively modulates the electronic structure of Ni_3_Fe and optimizes the Ni─Fe bonding configuration, thereby enabling dual regulation of the Volmer‐Heyrovsky steps and the evolution pathways of reaction intermediates during the water electrolysis process, which collectively accelerates both the hydrogen evolution reaction and oxygen evolution reaction; in addition, the catalyst exhibits superhydrophilic/superaerophobic properties, facilitating electrolyte exchange and bubble release.

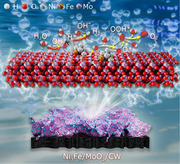

## 1 Introduction

The urgent demand for energy transition and carbon neutrality has accelerated the development of green hydrogen technologies. Among them, water electrolysis powered by renewable energy is considered a promising route due to its zero‐carbon footprint and high energy density.^[^ ^1^, ^2^ ^]^ However, large‐scale deployment remains hindered by sluggish hydrogen and oxygen evolution kinetics, which require substantial overpotentials.^[^ ^3^, ^4^ ^]^ While noble metal catalysts (e.g., Pt, Ru) show excellent activity, their scarcity and high cost limit practical use, driving interest toward transition metal‐based alternatives.^[^ ^5^ ^]^ NiFe alloys have attracted particular attention owing to their abundance, low cost, and outstanding oxygen evolution reaction (OER) activity in alkaline media, comparable to noble metals.^[^ ^6^, ^7^, ^8^ ^]^ Their OER performance is generally attributed to the synergistic effect of Fe incorporation, which modulates the Ni electronic structure and promotes active *β*‐NiOOH formation.^[^ ^9^ ^]^ Nevertheless, NiFe alloys suffer from poor hydrogen evolution reaction (HER) activity because Fe^3+^ enhances water dissociation but simultaneously renders H* adsorption on Ni sites too strong, hampering H_2_ release during the Heyrovsky step.^[^ ^10^ ^]^ This imbalance between HER and OER undermines the overall bifunctional efficiency of NiFe‐based electrocatalysts.

Heterojunction engineering provides an effective strategy to address this challenge by facilitating interfacial charge transfer and optimizing electronic configurations, thereby lowering reaction barriers for both HER and OER.^[^ ^11^, ^12^, ^13^, ^14^ ^]^ In particular, Mo‐based compounds have been shown to promote water dissociation and regulate H* adsorption/desorption in alkaline media, thus accelerating HER kinetics and tuning OER pathways.^[^ ^15^, ^16^, ^17^, ^18^ ^]^ Despite these advances, most heterojunction catalysts are either powder‐based or supported on costly metal foams, both of which limit large‐scale application due to binder‐induced detachment or fabrication complexity.^[^ ^19^, ^20^, ^21^ ^]^

Wood‐derived carbon offers a promising alternative as a self‐supporting substrate, combining low cost, good conductivity, and a hierarchical porous framework that maximizes active site exposure and mass transport.^[^ ^22^, ^23^, ^24^ ^]^ For instance, Ni nanoparticles encapsulated in nitrogen‐doped carbon on delignified wood exhibited ultralow overpotentials and long‐term stability due to enhanced electron/electrolyte transfer.^[^ ^25^ ^]^ Nonetheless, wood‐derived carbon‐based catalysts still face challenges such as insufficient intrinsic activity and poor surface wettability, underscoring the need for rational structural and electronic modulation to achieve high‐performance water splitting.

Inspired by these ideas, herein, we report the in‐situ growth of a Ni_3_Fe/MoO_2_ heterojunction on wood‐derived carbon (named as Ni_3_Fe/MoO_2_/CW) using vacuum impregnation, solvothermal treatment, and a calcination process, aiming at enhancing water electrolysis efficiency. The Ni_3_Fe/MoO_2_/CW catalyst exhibits low overpotentials in alkaline solution, with *η*_−10_ = 45 mV for the HER and *η*_300_ = 251 mV for the OER. X‐ray absorption fine structure (XAFS) analyses combined with density functional theory (DFT) calculations reveal that the incorporation of MoO_2_ into Ni_3_Fe leads to the formation of a heterojunction, which promotes electron transfer from Ni_3_Fe to MoO_2_ via strong interfacial coupling. This electronic redistribution shifts the *d*‐band center of Ni_3_Fe away from the Fermi level, while the presence of MoO_2_ also induces the contraction of the Ni─Fe bond. These changes optimize the binding energies between the Ni/Fe active sites and key intermediates such as H* and O*. The modulated Ni/Fe─H* interaction facilitates H─OH bond cleavage and improves the adsorption/desorption dynamics of H*, thereby accelerating the Volmer‐Heyrovsky mechanism in the HER process. Concurrently, the energy barrier for the rate‐determining step (RDS) of OER is effectively reduced under the adsorbate evolution mechanism, resulting in faster reaction kinetics and enhanced overall catalytic efficiency. These findings underscore the potential of the Ni_3_Fe/MoO_2_/CW heterojunction for enabling highly efficient water electrolysis.

Furthermore, the combination of Ni_3_Fe/MoO_2_ porous nanosheets with the 3D framework of wood‐derived carbon not only maximizes the exposure of active sites but also endows the catalyst with superhydrophilic and superhydrophobic characteristics, facilitating efficient mass and charge transport during electrolysis. As a result, in an anion exchange membrane electrolyzer operating under simulated alkaline industrial conditions, Ni_3_Fe/MoO_2_/CW achieves a current density of 1000 mA cm^−2^ at a low cell voltage of 2.069 V and maintains stable performance for 24 h. This study underscores the catalyst's high efficiency and durability, providing new strategies for developing NiFe‐based catalysts for large‐scale water electrolysis.

## 2 Results and Discussion

### 2.1 Synthesis and Characterization of Heterostructure Ni_3_Fe/MoO_2_/CW

**Figure 1:**
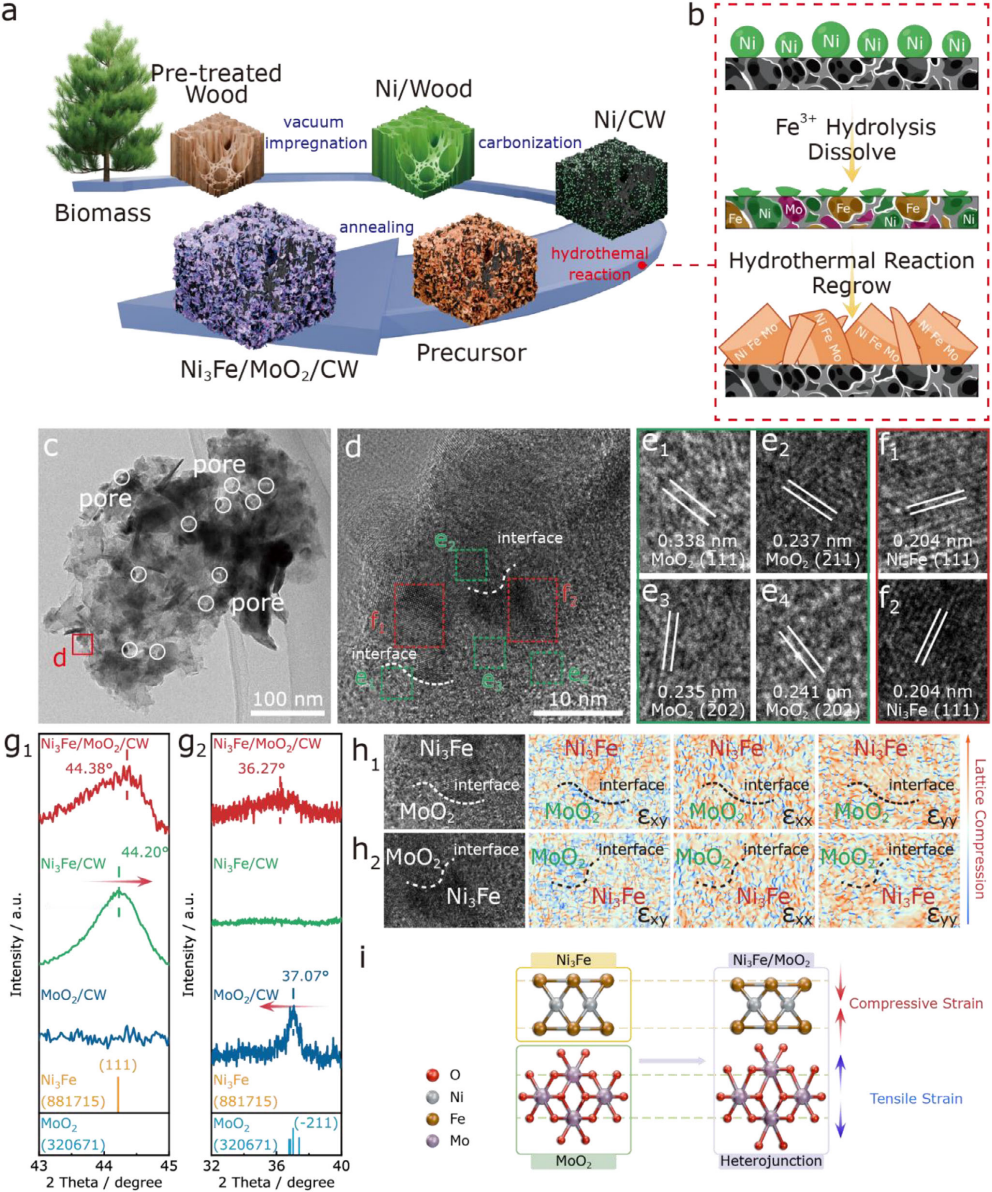
Schematic illustration of (a) the synthesis procedure of Ni_3_Fe/MoO_2_/CW and (b) the dissolution and regrowth process during Fe^3+^ hydrolysis; c–f) HRTEM images of Ni_3_Fe/MoO_2_/CW; g_1_–g_2_) XRD peak shifts of Ni_3_Fe/MoO_2_/CW, Ni_3_Fe/MoO_2_/CW, and MoO_2_/CW; h_1_‐h_2_) GPA‐based strain maps; i) Schematic of lattice evolution in the Ni_3_Fe/MoO_2_ heterostructure.

**Figures** **1**a and S1 (Supporting Information) schematically illustrate the stepwise synthesis of Ni_3_Fe/MoO_2_/CW. First, Ni^2+^ was uniformly introduced into the porous wood framework by vacuum impregnation to obtain Ni/Wood. After carbonization, the wood matrix was converted into conductive carbonized wood (CW), with Ni nanoparticles precipitated in‐situ, yielding Ni/CW.^[^ ^25^ ^]^ Subsequently, Fe^3+^ and MoO_4_^2−^ species were deposited onto Ni/CW via a hydrothermal process, during which the hydrolysis of Fe^3+^ promoted the dissolution‐reconstruction of Ni nanoparticles, while co‐deposited Fe^3+^ and MoO_4_^2−^ generated nanosheet structures on the CW channel walls (Figure 1b).^[^ ^26^ ^]^ Annealing in a H_2_/Ar atmosphere afforded the final Ni_3_Fe/MoO_2_/CW catalyst. XRD analysis (Figure S2, Supporting Information) revealed that the Precursor was mainly composed of NiMoO_4_ and Fe_2_(MoO_4_)_3_. Owing to the different reduction enthalpies of Ni, Fe, and Mo,^[^ ^27^, ^28^ ^]^ Ni and Fe were reduced to form Ni_3_Fe alloy, while MoO_4_^2−^ was partially reduced to MoO_2_, together constructing the Ni_3_Fe/MoO_2_ heterojunction.

The phase composition of Ni_3_Fe/MoO_2_/CW was confirmed by XRD (Figure S3, Supporting Information). Characteristic peaks at 44.4°, 51.8°, and 75.9° correspond to Ni_3_Fe (PDF#88‐1715), while broad reflections at 26.0°, 36.8°, and 54.6° are assigned to MoO_2_ (PDF#32‐0671). The peak near 26.0° also indicates the (002) plane of graphitic carbon (PDF#89‐8487). Control samples (Figures S4 and S5, Supporting Information) further confirm the successful synthesis of Ni_3_Fe/CW and MoO_2_/CW, respectively.

SEM analysis (Figure S6, Supporting Information) illustrates the morphological evolution of Ni_3_Fe/MoO_2_/CW during the synthesis process. Initially, Ni nanoparticles were uniformly anchored on the smooth CW channels, forming the Ni/CW intermediate (Figure S7, Supporting Information). Hydrothermal treatment induced nanoparticle dissolution and reconstruction, yielding a nanosheet‐like Precursor (Figure S8, Supporting Information). After annealing, the final catalyst preserved the nanosheet morphology while developing abundant pores and nanoparticles (Figures S9 and S10, Supporting Information), which are expected to facilitate electrolyte penetration and promote HER/OER activity. Subsequently, the morphology and lattice structure of Ni_3_Fe/MoO_2_/CW were characterized by transmission electron microscopy (TEM) and high‐angle annular dark‐field scanning TEM (HAADF‐STEM). As shown in Figures 1c and S11 (Supporting Information), the nanosheets contain abundant mesopores and micropores, which facilitates gas release and electrolyte infiltration, thereby promoting HER/OER.^[^ ^29^ ^]^ HRTEM images (Figures 1d–f and S12, Supporting Information) further display clear lattice fringes, with spacings of 0.204 nm assigned to Ni_3_Fe (111) and 0.237 (−211), 0.235 (−202), 0.241 (202), and 0.338 (−111) nm corresponding to MoO_2_ planes, confirming the formation of multiple Ni_3_Fe/MoO_2_ interfaces. Such heterojunctions not only provide abundant active sites but also optimize the adsorption free energy of reaction intermediates through electronic coupling,^[^ ^30^ ^]^ ultimately enhancing catalytic performance in water electrolysis. Comparison of the XRD patterns before and after heterojunction formation (Figure 1g_1_,g_2_) reveals a shift in the Ni_3_Fe (111) diffraction peak toward a higher angle, from 2θ = 44.20° to 44.38°, corresponding to a 0.18° shift. According to Bragg's law,^[^ ^31^ ^]^ this indicates a lattice compression in the Ni_3_Fe phase. In contrast, the MoO_2_ (−211) peak shifts toward a lower angle by 0.73°, from 2θ = 37.00° to 36.27°, suggesting a lattice expansion. These shifts arise from interfacial lattice strain induced by the mismatch in lattice constants between Ni_3_Fe and MoO_2_. To accommodate this mismatch, interfacial atoms undergo elastic deformation, which modulates the local electronic structure and promotes charge redistribution, beneficial for HER and OER catalytic activity.^[^ ^32^ ^]^

**Figure 2:**
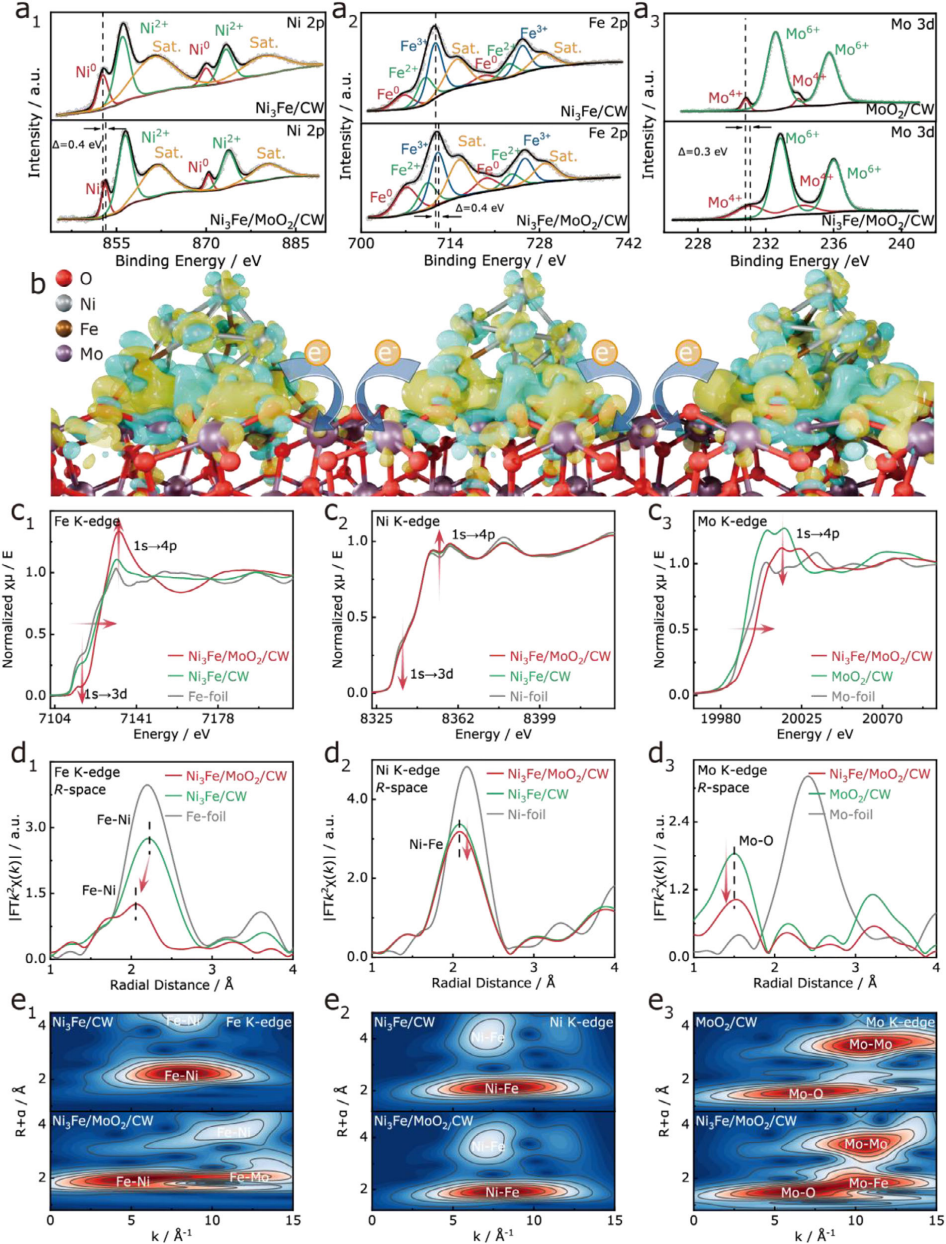
XPS spectra of a_1_‐a_3_) Ni 2p, Fe 2p, and Mo 3d of all catalysts; b) Schematic illustration of electron transfer in Ni_3_Fe/MoO_2_/CW; c_1_‐c_3_) XANES spectra at the Fe, Ni, and Mo *K*‐edges; d_1_–d_3_) Corresponding *R*‐space EXAFS spectra; e_1_–e_3_) Wavelet transform plots.

Strain mapping based on geometric phase analysis (GPA, Figure 1h_1_‐h_2_), derived from Figure 1d, further demonstrates that the strain is primarily concentrated near the interface: compressive strain is observed in the Ni_3_Fe region, whereas tensile strain dominates the MoO_2_ region. The schematic illustration in Figure 1i summarizes the interfacial strain‐induced modulation mechanism. This cooperative interfacial strain effect effectively tunes the *d*‐band center, optimizes the adsorption free energies of reaction intermediates, and thus simultaneously enhances the kinetics of both the OER and HER, endowing the Ni_3_Fe/MoO_2_ heterostructure with outstanding bifunctional electrocatalytic performance for water electrolysis. The uniform distribution of Ni, Fe, Mo, and O elements in the Ni_3_Fe/MoO_2_/CW catalyst was confirmed by energy‐dispersive X‐ray spectroscopy (EDS) and inductively coupled plasma mass spectrometry (ICP‐MS), along with the observation of a porous structure, as shown in Figure S13 (Supporting Information) and listed in Table S1 (Supporting Information), respectively.

### 2.2 Electronic Structure of Ni_3_Fe/MoO_2_/CW

X‐ray photoelectron spectroscopy (XPS) was employed to investigate the electronic structure of Ni_3_Fe/MoO_2_/CW. As shown in **Figure** **2**a, the Ni 2p, Fe 2p, and Mo 2p spectra display signals corresponding to Ni^0^/Ni^2+^, Fe^0^/Fe^2+^/Fe^3+^, and Mo^4+^/Mo^6+^, respectively, along with characteristic satellite peaks.^[^ ^33^, ^34^, ^35^, ^36^, ^37^, ^38^ ^]^ The presence of Ni^0^, Fe^0^, and Mo^4+^ confirms the coexistence of Ni_3_Fe alloys and MoO_2_, consistent with XRD results, while higher valence states likely originate from surface oxidation.^[^ ^39^, ^40^ ^]^ In addition, the O 1s and C 1s spectra (Figures S14 and S15, Supporting Information) reveal typical Metal─O, C─O, and O─C═O, as well as C═C/C─C, C─O, and O─C═O bonds,^[^ ^21^, ^41^ ^]^ respectively. Notably, distinct binding energy shifts were observed due to interfacial electronic coupling.^[^ ^42^ ^]^ Compared with individual Ni_3_Fe/CW and MoO_2_/CW, the Ni 2p and Fe 2p peaks in Ni_3_Fe/MoO_2_/CW shifted by 0.4 eV to higher binding energies, while Mo 2p peaks shifted by 0.3 eV higher and O 1s peaks shifted by 0.2 eV lower. These shifts suggest strong charge redistribution at the heterojunction interface, yielding electron‐rich MoO_2_ and electron‐deficient Ni_3_Fe regions (Figure 2b). Such interfacial electron transfer optimizes the catalyst's electronic structure, thereby boosting HER and OER activity.

More importantly, further insight into the dynamic changes in local coordination and electronic structure during the construction of the Ni_3_Fe/MoO_2_/CW heterostructure was obtained by X‐ray absorption fine structure (XAFS) measurements. Figure 2c_1_‐c_3_ shows the normalized X‐ray absorption near‐edge structure (XANES) spectra at the Fe K‐edge, Ni K‐edge, and Mo K‐edge of Ni_3_Fe/MoO_2_/CW, Ni_3_Fe/CW, and reference samples for comparison. Compared with Ni_3_Fe/CW, the Ni_3_Fe/MoO_2_/CW heterostructure exhibits a decrease in the pre‐edge peak intensities of Fe and Ni (corresponding to 1s→3d transitions), indicating enhanced 3d orbital hybridization and reduced local symmetry induced by interfacial coupling between Ni_3_Fe and MoO_2_. This structural distortion is conducive to the creation of active adsorption sites and the optimization of charge transport pathways. Additionally, the increased white‐line intensities at the Fe and Ni K‐edges (Figure 2c_1_–c_2_) suggest electron reduction of Ni_3_Fe, indicative of the formation of high‐valent active species. In contrast, the Mo K‐edge white‐line intensity decreases (Figure 2c_3_), implying electron accumulation on Mo species. This electron redistribution is consistent with XPS analysis, confirming the presence of significant interfacial charge transfer effects within the heterostructure.

To provide additional confirmation of the evolution of metal coordination environments, extended X‐ray absorption fine structure (EXAFS) data were Fourier‐transformed to obtain real‐space (R‐space) structural information (Figure 2d_1_–d_3_; Table S2, Supporting Information). Compared with Ni_3_Fe/CW, the Ni─Fe bond length in Ni_3_Fe/MoO_2_/CW is shortened, while the Mo─O coordination is weakened, indicating a strong interfacial interaction between the Ni_3_Fe alloy and MoO_2_. This interaction likely involves electronic coupling and structural rearrangement at the heterojunction, facilitating charge redistribution and local lattice modulation. Wavelet transform (WT) contour plots (Figure 2e_1_–e_3_) reveal the emergence of a new Fe─Mo scattering signal at high K‐values, accompanied by a diminished Fe─Ni coordination peak. This observation indicates that the incorporation of MoO_2_ restructures the local coordination environment of Ni_3_Fe, leading to the formation of Fe─Mo─O coordination units. Such hetero‐coordination is beneficial for modulating the interfacial electronic structure in a targeted manner.

### 2.3 Density Functional Theory Calculations

Density functional theory (DFT) calculations are widely employed to elucidate catalytic activity in HER and OER.^[^ ^43^ ^]^ To gain mechanistic insights, structural models of Ni_3_Fe/MoO_2_/CW and Ni_3_Fe/CW were constructed (Figures S16 and S17, Supporting Information). Guided by TEM results, a heterojunction was modeled by combining the Ni_3_Fe (111) plane with the MoO_2_ (−211) plane, incorporating lattice strain from XRD, followed by full relaxation.

**Figure 3:**
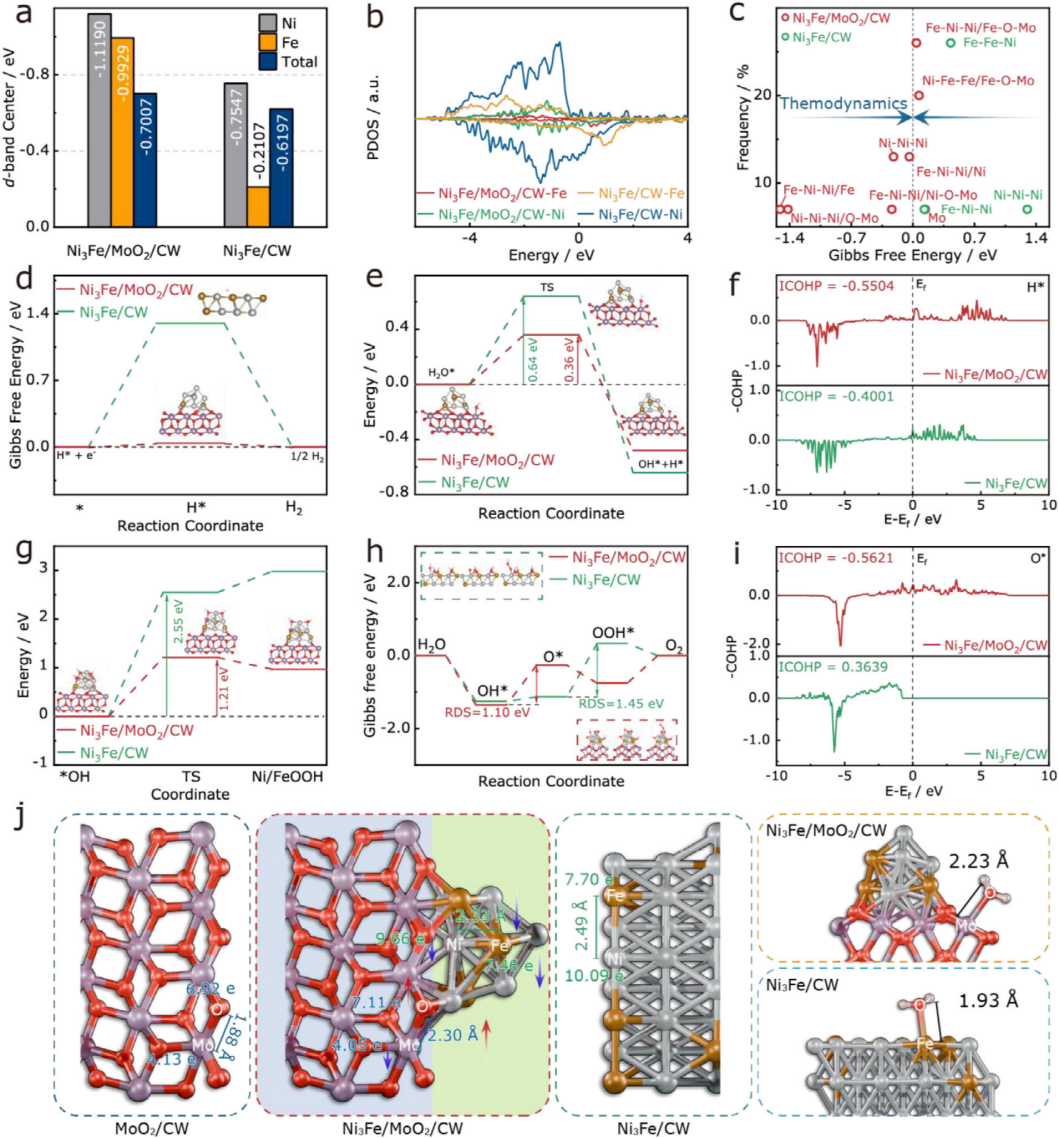
a) *d*‐band center and (b) DOS of catalysts; c) Frequency of active sites and Gibbs free energy; d) Gibbs free energy of H* for catalysts; e) Dissociation energy of water for catalysts; f) ICOHP of catalysts for H*; g) Reaction free energy of surface reconstruction on Ni_3_Fe/MoO_2_/CW and Ni_3_Fe/CW; h) Gibbs free energy of the OER process; i) ICOHP of catalysts for O*; j) Bond length and electronic structure variations after heterojunction formation.

The electronic modulation induced by the Ni_3_Fe/MoO_2_ interface was probed via charge density difference and Bader charge analyses (Figure S18 and Table S3, Supporting Information). Electron transfer from Ni, Fe, and Mo atoms to O atoms optimized the interfacial electronic configuration, consistent with XPS observations, and favored the generation of catalytically active species.^[^ ^44^ ^]^ Density of states (DOS) analysis (Figure S19, Supporting Information) confirmed the metallic nature and excellent conductivity of both systems,^[^ ^45^ ^]^ with *d* orbitals dominating near the Fermi level^[^ ^46^ ^]^ (Figures S20 and S21, Supporting Information). Notably, the *d*‐band centers of Ni and Fe in Ni_3_Fe/MoO_2_/CW shifted closer to the Fermi level relative to Ni_3_Fe/CW (**Figure** **3**a), suggesting improved adsorption of HER/OER intermediates in line with *d*‐band theory.^[^ ^47^, ^48^ ^]^ Projected DOS (PDOS) further revealed reduced electron density at Ni and Fe sites upon heterojunction formation (Figure 3b), corroborating an optimized electronic structure for enhanced catalytic activity.

In‐situ Raman spectroscopy revealed distinct behaviors for HER and OER. For the HER process (Figure S22, Supporting Information), no noticeable spectral changes were observed, indicating the absence of surface reconstruction. In contrast, during the OER (Figure S23, Supporting Information) process, the gradual emergence of Raman bands associated with NiOOH and FeOOH was detected as the potential increased, signifying surface reconstruction and the oxidation of Ni and Fe into high‐valence active sites. Given the structural stability observed under HER conditions, the correlation between the exposure frequency of different catalytic sites and their Gibbs free energy for hydrogen adsorption was systematically analyzed (Figure 3c). Evidently, under identical active site configurations, the hydrogen adsorption free energy value is closer to thermoneutral in the presence of MoO_2_. This observation underscores the beneficial role of MoO_2_ in modulating the local electronic environment and metal coordination, thereby enhancing the intrinsic catalytic activity of Ni_3_Fe sites. Figure 3d presents the lowest Gibbs free energy $\left(\Delta G_{H^{*}}\right)$ of H* on the surface of the catalysts. Notably, the $\Delta G_{H^{*}}$ value of Ni_3_Fe/MoO_2_/CW (0.04 eV) is closer to the ideal 0 eV compared to Ni_3_Fe/CW (1.30 eV). An optimal $\Delta G_{H^{*}}$ promotes a balanced adsorption/desorption of hydrogen on the catalyst surface, thereby significantly enhancing the HER kinetics.^[^ ^49^ ^]^

Furthermore, within the Volmer‐Heyrovsky mechanism, the water dissociation step typically requires overcoming a substantial energy barrier.^[^ ^50^ ^]^ As depicted in Figure 3e the water dissociation energy for Ni_3_Fe/MoO_2_/CW (0.36 eV) is considerably lower than that for Ni_3_Fe/CW (0.64 eV), indicating a reduced energy barrier for the Volmer step. This enhancement facilitates the rapid generation of H* intermediates, thereby accelerating the HER process. As illustrated in Figure S24 (Supporting Information), based on adsorption energy calculations, water molecules preferentially adsorb onto the MoO_2_ surface, which can be attributed to its higher oxygen affinity and the presence of undercoordinated Mo sites. Upon adsorption, the H_2_O molecule dissociates via O─H bond cleavage, forming surface‐bound hydroxyl (OH*) and hydrogen (H*) intermediates. The OH* species remains stably adsorbed on the MoO_2_ surface, while the generated hydrogen atom migrates to adjacent Ni_3_Fe sites. This interfacial reaction pathway exhibits a spatially separated adsorption configuration, wherein MoO_2_ facilitates water activation, and Ni_3_Fe offers favorable sites for hydrogen adsorption. The strong electronic coupling at the Ni_3_Fe/MoO_2_ interface effectively reduces the energy barrier for water dissociation, thereby enhancing both HER and OER performance under alkaline conditions. These findings suggest that the incorporation of MoO_2_ effectively modulates the HER reaction pathway of Ni_3_Fe alloy.

Further insight into the impact of electronic structure on the adsorption behavior of reaction intermediates was obtained by conducting crystal orbital Hamilton population (COHP) analysis of the bonding interactions between metal sites and adsorbed species.^[^ ^51^ ^]^ As illustrated in Figure 3f, the ICOHP integral for H* adsorption on Ni_3_Fe/MoO_2_/CW (−0.5504) is significantly higher than that of Ni_3_Fe/CW (−0.4001), revealing that the import of MoO_2_ strengthens the Ni/Fe─H bond. This enhancement not only facilitates H* adsorption on the catalyst surface but also promotes the cleavage of the H─OH bond during water dissociation, thereby synergistically optimizing both $\Delta G_{H^{*}}$ and water dissociation energy, thus, regulating the Volmer–Heyrovsky steps in HER.

For the OER process, the dynamic electrochemical evolution and regeneration of Ni and Fe active sites were investigated. Based on the initial structural model, surface reconstruction proceeds via OH^−^ adsorption, followed by the formation of hydroxides and their subsequent electrooxidation into hydroxylated oxide species.^[^ ^52^ ^]^ As shown in Figure 3g, calculations of the reconstruction reaction energy revealed that Ni_3_Fe/MoO_2_/CW (1.21 eV) exhibits a significantly lower energy barrier than Ni_3_Fe/CW (2.55 eV), indicating that Ni_3_Fe/MoO_2_ favors the generation of hydroxylated oxide sites at lower potentials, thereby enhancing catalytic activity. These findings were further corroborated by in situ Raman spectroscopy (Figure S23, Supporting Information).

Besides, to gain a greater understanding of the OER mechanism, the Gibbs free energy change (Δ*G*) of the rate‐determining step (RDS) in the adsorbate evolution mechanism (AEM) was analyzed to elucidate the energetic variations of different reaction intermediates,^[^ ^53^ ^]^ as shown in Figure 3h. The results reveal that for Ni_3_Fe/CW, the RDS corresponds to the O* → OOH* transition, with an associated Δ*G* of 1.45 eV. However, upon the introduction of MoO_2_ to form the Ni_3_Fe/MoO_2_ heterointerface, the RDS shifts to OH* → O*, with a significantly reduced Δ*G* of 1.10 eV. This transformation indicates that the heterojunction effectively alters the OER reaction pathway by lowering the energy barrier of the RDS, thereby enhancing the intrinsic catalytic activity. Meanwhile, the absolute ICOHP value for O* adsorption in Ni_3_Fe/MoO_2_/CW (−0.5621) is markedly greater than that in Ni_3_Fe/CW (−0.3639, Figure 3i). The strengthened Ni─O bond promotes greater O adsorption on the catalyst surface, causing the OER rate‐determining step to shift from O* → OOH* to OH* → O*, with the O* → OOH* step becoming a spontaneous process, effectively reducing the energy requirement for the adsorption evolution mechanism.

Finally, the variations in bond lengths were analyzed, as shown in Figure 3j. The presence of MoO_2_ significantly influenced the electronic environment and metal coordination of Ni_3_Fe during heterojunction formation. Specifically, as seen, the Mo─O bond in MoO_2_ is elongated from 1.88 Å to 2.30 Å, while the Ni─Fe bond in Ni_3_Fe is compressed from 2.49 to 2.33 Å, consistent with XRD and TEM analyses. Interestingly, during H_2_O adsorption, the bond length between water molecules and active metal sites increased from 1.93 to 2.23 Å, indicating a weakened H_2_O adsorption on Ni_3_Fe/MoO_2_/CW. This result suggests that the introduction of MoO_2_ effectively modulates interfacial H_2_O adsorption behavior, thereby creating a more favorable interfacial environment for the generation and transformation of key intermediates.

### 2.4 HER/OER and Water Electrolysis Performance of Ni_3_Fe/MoO_2_/CW

**Figure 4:**
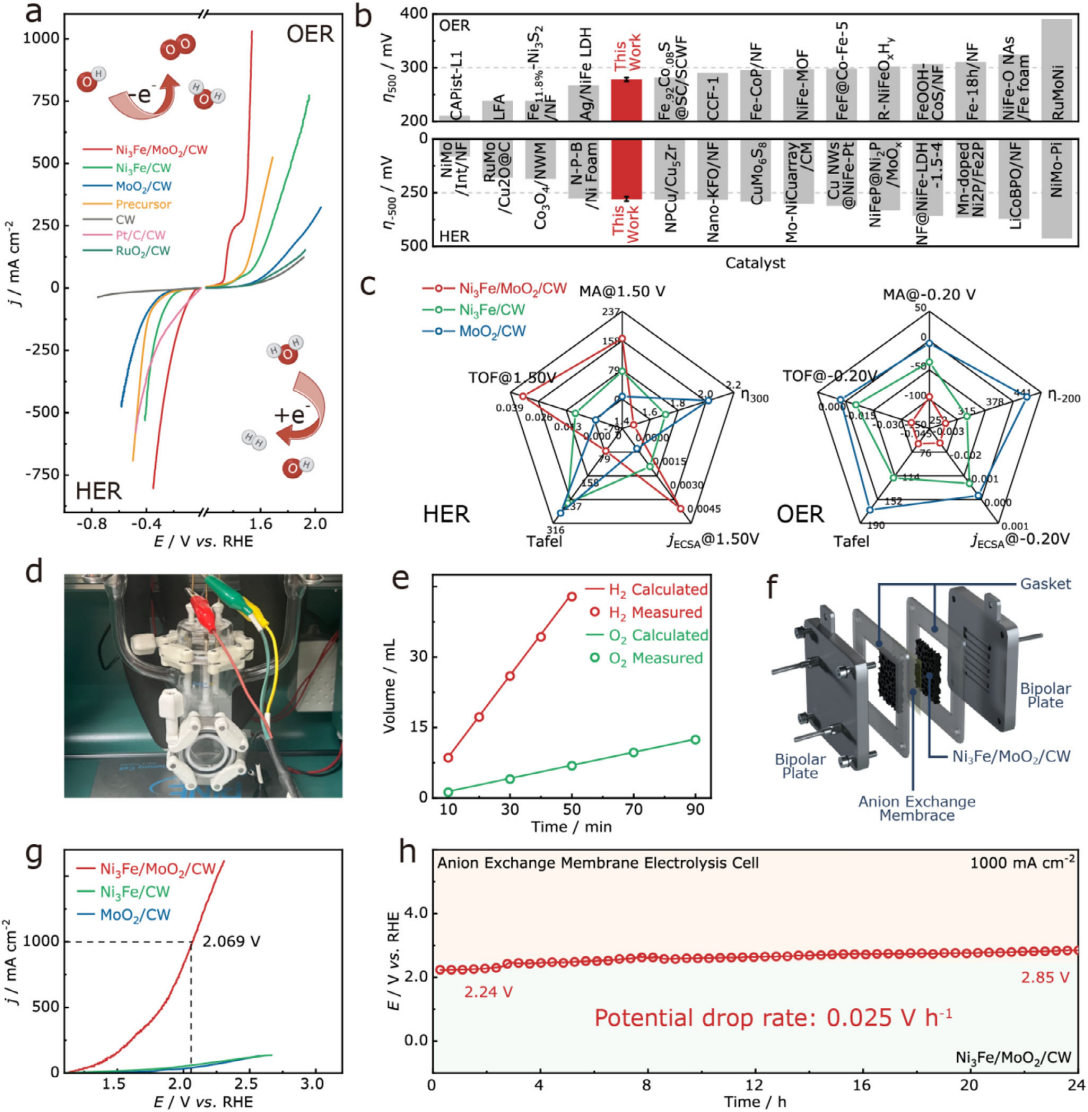
a) LSV curves of all catalysts; b) Overpotential comparison with reported catalysts;^[^ ^54^, ^55^, ^56^, ^57^, ^58^, ^59^, ^60^, ^61^, ^62^, ^63^, ^64^, ^65^, ^66^, ^67^, ^68^, ^69^, ^70^, ^71^, ^72^, ^73^, ^74^, ^75^, ^76^, ^77^ ^]^ c) Radar chart of the prepared catalysts; d) All‐glass online gas analysis system (GC); e) Theoretical vs. experimental H_2_/O_2_ amounts; f) Schematic of the anion exchange membrane electrolyzer; g) LSV curves and (h) stability of the AEM electrolyzer with the catalysts.

**Figures** **4**a and S25 (Supporting Information) shows the linear sweep voltammetry (LSV) curves of Ni_3_Fe/MoO_2_/CW, Ni_3_Fe/CW, MoO_2_/CW, Precursor, Pt/C/CW, and CW in 1.0 M KOH solution for both the HER and OER. Ni_3_Fe/MoO_2_/CW exhibits the highest catalytic activity among the tested catalysts, requiring only 45/342 mV (HER) and 251/306 (OER) mV overpotentials to achieve current densities of −10/−750 and 300/1000 mA cm^−2^, respectively. These values are significantly lower than those of Ni_3_Fe/CW, MoO_2_/CW, Precursor, and CW. Furthermore, the HER and OER activities of Ni_3_Fe/MoO_2_/CW are comparable to those observed for noble‐metal‐based Pt/C/CW and RuO_2_/C/CW, respectively, demonstrating that the synthesis of Ni_3_Fe/MoO_2_ heterojunction effectively enhanced electrocatalytic performance, outperforming most reported transition metal‐based catalysts, as shown in Figure 4b and in Tables S4 and S5 (Supporting Information). It is worth noting that the oxidation peaks observed during the OER process indicate the formation of hydroxide and oxide species on the surface of Ni_3_Fe/MoO_2_/CW. These species, together with Ni_3_Fe, serve as active sites and contribute to the enhanced OER activity,^[^ ^44^ ^]^ which is consistent with our preceding Raman analysis (Figure S23, Supporting Information) and DFT calculations (Figure 3e). Moreover, the effect of calcination temperature on the performance of Ni_3_Fe/MoO_2_/CW was systematically evaluated (Figures S26–S31, Supporting Information).

Tafel slope is a critical parameter for evaluating electrocatalytic kinetics, with lower values indicating more favorable reaction dynamics. In alkaline media, HER generally follows either the Volmer–Heyrovsky or Volmer–Tafel pathway.^[^ ^78^ ^]^ As shown in Figure S32a (Supporting Information), Ni_3_Fe/MoO_2_/CW exhibits a Tafel slope of 74 mV dec^−1^, suggesting that Ni_3_Fe/MoO_2_/CW follows the Volmer–Heyrovsky mechanism, and is significantly lower than that of Ni_3_Fe/CW (94 mV dec^−1^). This improvement is likely due to the introduction of MoO_2_, which modulates the adsorption and desorption behavior of H_2_O, H*, and OH* on the catalyst surface, reducing the water dissociation energy barrier and thereby accelerating the Volmer–Heyrovsky step. Furthermore, the Tafel slope of Ni_3_Fe/MoO_2_/CW is markedly lower than that of Precursor, MoO_2_/CW, and CW, and was comparable to that of Pt/C/CW. In the OER results depicted in Figure S32b (Supporting Information), Ni_3_Fe/MoO_2_/CW exhibits a Tafel slope of 75 mV dec^−1^, substantially lower than those of Ni_3_Fe/CW, MoO_2_/CW, Precursor, RuO_2_/C/CW, and CW. These results indicate that Ni_3_Fe/MoO_2_/CW possesses superior reaction kinetics for both HER and OER, facilitating rapid electrochemical conversion, due to the synergistic interfacial interaction between Ni_3_Fe and MoO_2_.

Electrochemical impedance spectroscopy (EIS) measurements further verified that the heterojunction substantially promoted interfacial charge transfer. At HER (−0.2 V) and OER (1.5 V) potentials, Ni_3_Fe/MoO_2_/CW exhibited markedly reduced charge transfer resistances (R_ct_) of 0.41 Ω and 0.72 Ω, respectively, significantly lower than those of Ni_3_Fe/CW (1.36 Ω/3.26 Ω) and MoO_2_/CW (4.92 Ω/42.59 Ω, Figures S33–S36, Supporting Information). This confirms that the heterojunction interface effectively accelerated electron transport and reduced the activation energy of the rate‐determining step, thereby enhancing overall catalytic performance.

As is known, the electrochemically active surface area (ECSA) is evaluated to quantify the number of accessible active sites.^[^ ^79^ ^]^ Cyclic voltammetry (CV) measurements in a non‐Faradaic potential range (0.15–0.45 V) revealed that Ni_3_Fe/MoO_2_/CW had a double‐layer capacitance (*C*_dl_) of 3.94 F cm^−1^, significantly higher than those of Ni_3_Fe/CW and MoO_2_/CW (Figure S37, Supporting Information), indicating a greater density of active sites. After ECSA normalization, the current densities at −0.20 V and 1.5 V for Ni_3_Fe/MoO_2_/CW (−0.00239/0.00407 mA cm^−2^) were considerably higher than those of Ni_3_Fe/CW and MoO_2_/CW, as illustrated in Figure S38 (Supporting Information), further confirming its superior intrinsic activity.

Figures S39–S42 (Supporting Information) and the radar chats (Figure 4c) provide a comprehensive comparison of key performance metrics, including ECSA‐normalized current density (*j*_ECSA_), turnover frequency (TOF), mass activity (MA), overpotential, and Tafel slope. Ni_3_Fe/MoO_2_/CW outperformed both Ni_3_Fe/CW and MoO_2_/CW across all evaluated parameters, underscoring its superior catalytic activity for both HER and OER. This enhancement can be attributed to the optimized electronic structure and contracted Ni─Fe bonds within the heterojunction, which together increases more active sites, facilitates more favorable adsorption/desorption of reaction intermediates, and accelerates charge transfer kinetics during the water electrolysis process.

Long‐term stability is crucial for practical applications. As shown in Figure S43 (Supporting Information), Ni_3_Fe/MoO_2_/CW maintained stable performance over 120 h at high current densities of −500 mA cm^−2^ (HER) and 400 mA cm^−2^ (OER), with negligible potential variation (HER: 0.0017 V h^−1^; OER: 0.001 V h^−1^) and composition change (Table S6, Supporting Information). Moreover, the LSV curves before and after the stability test remained nearly unchanged (Figures S44 and S45, Supporting Information), confirming its excellent durability. SEM (Figure S46, Supporting Information) further demonstrates that, owing to the in‐situ grown self‐supporting architecture, the porous nanosheet structure of Ni_3_Fe/MoO_2_/CW remains intact even after prolonged electrolysis, indicating excellent mechanical robustness. XRD analysis after 120 h of continuous HER/OER operation revealed no significant changes in the phase composition, confirming the retention of the Ni_3_Fe and MoO_2_ heterostructure. Additionally, XPS analysis showed a slight increase in the proportion of low‐valence states (Ni^0^, Fe^0^, Mo^4+^) after HER testing and a moderate rise in high‐valence states (Ni^2+^, Fe^2+^/Fe^3+^, Mo^6+^) following OER testing (Figures S47–S50 and Table S7, Supporting Information). However, the overall chemical state distribution remained largely unchanged, confirming the catalyst's strong chemical stability under operational conditions.

To evaluate the industrial feasibility of Ni_3_Fe/MoO_2_/CW, an electrolyzer was assembled using Ni_3_Fe/MoO_2_/CW as both the anode and cathode, with 1.0 M KOH solution as the electrolyte. Building upon prior HER/OER performance analyses and DFT‐derived mechanistic insights, a plausible water electrolysis pathway under a two‐electrode configuration is proposed (Figure S51, Supporting Information). During the HER process, OH^−^ preferentially adsorbs onto Mo sites, while H* occupies a Ni─Ni─Fe vacancy site, accelerating the Volmer step. The subsequent Heyrovsky step proceeds via reaction of H with H_2_O, yielding H_2_ that desorbs efficiently from the interface. For the OER process, following the adsorbate evolution mechanism (AEM), OH^−^ initially occupies the same vacancy site and reacts to form O species at the Ni sites, identified as the rate‐determining step. Continued OH^−^ attack eventually leads to O_2_ evolution. The formation of the Ni_3_Fe/MoO_2_ heterojunction effectively modulates both HER (Volmer–Heyrovsky) and OER (AEM) pathways, achieving synergistic enhancement of overall electrocatalytic performance.

As shown in Figure S52 (Supporting Information), the assembled electrolyzer delivered 100 mA cm^−2^ at a low voltage of 1.55 V, surpassing the Pt/C/CW||RuO_2_/C/CW benchmark (1.99 V), the Ni_3_Fe/MoO_2_‐powder/CW, and many previously reported catalysts (Figure S53 and Table S8, Supporting Information). After the LSV test, further SEM analysis revealed significant aggregation and detachment of the Ni_3_Fe/MoO_2_ components in the Ni_3_Fe/MoO_2_‐powder/CW system (Figure S54, Supporting Information). In contrast, the Ni_3_Fe/MoO_2_/CW catalyst effectively suppressed metal site aggregation and detachment, owing to its in‐situ grown architecture and strong interfacial interactions with the wood‐derived carbon matrix. Notably, after 120 h of continuous operation at 100 mA cm^−2^, the voltage increased by only 0.13 V, underscoring its exceptional durability for industrial applications (Figure S55, Supporting Information). To quantitatively assess the gas evolution, an all‐glass automated online trace gas analysis system coupled with gas chromatography was employed, as shown in Figure 4d. The H_2_ and O_2_ generation rates exhibited a linear correlation with time and were closely aligned with theoretical values (Figure 4e), demonstrating high faradaic efficiency.

To demonstrate its feasibility under practical alkaline industrial environments, an anion exchange membrane electrolyzer was assembled using Ni_3_Fe/MoO_2_/CW as both electrodes, as shown in Figure 4f. The device is tested in 6.0 M KOH at 60 °C, where LSV measurements reveal that is required only 2.069 V to sustain a current density of 1000 mA cm^−2^, significantly lower than that of Ni_3_Fe/CW and MoO_2_/CW (Figure 4g). This result highlights the potential of Ni_3_Fe/MoO_2_/CW as an energy‐efficient electrocatalyst for industrial water electrolysis. To assess long‐term durability, the Ni_3_Fe/MoO_2_/CW‐based anion exchange membrane electrolyzer is subjected to continuous operation at 1000 mA cm^−2^ in an alkaline environment. As shown in Figure 4h, the system maintains stable performance over 24 h with minimal voltage fluctuations (0.025 V h^−1^) and composition change (Table S6, Supporting Information), confirming its robustness under industrial electrolysis conditions.

The above outstanding stability and efficiency can be attributed to the synergistic effects of the Ni_3_Fe/MoO_2_ heterojunction, which not only increases the density of active sites but also enhances intrinsic catalytic activity by modulating the local electronic structure and Ni─Fe bonds through strong interfacial coupling. Additionally, the unique porous nanosheet morphology imparts superhydrophilic/superhydrophobic properties, facilitating rapid gas bubble release and improving mass transport at high current densities. Overall, the successful fabrication of Ni_3_Fe/MoO_2_/CW introduces a novel approach for designing wood‐carbon‐based catalysts with promising potential for industrial water electrolysis applications.

### 2.5 Hydrophilic/Gas‐Phobic Properties of Ni_3_Fe/MoO_2_/CW

The remarkable stability of Ni_3_Fe/MoO_2_/CW can be attributed to the synergistic effects of its i) 3D layered substrate, ii) porous nanosheet morphology, and iii) superhydrophilic/superaerophobic properties. These structural features not only prevent active site blockage but also enhance mass transport at the gas‐liquid interface; factors that are particularly critical for industrial‐scale water electrolysis. Indeed, the catalytic performance in HER and OER is strongly governed by the interactions among: i) the solid catalyst, ii) the liquid electrolyte, and iii) the gaseous reaction products.^[^ ^38^, ^80^ ^]^ Accumulation of gas bubbles on the catalyst surface can hinder mass transfer by decreasing the contact area with the electrolyte, elevating interfacial resistance, and slowing reaction kinetics. Therefore, the tailored surface wettability and gas‐repellent nature of Ni_3_Fe/MoO_2_/CW play a pivotal role in maintaining efficient catalytic activity under high current densities.

**Figure 5:**
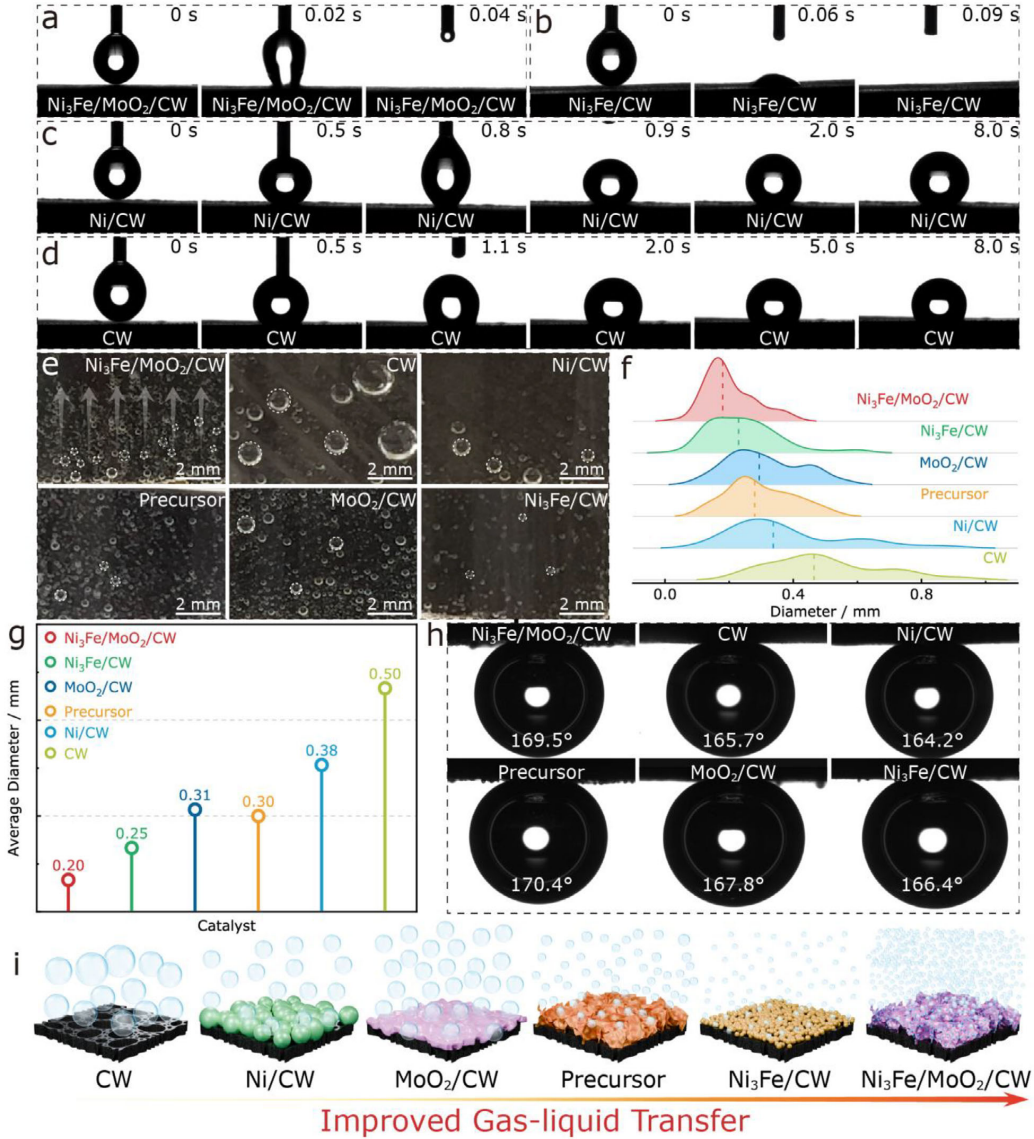
Contact angle of (a) Ni_3_Fe/MoO_2_/CW, b) Ni_3_Fe/CW, c) Ni/CW, and (d) CW; e) Photos of bubble behavior; f) Bubble diameter distribution; g) Average bubble diameter; h) Underwater gas‐bubble contact angle of Ni_3_Fe/MoO_2_/CW, CW, Ni/CW, Precursor, MoO_2_/CW, and Ni_3_Fe/CW; i) Illustration of bubble release.

The wettability of the catalysts was assessed by contact angle measurements. Ni_3_Fe/MoO_2_/CW exhibited superhydrophilicity, with water droplets disappearing within 0.04 s (**Figures** **5**a and S56, Supporting Information). FTIR analysis (Figure S57, Supporting Information) revealed abundant surface hydroxyl (O─H) and carbonyl (C═O) groups introduced under solvothermal conditions, which, together with the hierarchical porous nanosheet architecture, greatly enhanced wettability. Consequently, Ni_3_Fe/CW also showed superhydrophilicity with a droplet absorption time of 0.09 s (Figures 5b and S56, Supporting Information), while the Precursor absorbed water within 0.6 s (Figures S56 and S58, Supporting Information). In contrast, CW, Ni/CW, and MoO_2_/CW were hydrophobic, with contact angles of 120.5°, 134.4°, and 136.2° (Figures 5c,d, S56, and S59, Supporting Information). Combined FTIR and SEM analyses (Figures S60–S61, Supporting Information) confirm that the superhydrophilicity of Ni_3_Fe/MoO_2_/CW originates from both enriched oxygen‐containing functional groups and the porous nanosheet arrays, which synergistically promotes electrolyte penetration and gas‐liquid‐solid interactions during electrolysis.

To assess gas evolution dynamics, real‐time imaging was captured to observe hydrogen bubble release behavior at a current density of −300 mA cm^−2^. As illustrated in Figure 5e and Movies S1–S6 (Supporting Information), Ni_3_Fe/MoO_2_/CW exhibited the highest H_2_ bubble density on its surface compared to CW, Ni/CW, Precursor, MoO_2_/CW, and Ni_3_Fe/CW. Moreover, the bubble size distribution in Figure 5f revealed that H_2_ bubbles on Ni_3_Fe/MoO_2_/CW were predominantly in the 0–0.4 mm range, with an average diameter of 0.20 mm, significantly smaller than those on CW (0.50 mm), Ni/CW (0.38 mm), Precursor (0.30 mm), MoO_2_/CW (0.31 mm), and Ni_3_Fe/CW (0.25 mm), as summarized in Figure 5g. Such improvement is closely related to the role of the carbonized wood support, which provides an interconnected porous network to accelerate electrolyte infiltration and bubble detachment, while simultaneously offering a conductive scaffold to facilitate charge transport. These synergistic effects greatly contribute to the superaerophobic behavior and the enhanced catalytic performance of Ni_3_Fe/MoO_2_/CW. These results indicate that bubble accumulation was effectively minimized on Ni_3_Fe/MoO_2_/CW during the reaction process, as small bubbles formed and detached rapidly from the surface.

Additionally, the aerophobicity of the catalysts toward O_2_ was evaluated via underwater gas‐bubble contact angle measurements. As illustrated in Figure 5h, Ni_3_Fe/MoO_2_/CW exhibited a contact angle of 169.5°, further enhancing aerophobic properties compared to CW (165.7°) and surpassing those of Ni/CW (164.2°), MoO_2_/CW (167.8°), and Ni_3_Fe/CW (166.4°), though lower than that of Precursor (170.4°), which also featured a nanosheet structure. Previous studies have demonstrated that nanosheet architectures with controlled roughness not only facilitate electrolyte infiltration through capillary action but also reduce viscous drag at the three‐phase contact line, thereby accelerating bubble detachment.^[^ ^81^, ^82^ ^]^ Consequently, the superhydrophilic/superaerophobic characteristics of Ni_3_Fe/MoO_2_/CW ensured efficient electrolyte exchange and prevented bubble accumulation, ultimately enhancing the HER/OER processes, as shown in Figure 5i. The observed superhydrophilic/superaerophobic surface characteristics correlate directly with the catalyst's performance under high current densities. By minimizing bubble accumulation and ensuring continuous exposure of active sites, Ni_3_Fe/MoO_2_/CW maintained stable HER/OER operation at industrial‐relevant current densities, demonstrating that the surface wettability and bubble detachment behavior are key contributors to its long‐term electrochemical stability.

Interestingly, compared to the nanoparticle morphology of Ni_3_Fe/CW (Figure S60, Supporting Information) and the irregular bulk structure of MoO_2_/CW (Figure S61, Supporting Information),Ni_3_Fe/MoO_2_/CW displayed porous nanosheet‐nanoparticle architecture and exhibited type IV isotherms, indicating the presence of both micropores and mesopores within the catalyst, larger specific surface area (167.50 m^2^/g) and micropore volume (0.053 cm^3^ g^−1^) than those of Ni_3_Fe/CW (S_BET_ = 109.08 m^2^ g^−1^, V_micro_ = 0.022 cm^3^ g^−1^) and MoO_2_/CW (S_BET_ = 83.27 m^2^ g^−1^, V_micro_ = 0.016 cm^3^ g^−1^), as shown in Figure S62 and Table S9 (Supporting Information). The large specific area of Ni_3_Fe/MoO_2_/CW can enhance electrolyte infiltration and facilitate gas bubble release,^[^ ^83^ ^]^ which benefits the catalytic performance of water electrolysis at high current densities. Additionally, the effect of calcination temperature on the specific surface area of Ni_3_Fe/MoO_2_ was investigated (Figure S63 and Table S8, Supporting Information). Among the tested conditions, the sample calcined at 450 °C exhibited the highest specific surface area, thereby exposing more active sites for catalysis. Meanwhile, the BET surface area of Ni_3_Fe/MoO_2_/CW was measured after the stability test (Figure S64, Supporting Information), showing a negligible change, which indicates its excellent structural stability.

## 3 Conclusion

In this work, we developed a superhydrophilic/superaerophobic Ni_3_Fe/MoO_2_/CW catalyst by constructing a Ni_3_Fe‐MoO_2_ heterojunction supported on 3D wood‐derived carbon, which simultaneously addresses the challenges of sluggish HER/OER kinetics and inefficient gas‐liquid mass transfer under industrial‐level current densities. XAFS and DFT calculations revealed that MoO_2_ introduction induces strong interfacial coupling, leading to electron transfer from Ni_3_Fe to MoO_2_, a downward shift in the *d*‐band center of Ni/Fe, and a contraction of the Ni─Fe bond. These effects collectively optimize the electronic structure and metal coordination environment, thereby promoting H─OH bond dissociation and facilitating H* adsorption/desorption to accelerate the Volmer–Heyrovsky mechanism during the HER process. For OER, the strengthened Ni─O─Mo interfacial bonding effectively lowers the energy barrier of the rate‐determining step (from 1.462 to 1.092 eV), enhancing the kinetics of the adsorption evolution mechanism (AEM). As a result, the catalyst delivers outstanding bifunctional activity, with low overpotentials (HER: *η*_−10/−750_ = 45/342 mV; OER: *η*_300/1000_ = 251/306 mV) and favorable kinetics (Tafel slopes: 74 mV dec^−1^ for HER, 75 mV dec^−1^ for OER).

Furthermore, benefiting from its hierarchical nanosheet‐nanoparticle structure, Ni_3_Fe/MoO_2_/CW exhibits an enlarged specific surface area and robust gas‐liquid‐solid interface properties, which ensure efficient electrolyte infiltration and rapid gas bubble detachment. Impressively, in an anion exchange membrane electrolyzer, Ni_3_Fe/MoO_2_/CW sustains a high current density of 1000 mA cm^−2^ at just 2.069 V, with excellent operational stability over 24 h. Overall, this study presents a MoO_2_‐driven interfacial electronic modulation strategy that unlocks the full potential of Ni_3_Fe‐based catalysts for practical, high‐rate water electrolysis, offering a sustainable pathway by integrating renewable biomass‐derived carbon supports.

## 4 Experimental Section

### Synthesis of Ni_3_Fe/MoO_2_/CW

The Ni_3_Fe/MoO_2_/CW catalyst was synthesized through a multistep process using wood‐derived carbon as the support. The wood chips were first pretreated in deionized (DI) water at 80 °C for 10 h, followed by delignification in a NaClO_2_/CH_3_COOH solution at 75 °C for 2 h. The pretreated wood chips were then vacuum‐impregnated with a Ni(NO_3_)_2_·6H_2_O solution (0.14 mmol mL^−1^) for 8 h and subsequently carbonized under Ar at 800 °C to yield Ni/CW. Thereafter, Ni/CW was immersed in an aqueous solution containing Na_2_MoO_4_ (400 mg) and Fe(NO_3_)_3_·9H_2_O (298 mg), followed by solvothermal treatment at 180 °C for 8 h. After washing and drying, the resulting precursor was annealed in H_2_/Ar (5% H_2_) at 450 °C to afford Ni_3_Fe/MoO_2_/CW. For comparison, Ni_3_Fe/CW was prepared following the same procedure but without the addition of Na_2_MoO_4_, while MoO_2_/CW was obtained by replacing Ni(NO_3_)_2_·6H_2_O with Na_2_MoO_4_. To investigate the role of the carbon substrate, untreated wood chips were directly carbonized under Ar at 800 °C, and the resulting material was designated as CW. In addition, RuO_2_/C/CW, Ni_3_Fe/MoO_2_‐powder/CW, and Pt/C/CW were prepared by loading corresponding commercial catalysts onto wood‐derived carbon using 5 wt.% Nafion as the binder.

### Material Characterization

The structural and morphological properties of the catalysts were comprehensively characterized using multiple analytical techniques. X‐ray diffraction (XRD) patterns were obtained using a Rigaku MinFlex600 diffractometer with Cu K_α_ radiation (λ = 1.54178 Å) to determine crystallographic phases. The surface morphology was visualized by scanning electron microscopy (SEM) using Hitachi SU8220 and Zeiss Sigma500. The elemental composition and oxidation states of Ni, Fe, and Mo were analyzed via X‐ray photoelectron spectroscopy (XPS) (Thermo Scientific ESCALAB 250Xi), while the bulk elemental content was quantified using inductively coupled plasma mass spectrometry (ICP‐MS) (Agilent 7500ce). To investigate the nanostructure, transmission electron microscopy (TEM) was performed using a Thermo Fisher Talos F200X G2. High‐angle annular dark‐field scanning TEM (HAADF‐STEM) and energy‐dispersive X‐ray spectroscopy (EDS) mapping were conducted using the same TEM instrument to examine element distribution. The hydrophilic and aerophobic properties of the catalysts were assessed by measuring the water contact angle (Krüss DSA25) and the underwater bubble contact angle (Lauda Scientific LSA100), respectively. The specific surface area and porosity were analyzed using a Micromeritics ASAP 2420 analyzer based on the Brunauer–Emmett–Teller (BET) method. Additionally, Fourier transform infrared spectroscopy (FT‐IR) was carried out using a Bruker Tensor II spectrometer to identify functional groups present in the catalysts.

X‐ray absorption fine structure (XAFS) measurements of Fe, Ni, and Mo *K*‐edges were conducted at beamline BL14W1 of the Shanghai Synchrotron Radiation Facility (SSRF), utilizing Si (111) double‐crystal monochromators. Prior to data acquisition, the powder samples were pelletized into 1 cm diameter discs and sealed with Kapton tape to ensure consistent geometry and prevent contamination. All XAFS spectra were collected at ambient temperature using a Bruker 5040 four‐channel Silicon Drift Detector (SDD). Extended X‐ray absorption fine structure (EXAFS) data at the Fe, Ni, and Mo *K*‐edges were acquired in transmission mode. Consistency between successive scans was confirmed by the negligible variation in both spectral features and peak positions. Reference spectra of metallic Fe, Ni, and Mo foils were also recorded under identical conditions for calibration. Data reduction and fitting were carried out using the Athena and Artemis software packages.

### Electrochemical Measurements

The electrocatalytic performance of the synthesized materials was evaluated for both the hydrogen evolution reaction (HER) and oxygen evolution reaction (OER) using a conventional three‐electrode system at 30 °C. The working electrode was prepared by depositing the catalyst on a conductive substrate, while a Hg/HgO electrode served as the reference electrode, and a graphite rod was used as the counter electrode. Electrochemical measurements were performed in 1.0 M KOH alkaline electrolyte. All measured potentials were converted to the reversible hydrogen electrode (RHE) scale for consistency using the equation:^[^ ^84^ ^]^

(1) $$E\left(vs.\;\mathrm{RHE}\right)=E\left(vs.\;\mathrm{Hg}/\mathrm{HgO}\right)+0.098+0.0591\times pH$$

Linear sweep voltammetry (LSV) was recorded using an electrochemical workstation (DongHua DH7001B) at a scan rate of 5 mV s^−1^, with iR compensation applied (where i represents the current and R denotes the solution resistance). The Tafel slope was determined from the LSV data using the equation:^[^ ^85^ ^]^

(2) $$\eta =b\;\log\;\left|j\right|+a$$

where *η* represents the overpotential, *b* is the Tafel slope, and *j* denotes the current density.

Electrochemical impedance spectroscopy (EIS) was conducted over a frequency range of 0.1 Hz to 10^5^ Hz with an amplitude of 5 mV to investigate charge transfer resistance. Additional electrochemical parameters, including the electrochemical active surface area (ECSA), turnover frequency (TOF), and mass activity, were calculated and provided in the Supporting Information.

## Conflict of Interest

The authors declare no conflict of interest.

## Author Contributions

L.L. and H.X. contributed equally to this work. L.L. performed in conceptualization, data curation, investigation, writing–original draft, formal analysis. H.X. performed in conceptualization, methodology, writing–original draft. G.Q. performed in formal analysis, funding acquisition, conceptualization, validation, writing–review and editing. X.C. performed in investigation, data curation, validation. J.L. performed in software, data curation. Y.X. performed in methodology, data curation. R.Z. performed in formal analysis, methodology. D.M. performed in software, data curation. J.C. performed in conceptualization, writing–review and editing, methodology. P.T. performed in conceptualization, validation, writing–review and editing.

## Supporting information



Supporting Information

Supplemental Movie 1

Supplemental Movie 2

Supplemental Movie 3

Supplemental Movie 4

Supplemental Movie 5

Supplemental Movie 6

## Data Availability

The data that support the findings of this study are available from the corresponding author upon reasonable request.
