# Supplementary material for: MoO2‐Mediated Ni─Fe Bond Contraction and Electronic Modulation in Ni3Fe Alloy for Efficient Water Electrolysis at High‐Current‐Densities
Source: Adv Mater. 2025 Sep 30;38(3):e12658. doi: 10.1002/adma.202512658 (PMC12801374; doi:10.1002/adma.202512658)
Supplement: Supplementary file 1 — Supporting Information [file ADMA-38-e12658-s001.docx]

Supporting Information

MoO_2_-Mediated Ni−Fe Bond Contraction and Electronic Modulation in Ni_3_Fe Alloy for Efficient Water Electrolysis at High-Current-Densities

Liancen Li^+^, Haotian Xu^+^, Guangfu Qian*, Xinyu Cao, Jiawei Li, Yihao Xu, Ruyu Zhang, Douyong Min,

Guangxi Key Laboratory of Clean Pulp & Papermaking and Pollution Control, School of Light Industry and Food Engineering, Guangxi University, Nanning 530004, China

qianguangfu@gxu.edu.cn

Jinli Chen*,

State Key Laboratory of Materials Processing & Die and Mould Technology, School of Materials Science and Engineering, Huazhong University of Science and Technology, Wuhan 430074, China

chenjinli@hust.edu.cn

Panagiotis Tsiakaras*,

Laboratory of Alternative Energy Conversion Systems, Department of Mechanical Engineering, School of Engineering, University of Thessaly, 1 Sekeri Street, Pedion Areos 38834, Greece.

tsiak@uth.gr

+Co-first authors

*Corresponding authors: qianguangfu@gxu.edu.cn (G. Qian); chenjinli@hust.edu.cn (J. Chen); tsiak@uth.gr (P. Tsiakaras).

1. **Experimental Section Contd.**

**Calculation of ECSA**

ECSA can be calculated by the following formula:

$$j_{\text{ECSA}}\text{= }j\times\text{40μF/C}_{\text{dl}}$$

Where j is current density (A cm^−2^); The specific capacitance (C_s_) of a flat surface is generally between 20 and 60 μF cm^−2^, and 40 μF cm^−2^ is used in this work. The double layer capacitance (*C*_dl_) can be obtained by the slope of the fitted straight line in **Figure S29**.

**Calculation of TOF**

TOF is calculated on the assumption that all the metal ions in the samples are catalytically active according to the following formula:

$$\text{TOF=}\frac{\text{j}\times\text{A}}{\alpha\times\text{F}\times\text{n}}$$

Where j is current density (A cm^−2^); A is the area of the working electrode (1.0 cm^2^); α is the number of moles of electrons that produce 1.0 mol of H_2_ and O_2_, which is 2 and 4 in HER and OER, respectively; F is Faraday’s constant (96485.33 C mol^−1^); n is the number of moles of metal content in the electrode. The metal element content of each catalyst is acquired by ICP-MS (**Table S2**).

**Calculation of MA**

The formula for calculating mass activity is:

$$\text{MA}=\text{j/m}$$

Where j is current density (A cm^−2^); m is mass loading.

**DFT Calculation**

All computations were carried out with the Vienna Ab Initio Simulation Package (VASP) employing the projector augmented wave (PAW) approach. The generalized gradient approximation (GGA) with the Perdew-Burke-Ernzerhof (PBE) functional was adopted to describe exchange-correlation effects, and van der Waals interactions were corrected using Grimme’s D3 scheme. A kinetic energy cutoff of 400 eV was chosen for the plane-wave basis, and the electronic self-consistent loop was converged to 10^-5^ eV. To avoid spurious interactions between neighboring slabs, a vacuum space of 15 Å was introduced. Structural optimizations were performed until the total energies reached their minima, yielding relaxed configurations. The Gibbs free energy change ($\Delta G$) of each step was evaluated under thermodynamic equilibrium conditions as $\Delta G=\Delta E+\Delta E_{ZPE}-T\Delta S$, where $\Delta E$ corresponds to the electronic energy obtained from DFT, $\Delta E_{ZPE}$ is the zero-point energy correction estimated within the harmonic oscillator approximation, and $\Delta S$ denotes the entropy contribution derived from vibrational analysis at 298.15 K. During geometry optimizations along the reaction pathway, the residual force acting on each atom was constrained below 0.01 eV Å^-1^. After repeated relaxations, the optimized structures of Ni_3_Fe/MoO_2_/CW and Ni_3_Fe/CW were identified.

1. **Supplementary Figures**


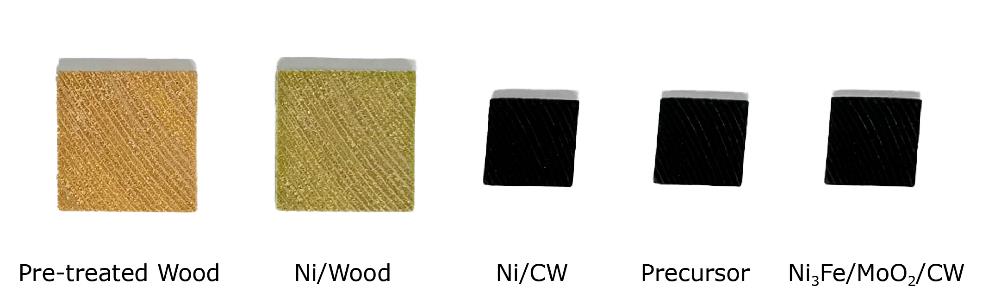


**Figure S1.** Photographs showing the step-by-step synthesis process of the catalyst.


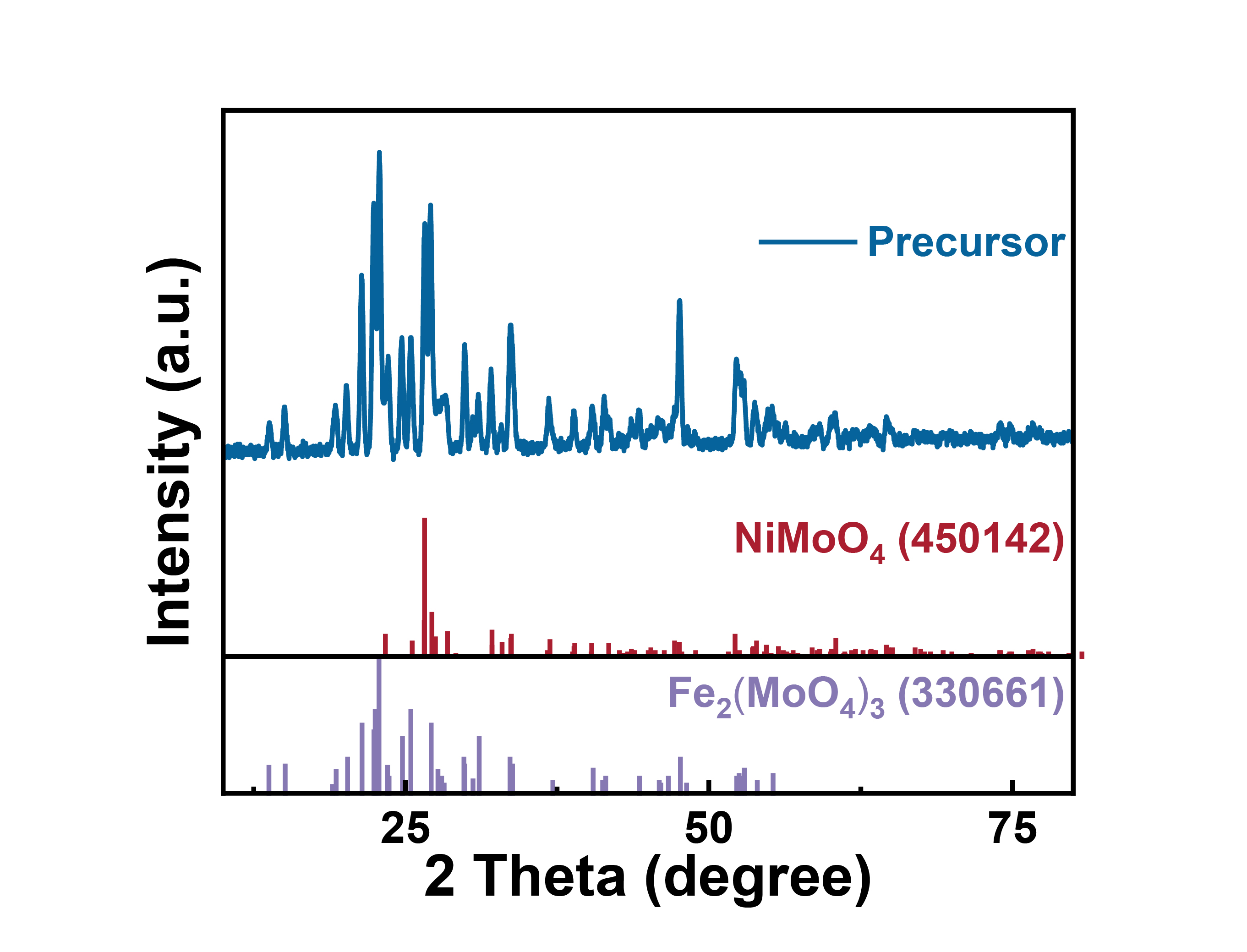


**Figure S2.** XRD pattern of Precursor.


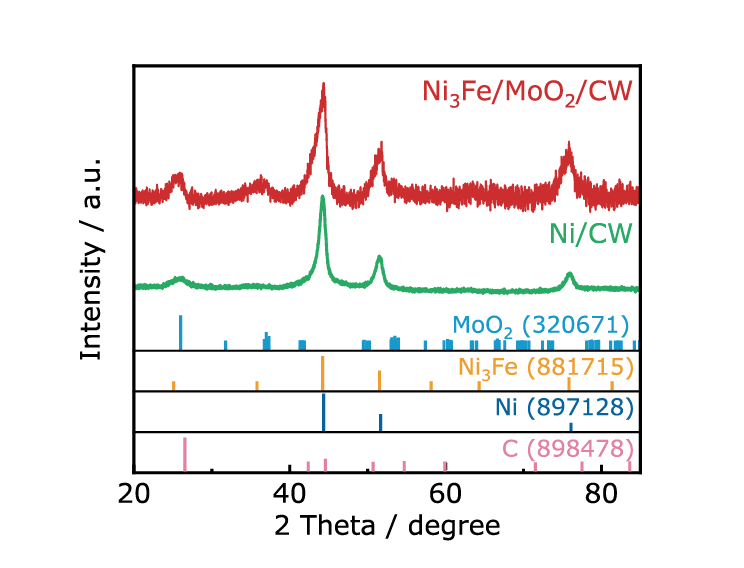


**Figure S3.** XRD pattern of Ni/CW and Ni_3_Fe/MoO_2_/CW.

**Figure S4.** XRD pattern of Ni_3_Fe/CW.

**Figure S5.** XRD pattern of MoO_2_/CW.


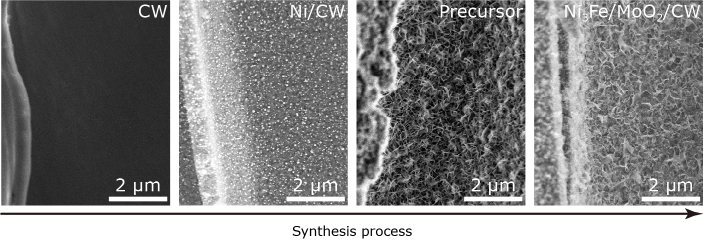


**Figure S6.** SEM images of CW, Ni/CW, Precursor, and Ni_3_Fe/MoO_2_/CW.

**
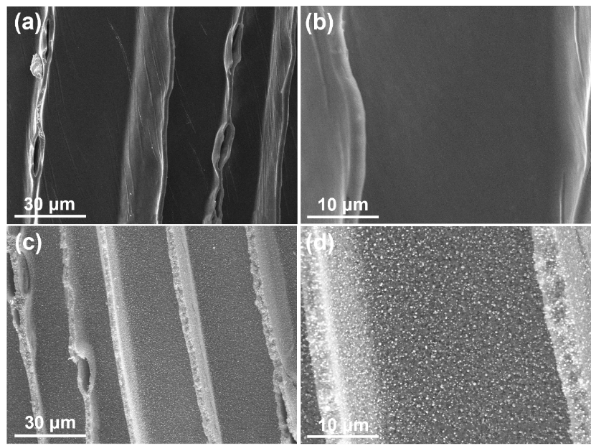
**

**Figure S7.** SEM images of (a-b) CW and (c-d) Ni/CW.

**
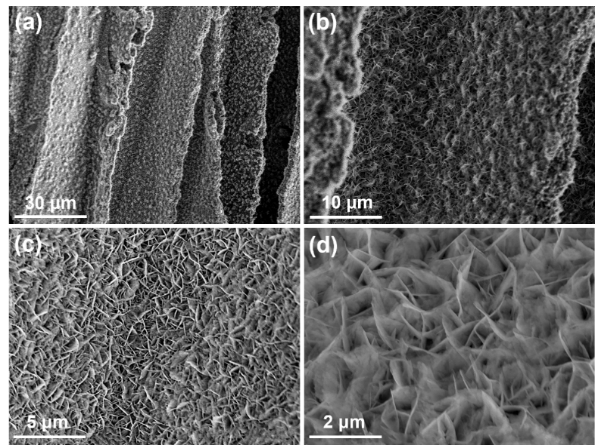
**

**Figure S8.** SEM images of the Precursor.


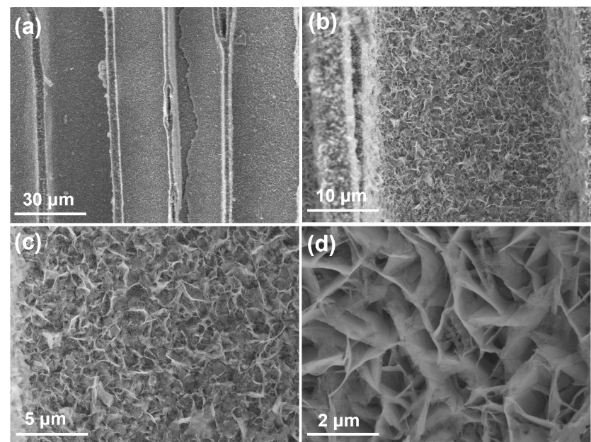


**Figure S9.** SEM images of Ni_3_Fe/MoO_2_/CW.

**
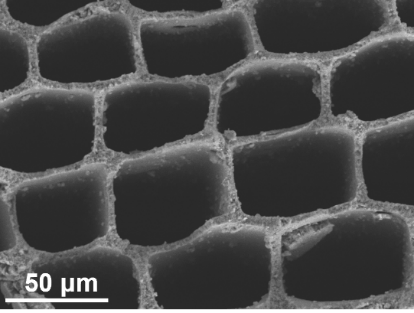
**

**Figure S10.** SEM image of Ni_3_Fe/MoO_2_/CW, cross section.


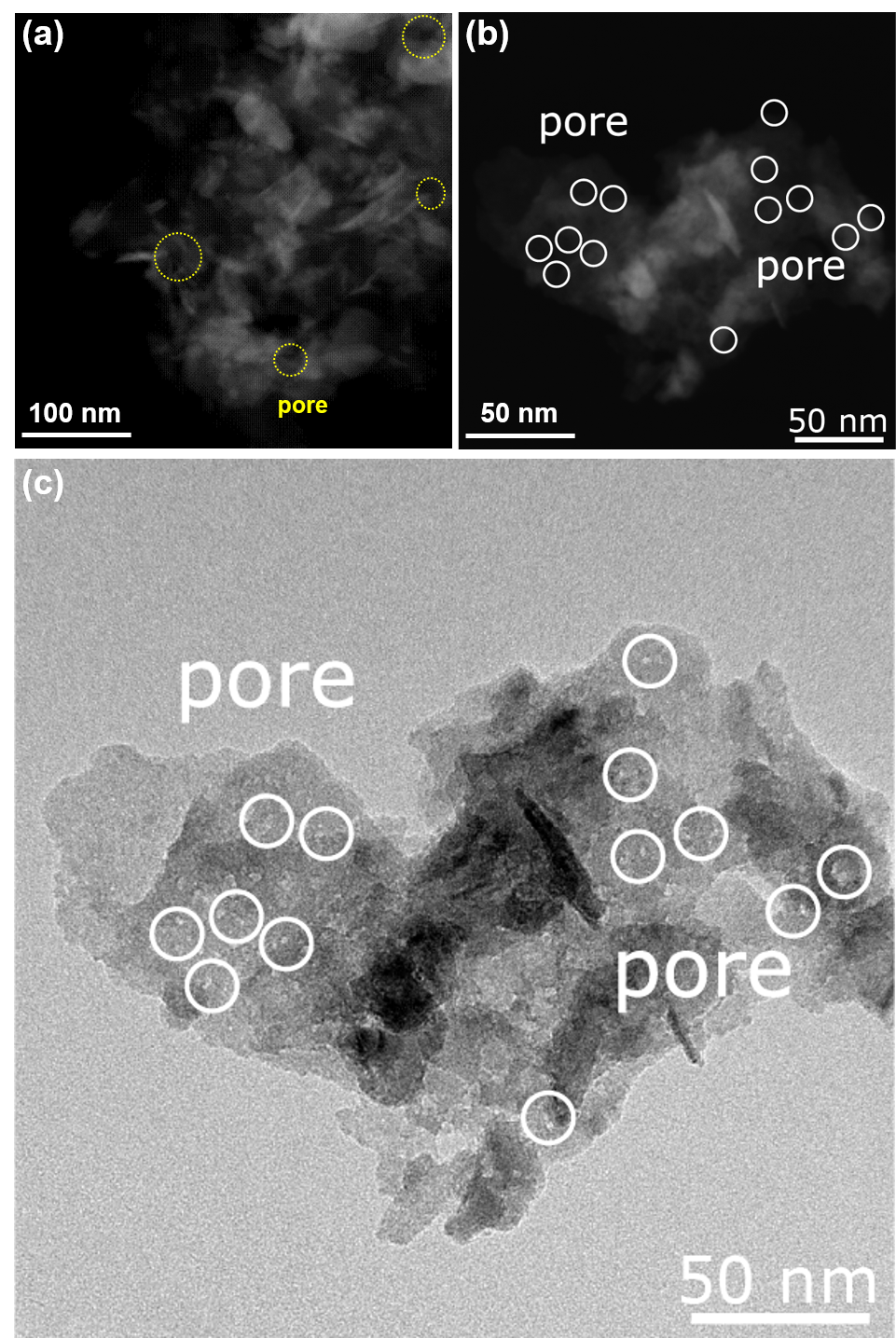


**Figure S11.** HAADF-STEM image of Ni_3_Fe/MoO_2_/CW.


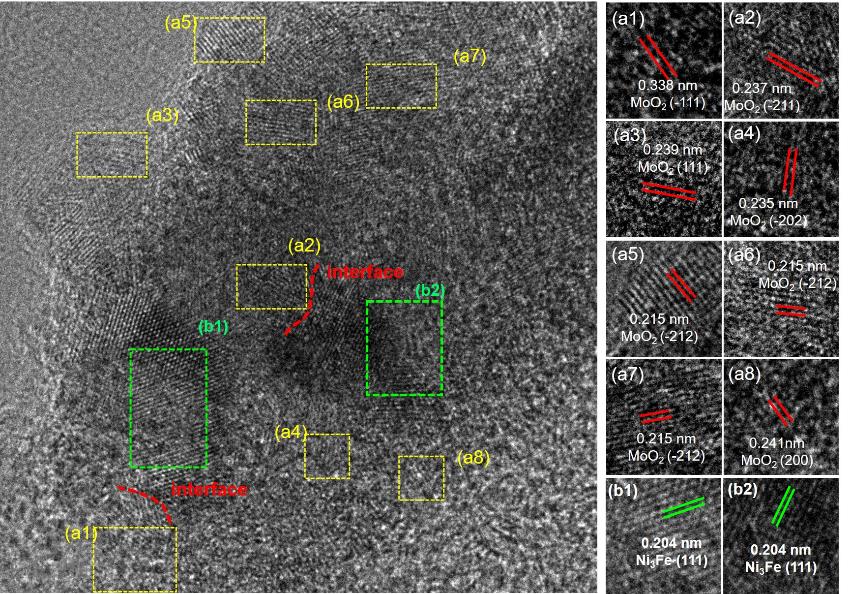


**Figure S12.** HRTEM images of Ni_3_Fe/MoO_2_/CW.


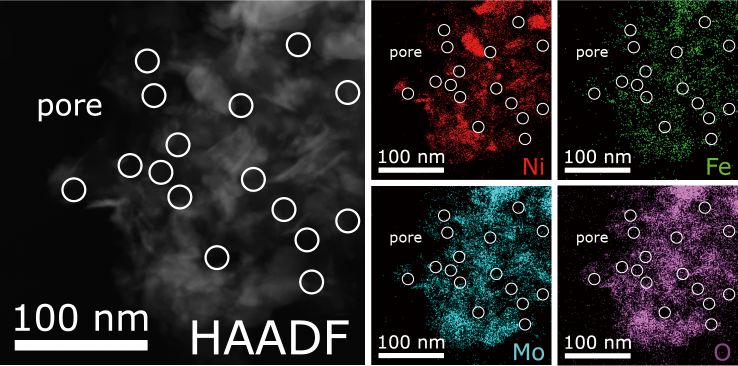


**Figure S13.** HAADF and EDS images of Ni_3_Fe/MoO_2_/CW.


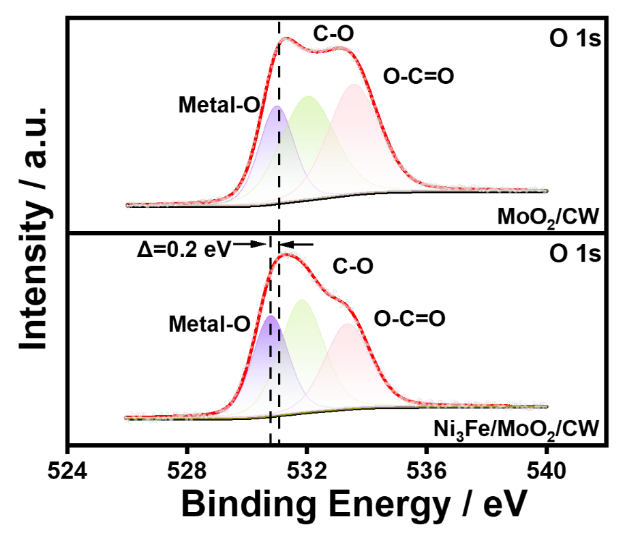


**Figure S14.** XPS spectra of O 1s for Ni_3_Fe/MoO_2_/CW.


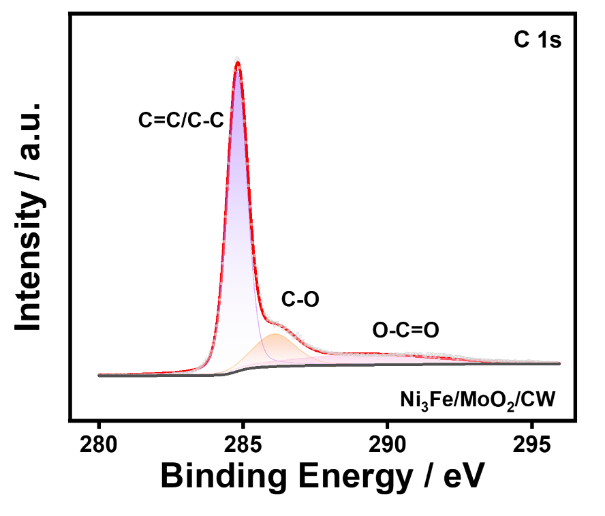


**Figure S15.** XPS spectra of C 1s for Ni_3_Fe/MoO_2_/CW.


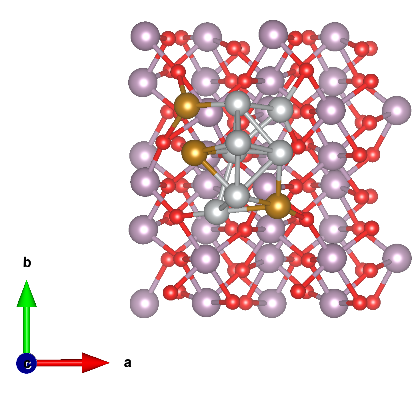


**Figure S16.** The geometry of Ni_3_Fe/MoO_2_/CW.


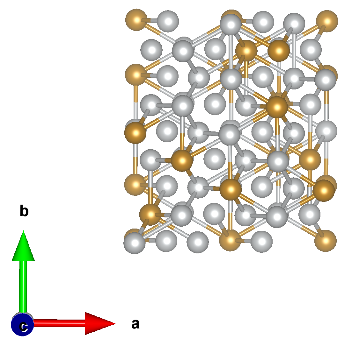


**Figure S17.** The geometry of Ni_3_Fe/CW.


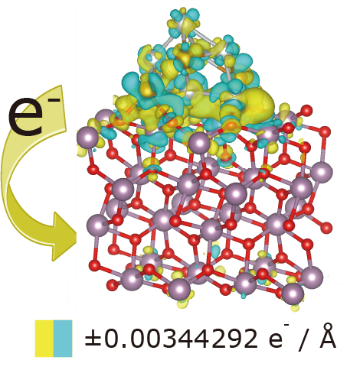


**Figure S18.** Charge density difference map of Ni_3_Fe/MoO_2_/CW.

**Figure S19.** The TDOS of catalysts.

**Figure S20.** DOS of the *s*, *p*, and *d* orbitals at different sites in Ni_3_Fe/MoO_2_/CW.

**
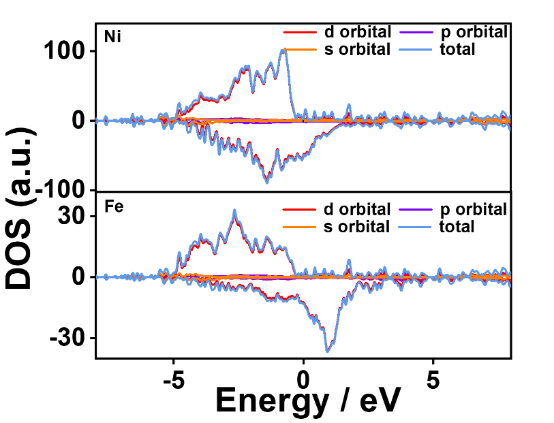
**

**Figure S21.** DOS of the *s*, *p*, and *d* orbitals at different sites in Ni_3_Fe/CW.


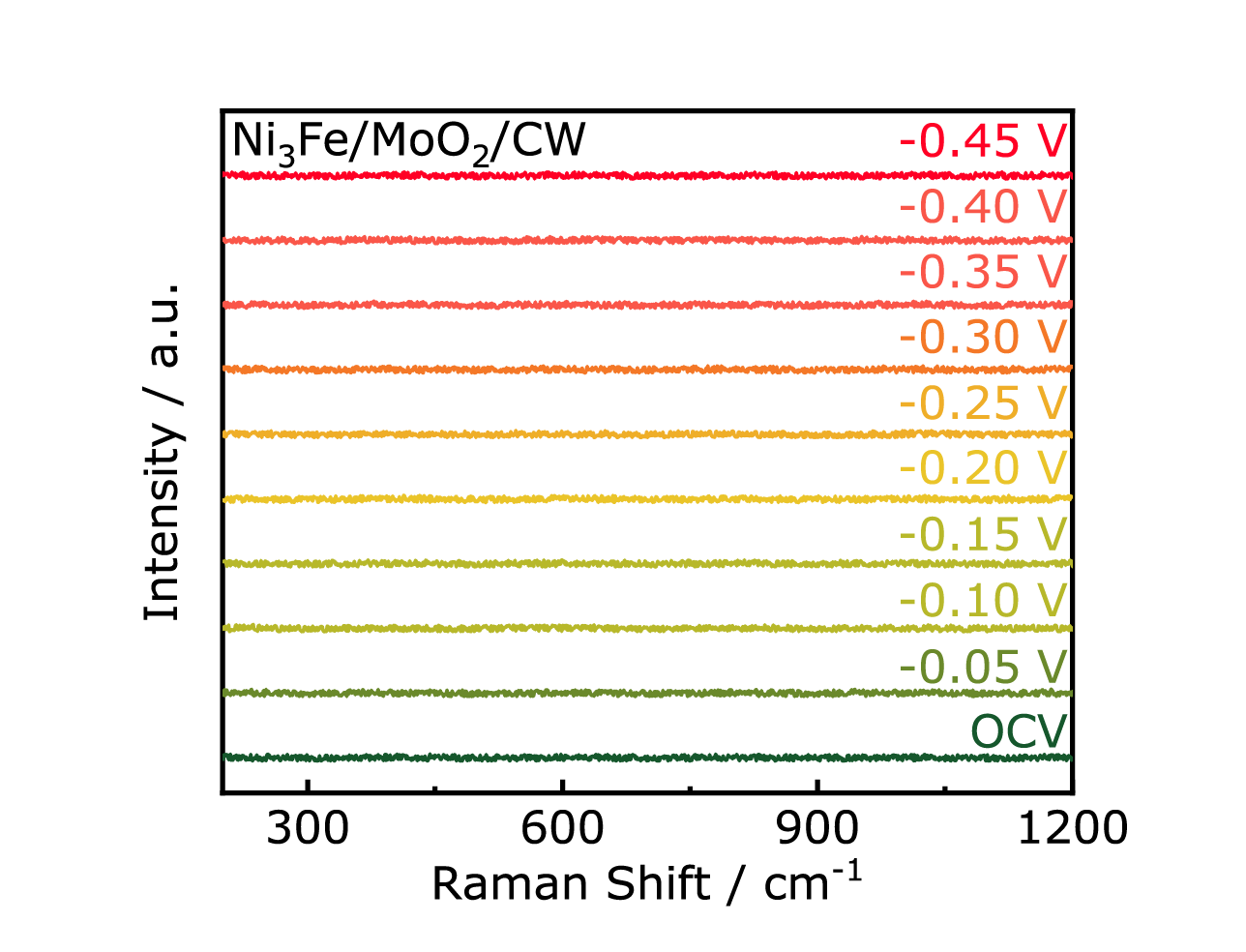


**Figure S22.** In-suit Raman spectrum during HER of Ni_3_Fe/MoO_2_/CW.


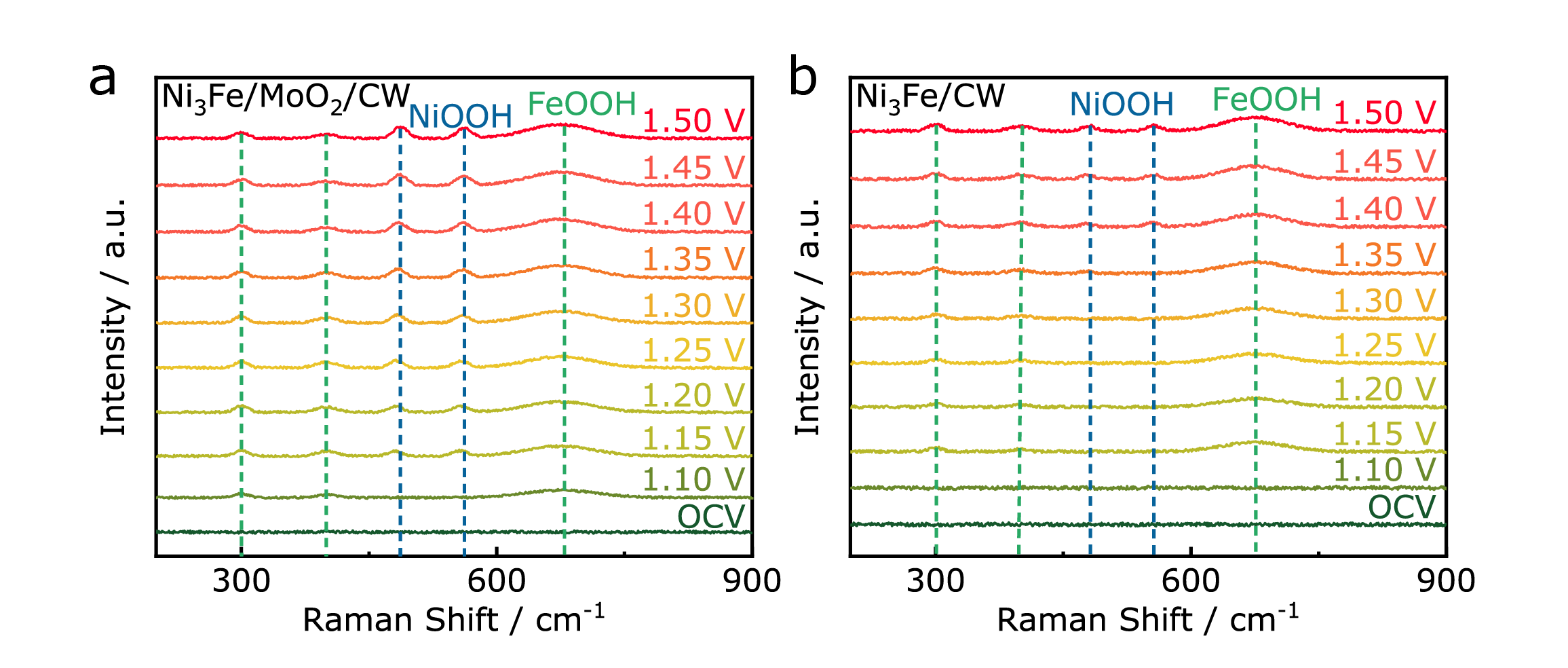


**Figure S23.** In-suit Raman spectrum during OER of (a) Ni_3_Fe/MoO_2_/CW and (b) Ni_3_Fe/CW.


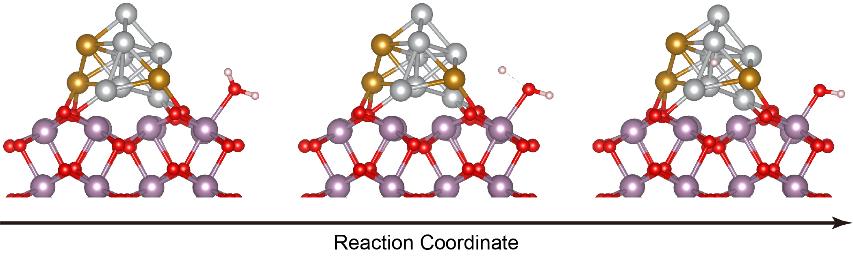


**Figure S24.** The water dissociation process of Ni_3_Fe/MoO_2_/CW.


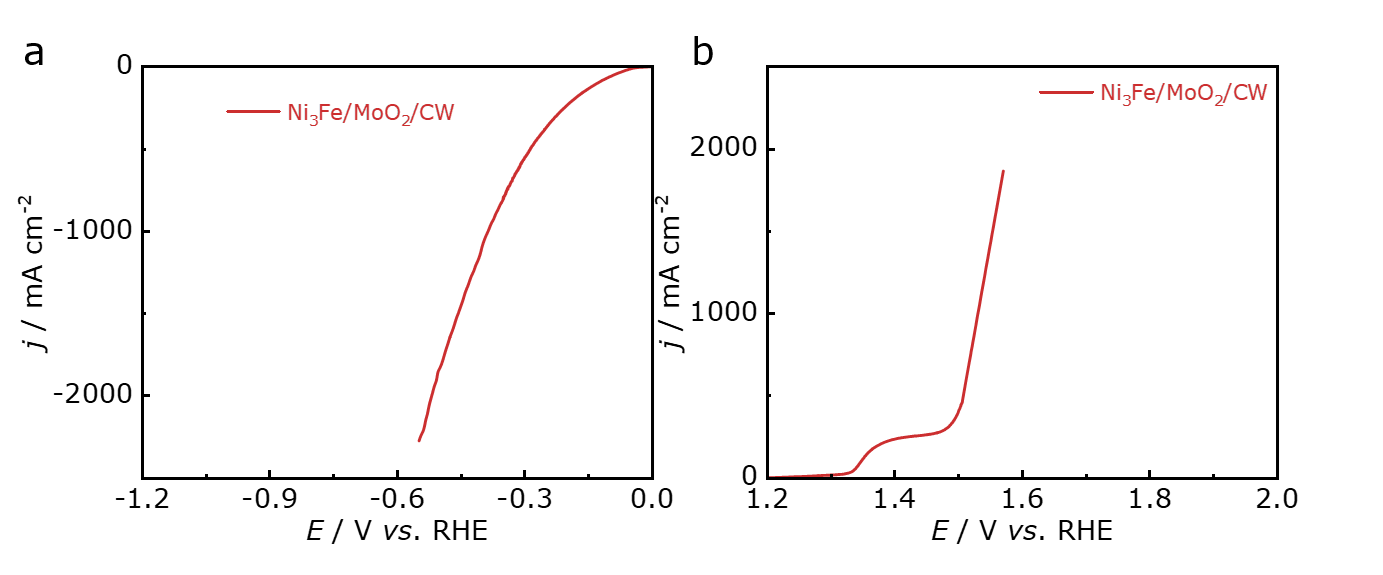


**Figure S25.** Large-scale LSV curves for (a) HER and (b) OER of Ni_3_Fe/MoO_2_/CW.

**Effect of Calcination Temperature on HER/OER Performance of Ni_3_Fe/MoO_2_/CW**

The annealing temperature plays a pivotal role in modulating the morphology and electrocatalytic performance of Ni_3_Fe/MoO_2_/CW. Scanning electron microscopy (SEM) images reveal that at lower temperatures (350 °C), the insufficient dehydration results in poorly developed porosity and limited nanoparticle formation, thereby impeding gas-liquid transport during electrolysis (**Figure S26**). Furthermore, XRD analysis shows that the diffraction peaks corresponding to Ni_3_Fe and MoO_2_ are barely detectable at this temperature (**Figure S27**), indicating inadequate metal reduction and failure to form a well-defined heterostructure, factors that negatively impact catalytic performance. In contrast, although high-temperature annealing at 550 °C successfully induces heterojunction formation, it also leads to structural collapse of the nanosheets and pronounced aggregation of nanoparticles, owing to rapid dehydration (**Figures S28-S29**). This structural deterioration significantly reduces the number of accessible active sites. Electrochemical evaluations (**Figures S30-S31**) demonstrate that Ni_3_Fe/MoO_2_/CW annealed at 450 °C exhibits the most favorable HER and OER activities. This can be attributed to the development of a well-defined porous nanosheet architecture that optimizes active site exposure and enhances both mass and gas transport. Moreover, the catalyst maintains excellent catalytic performance across a wide range of current densities, underscoring its strong potential for application in industrial-scale water electrolysis^[1,2]^.


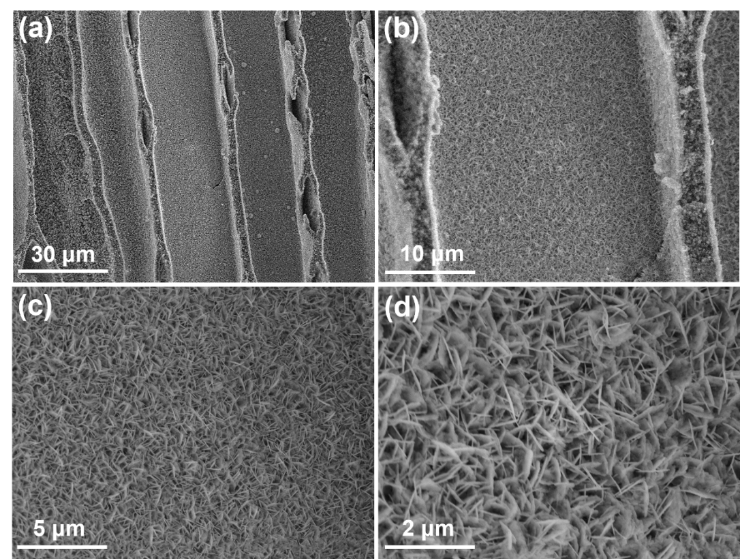


**Figure S26.** SEM images of Ni_3_Fe/MoO_2_/CW after annealing at 350 °C.


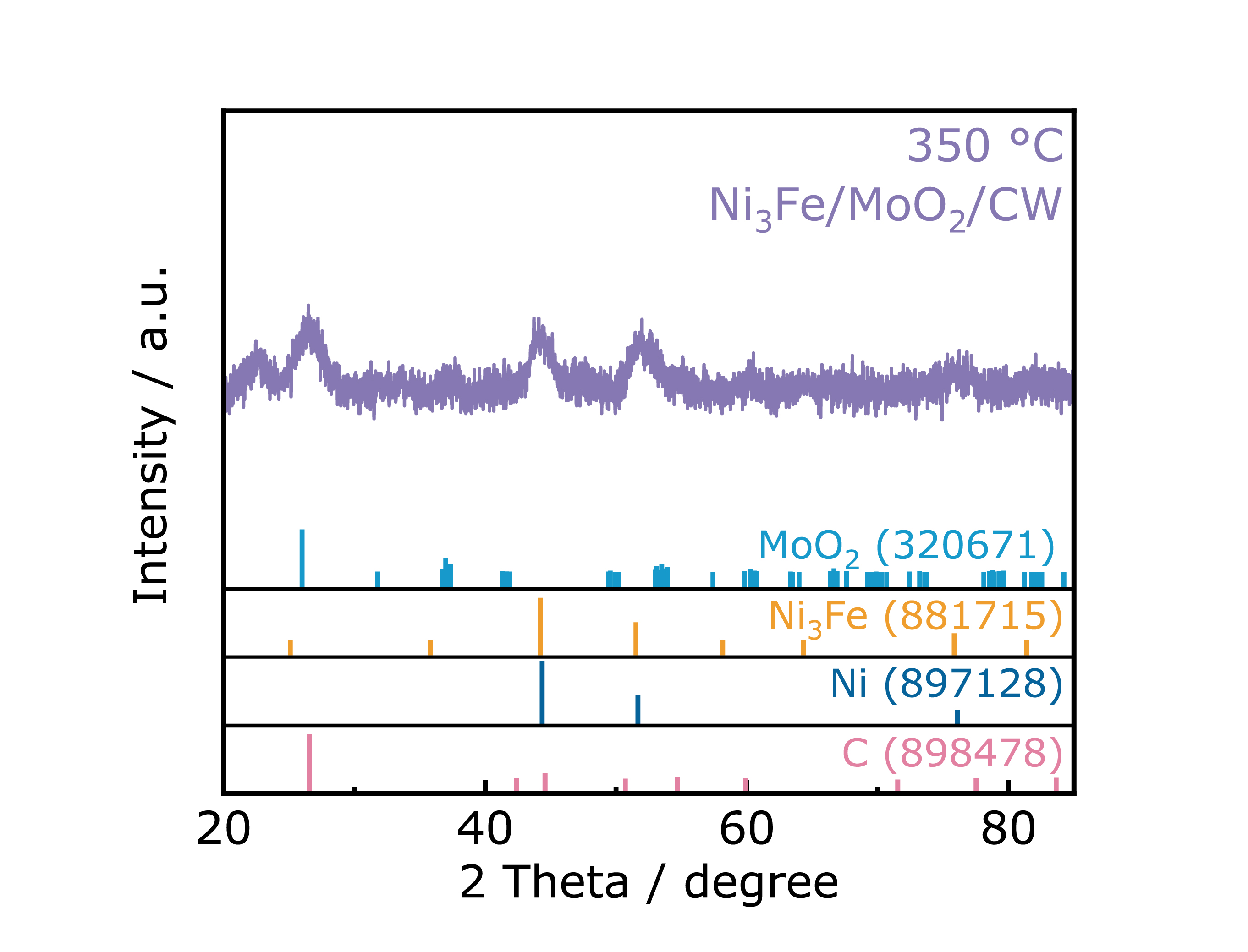


**Figure S27.** XRD pattern of Ni_3_Fe/MoO_2_/CW after annealing at 350 °C.


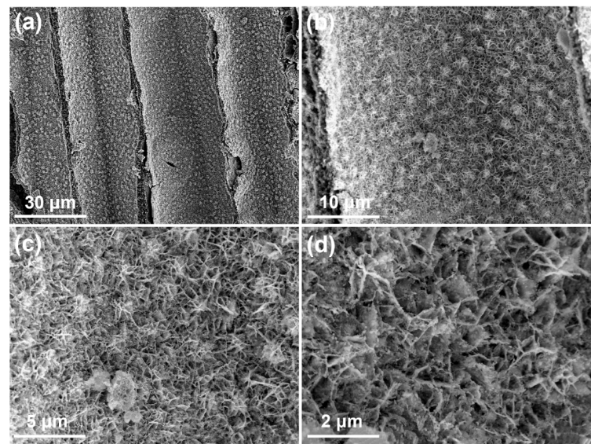


**Figure S28.** SEM images of Ni_3_Fe/MoO_2_/CW after annealing at 550 °C.


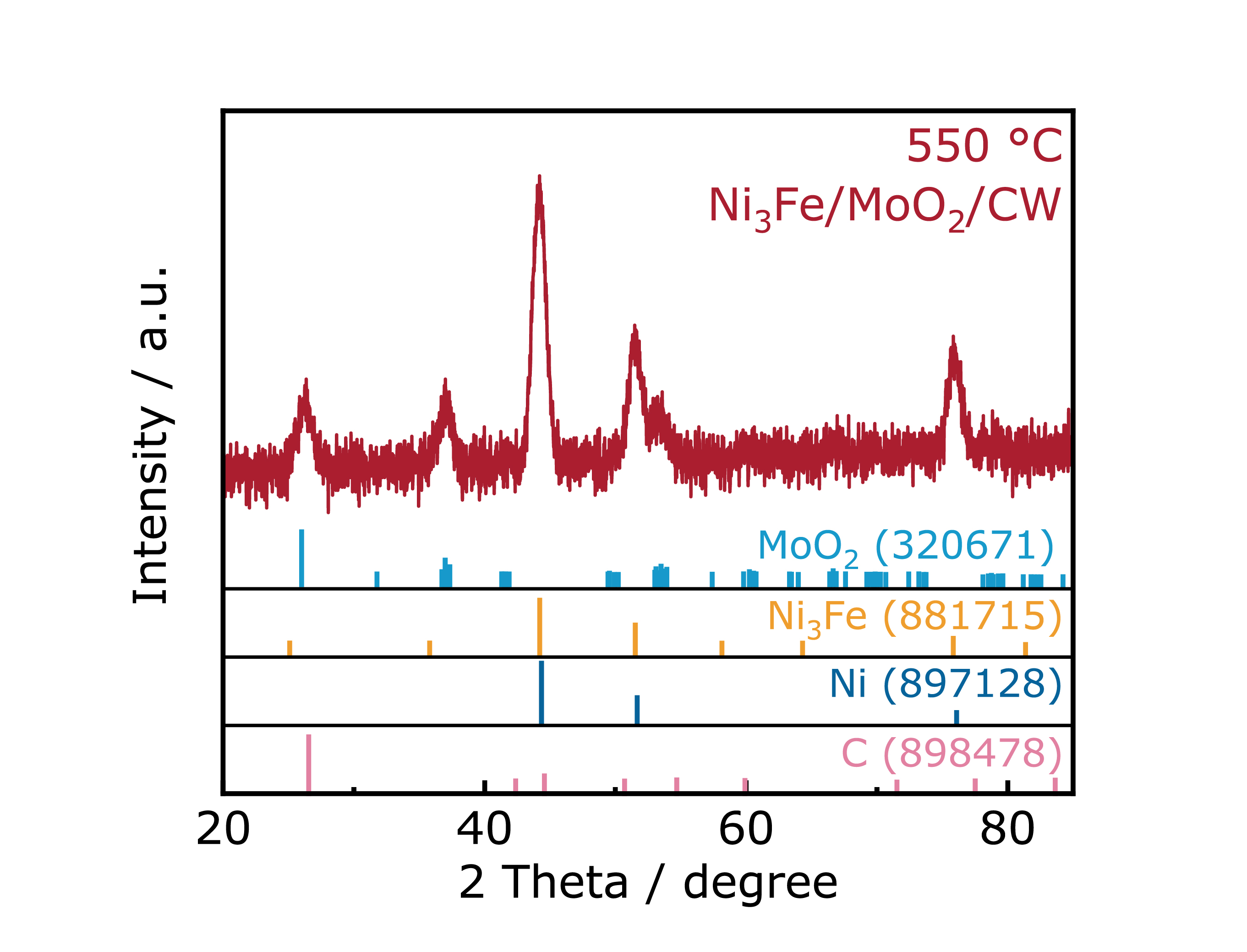


**Figure S29.** XRD pattern of Ni_3_Fe/MoO_2_/CW after annealing at 550 °C.


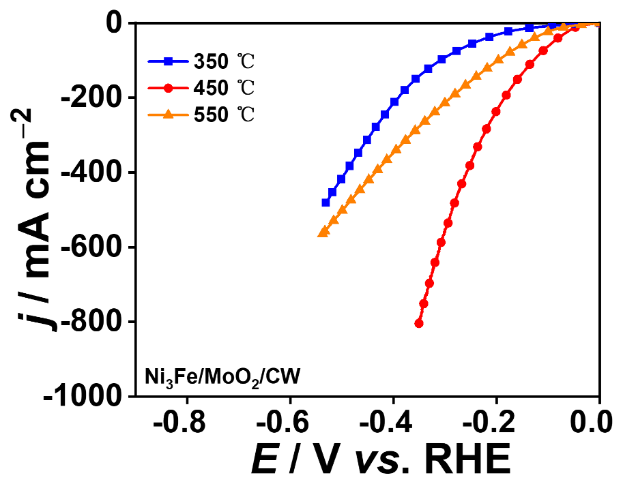


**Figure S30.** LSV curves of the Ni_3_Fe/MoO_2_/CW under different annealing temperatures for HER.

**Figure S31.** LSV curves of the Ni_3_Fe/MoO_2_/CW under different annealing temperatures for OER.


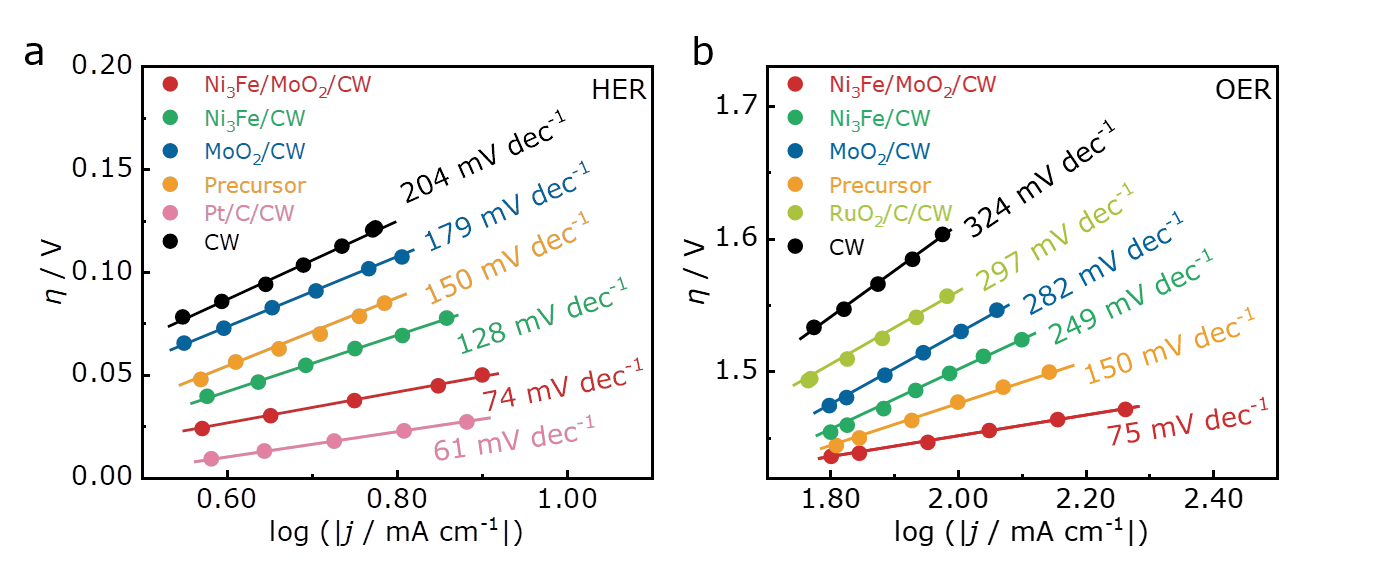


**Figure S32.** Tafel slope of all catalysts for (a) HER and (b) OER.


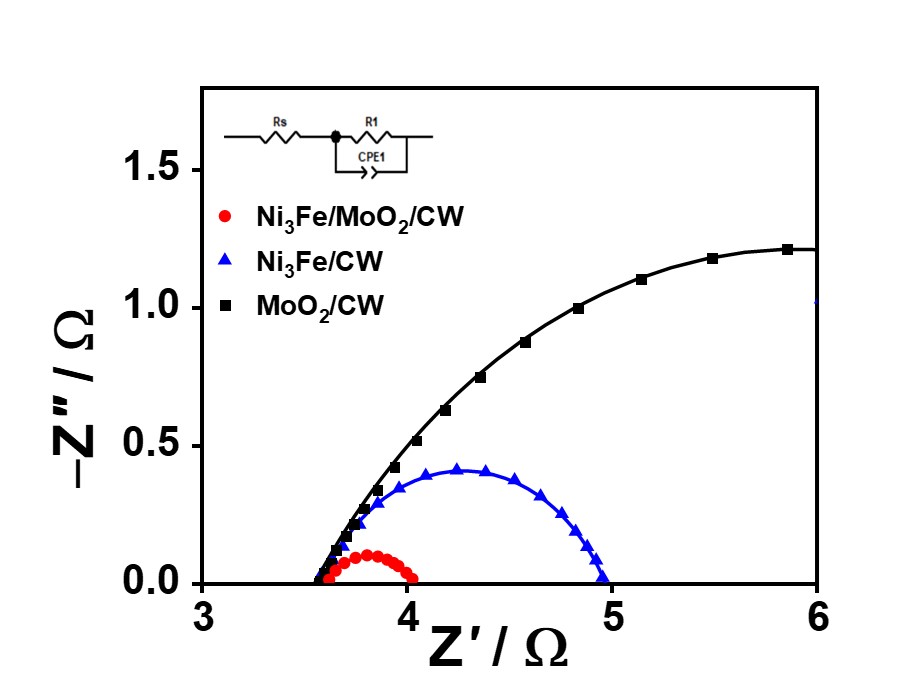


**Figure S33.** Nyquist plots of Ni_3_Fe/MoO_2_/CW, Ni_3_Fe/CW, and MoO_2_/CW for HER.


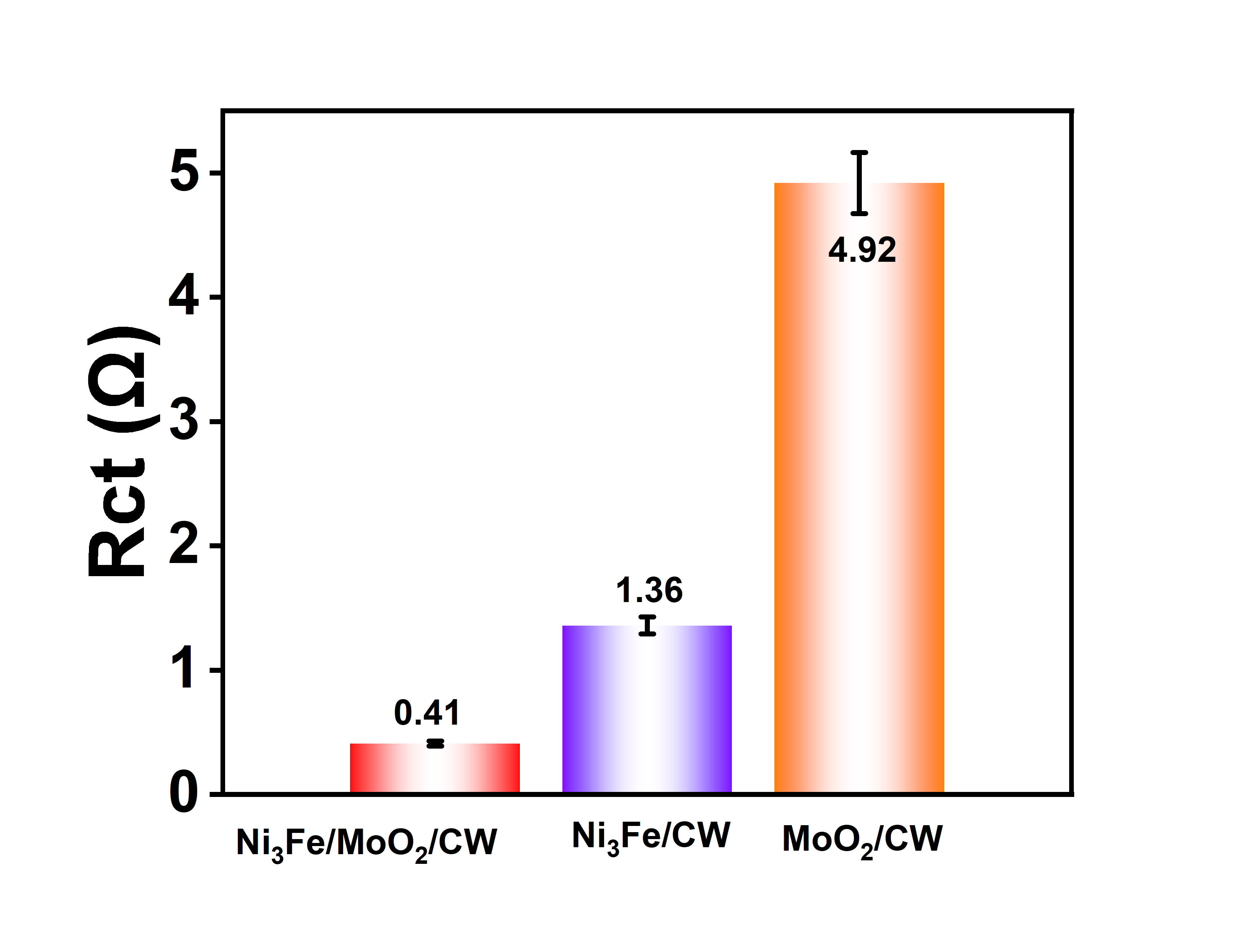


**Figure S34.** R_ct_ of Ni_3_Fe/MoO_2_/CW, Ni_3_Fe/CW, and MoO_2_/CW for HER.


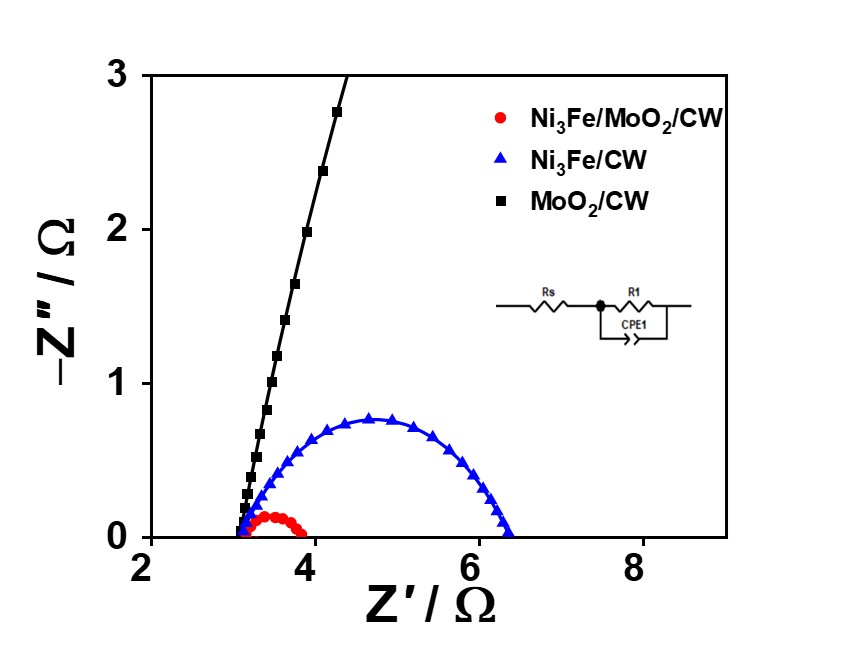


**Figure S35.** Nyquist plots of Ni_3_Fe/MoO_2_/CW, Ni_3_Fe/CW, and MoO_2_/CW for OER.


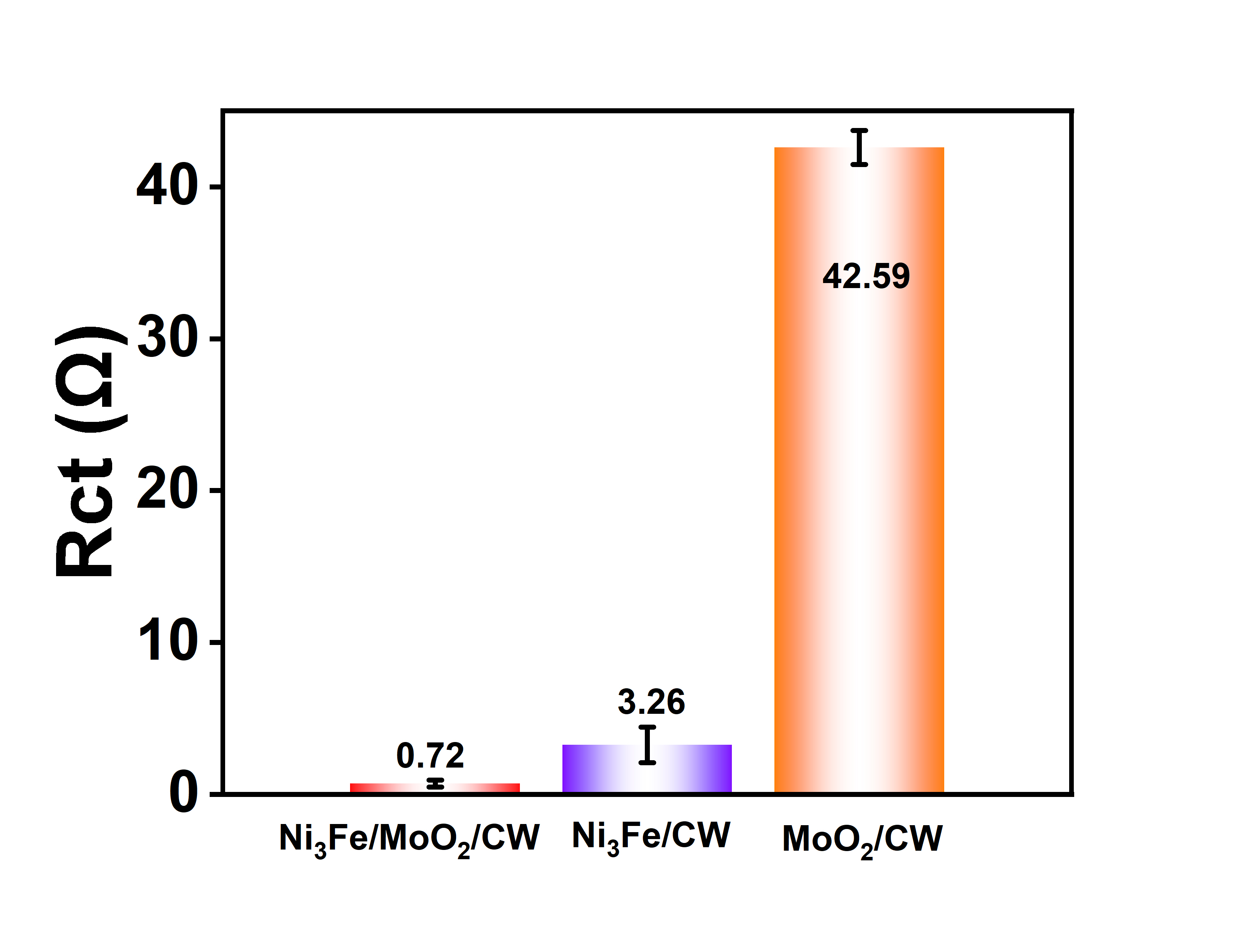


**Figure S36.** R_ct_ of Ni_3_Fe/MoO_2_/CW, Ni_3_Fe/CW, and MoO_2_/CW for OER.


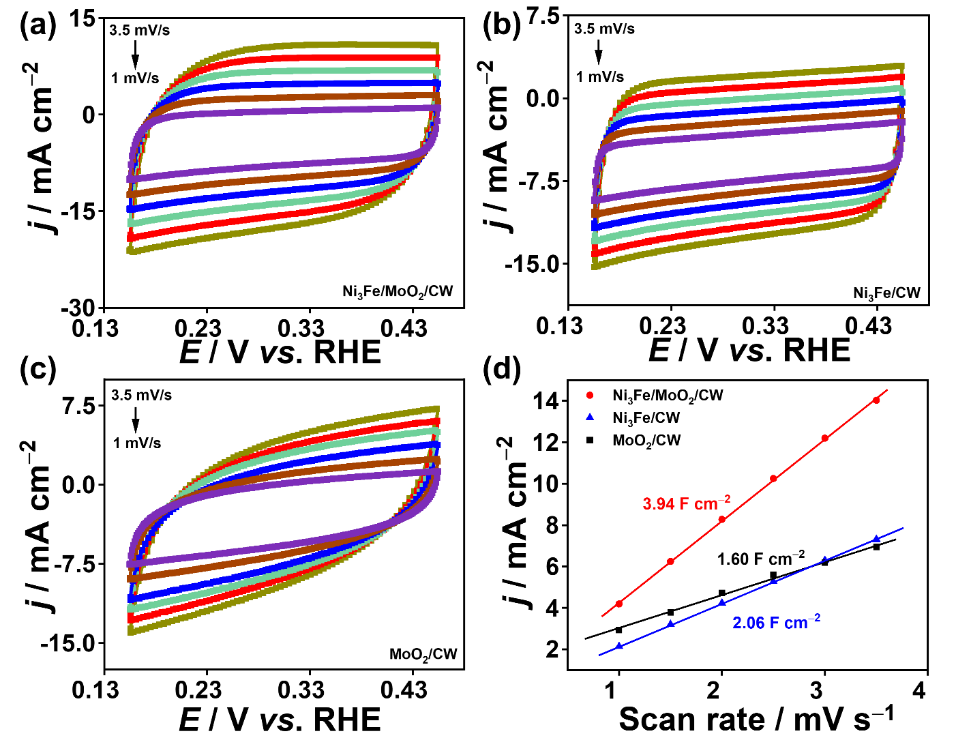


**Figure S37.** (a-c) The CV values of the catalysts with scan rates from 1 to 3.5 mV s^−1^ and scanning potentials ranging from 0.15 to 0.45 V in alkaline solution, and (d) The corresponding double layer capacitance.


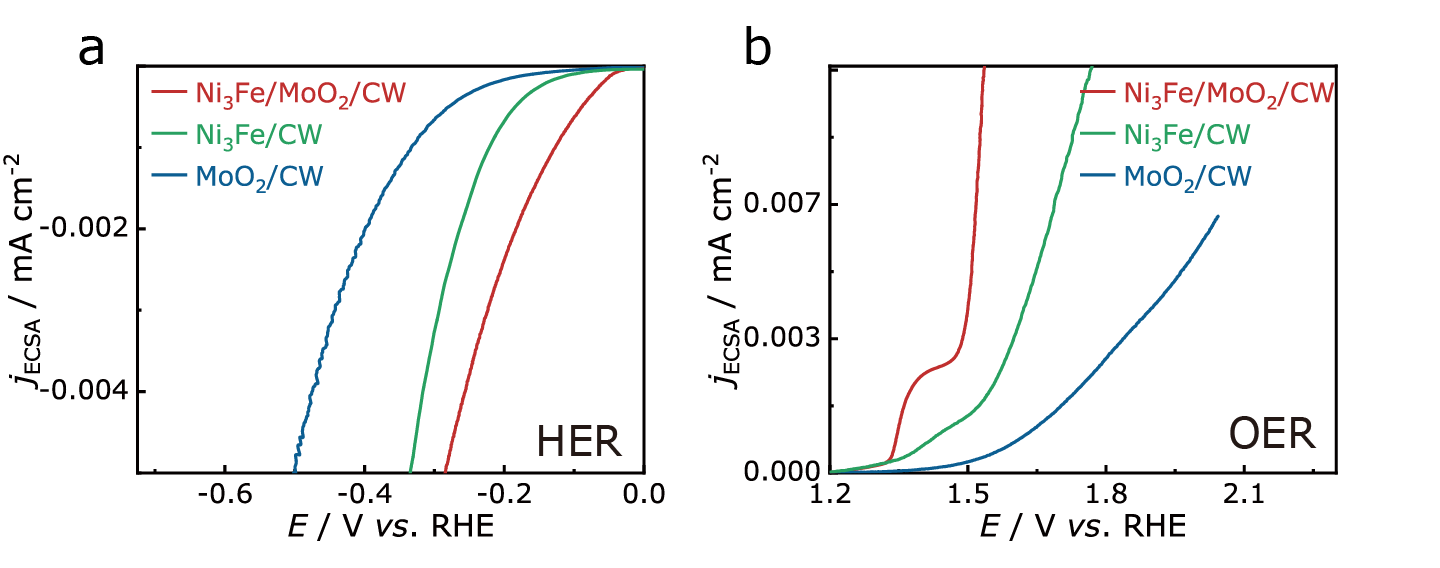


**Figure S38.** ECSA correction of all catalysts for (a) HER and (b) OER.


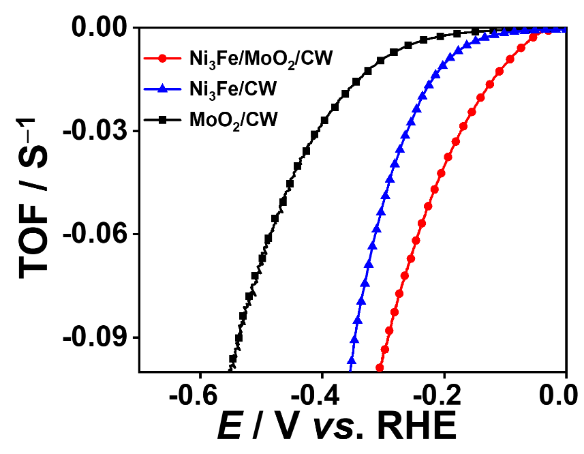


**Figure S39.** TOF of HER for catalysts.


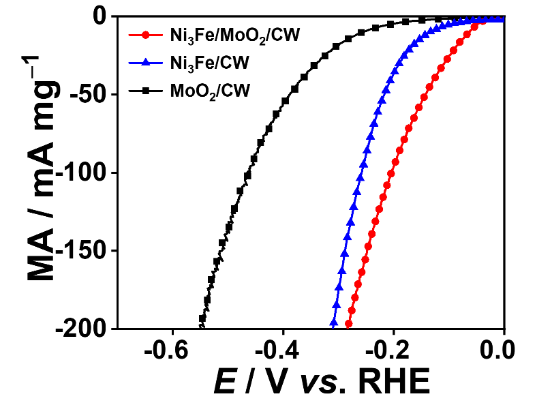


**Figure S40.** MA of HER for catalysts.


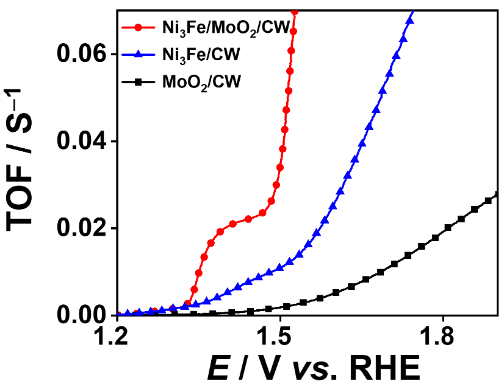


**Figure S41.** TOF of OER for catalysts.


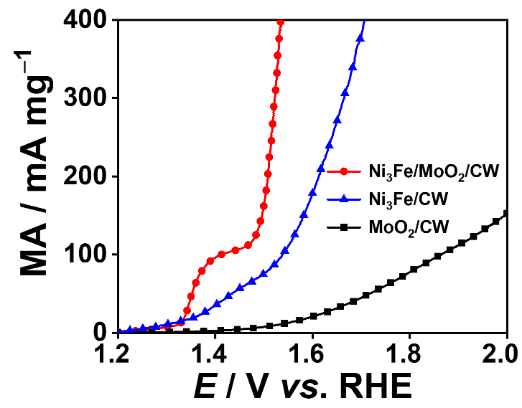


**Figure S42.** MA of OER for catalysts.


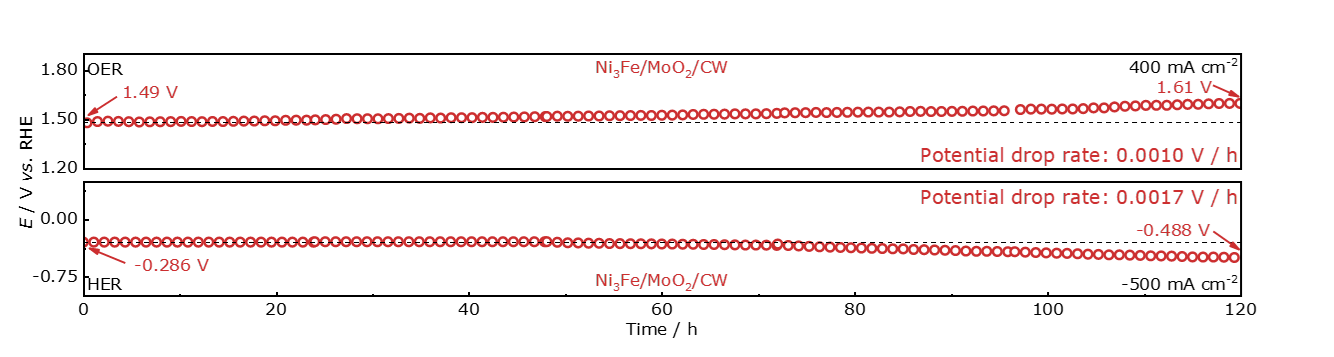


**Figure S43.** Stability test for Ni_3_Fe/MoO_2_ in HER and OER.

**Figure S44.** LSV curves before and after the HER stability test for Ni_3_Fe/MoO_2_/CW.

**Figure S45.** LSV curves before and after the OER stability test for Ni_3_Fe/MoO_2_/CW.


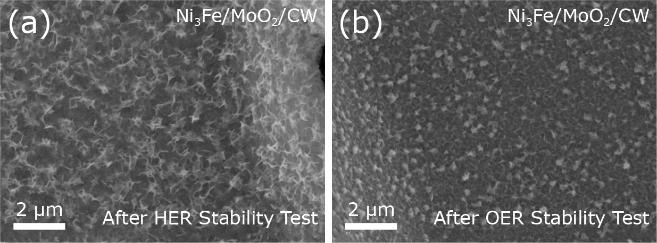


**Figure S46.** SEM after HER/OER stability test.


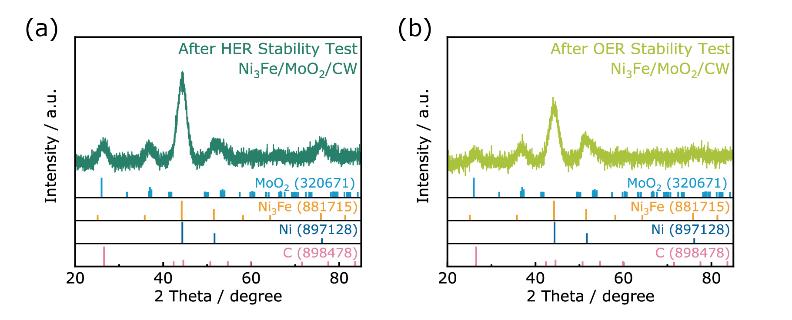


**Figure S47.** XRD after HER/OER stability test.


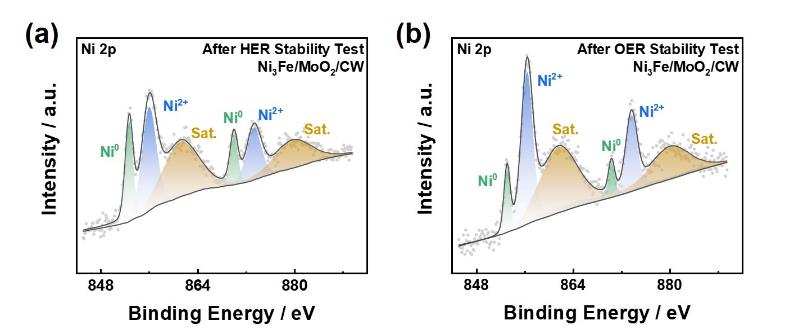


**Figure S48.** Ni 2p XPS spectra of Ni_3_Fe/MoO_2_ after HER/OER stability test.


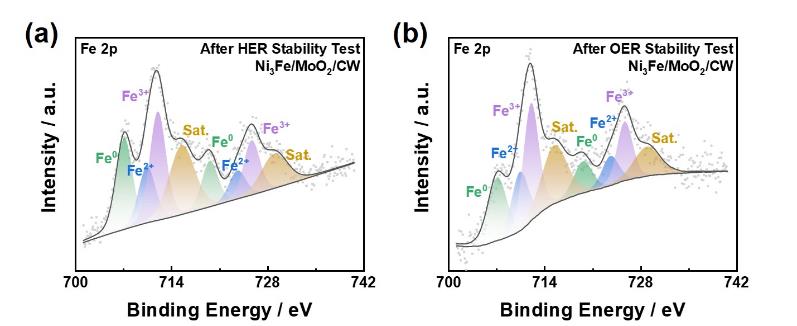


**Figure S49.** Fe 2p XPS spectra of Ni_3_Fe/MoO_2_ after HER/OER stability test.


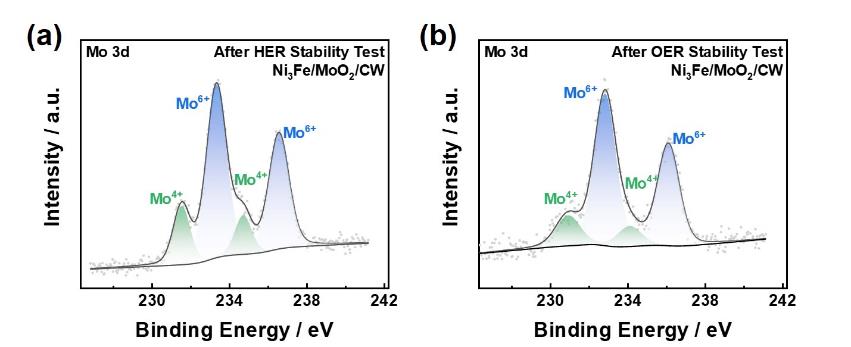


**Figure S50.** Mo 3d XPS spectra of Ni_3_Fe/MoO_2_ after HER/OER stability test.


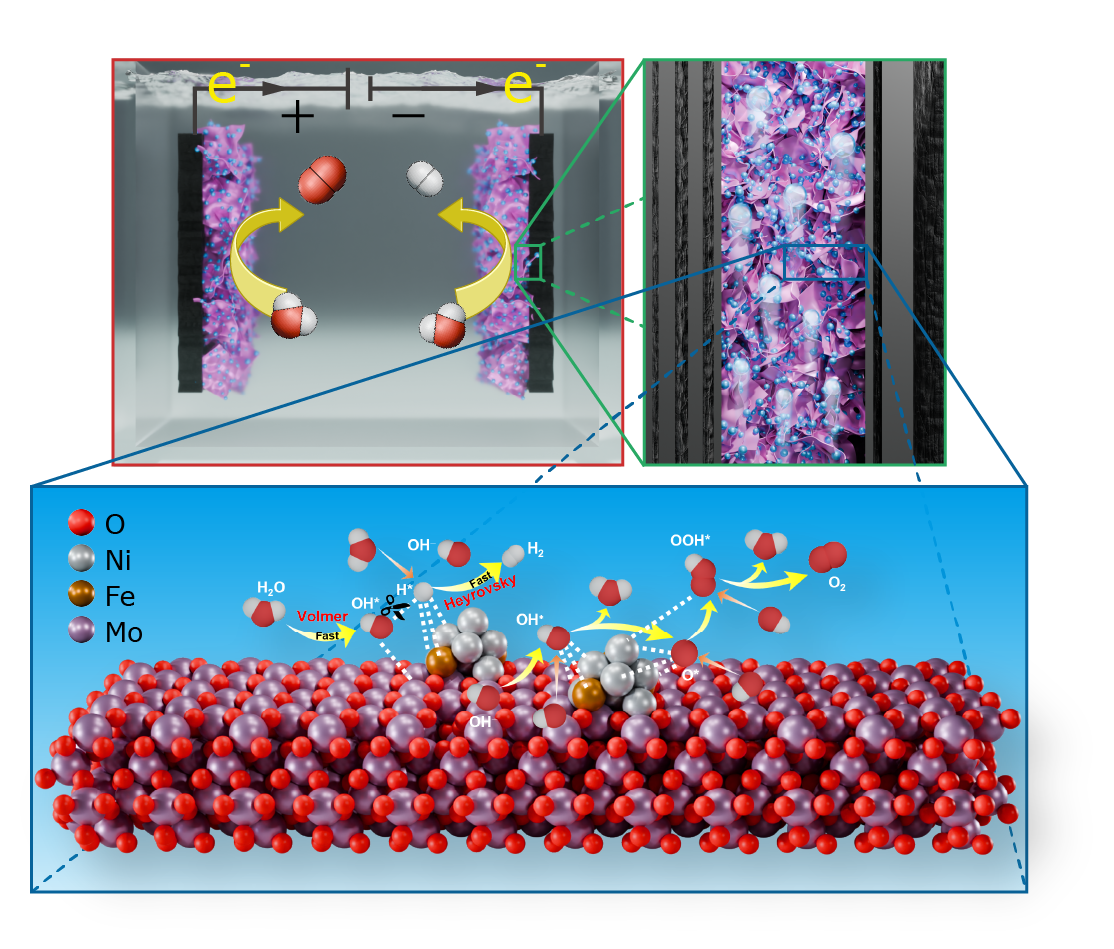


**Figure S51.** Schematic of water electrolysis.

.


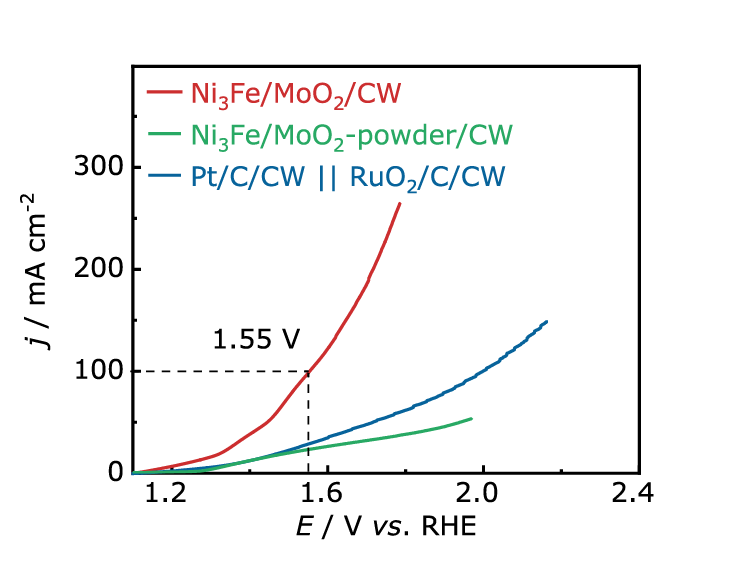


**Figure S52.** LSV curves of water electrolysis.


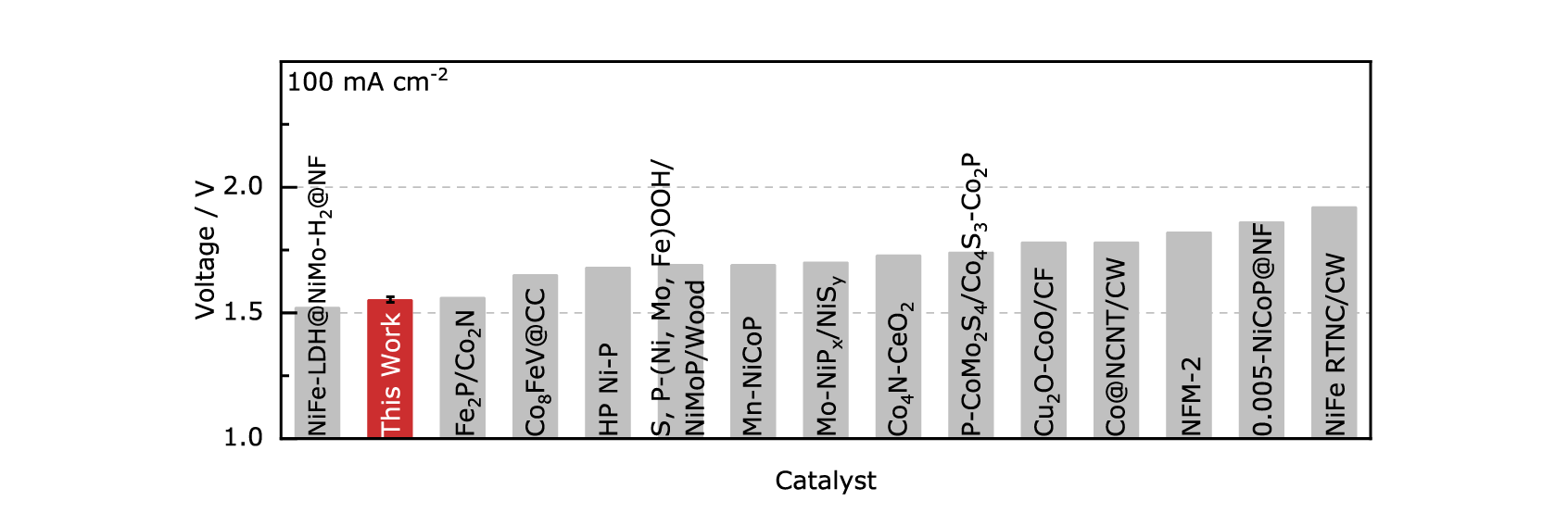


**Figure S53.** Comparison with reported water electrolysis catalyst.


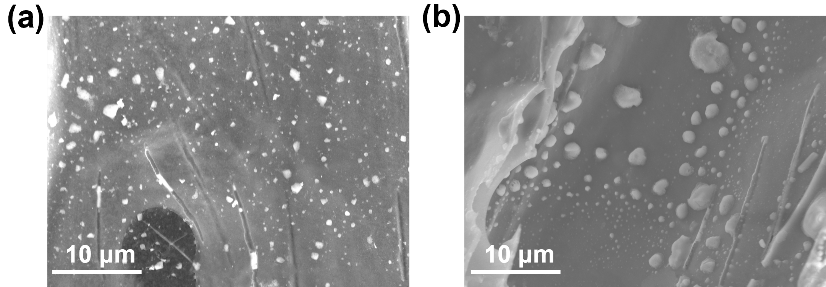


**Figure S54.** SEM images of Ni_3_Fe/MoO_2_-powder/CW (a) before and (b) after water electrolysis.


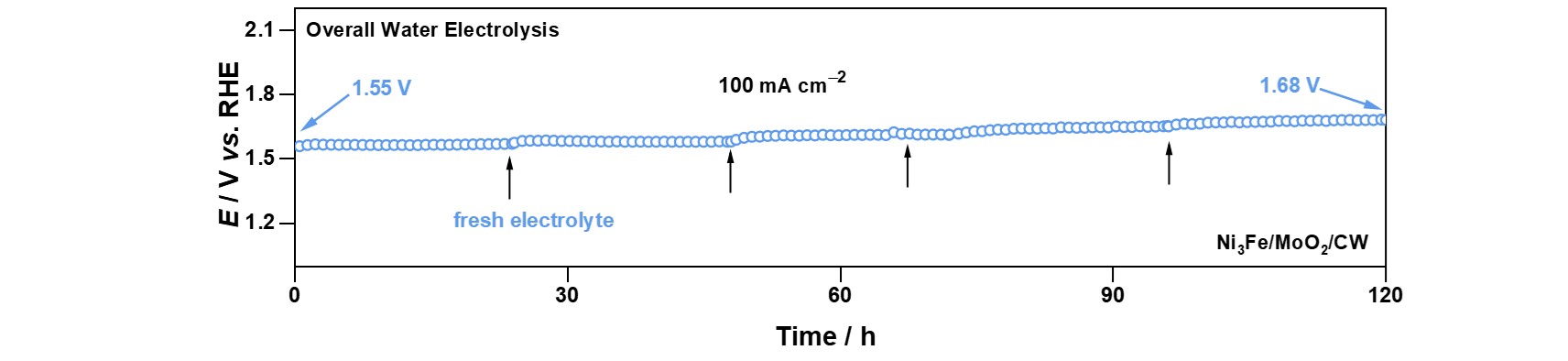


**Figure S55.** The stability of Ni_3_Fe/MoO_2_/CW’s water electrolysis.


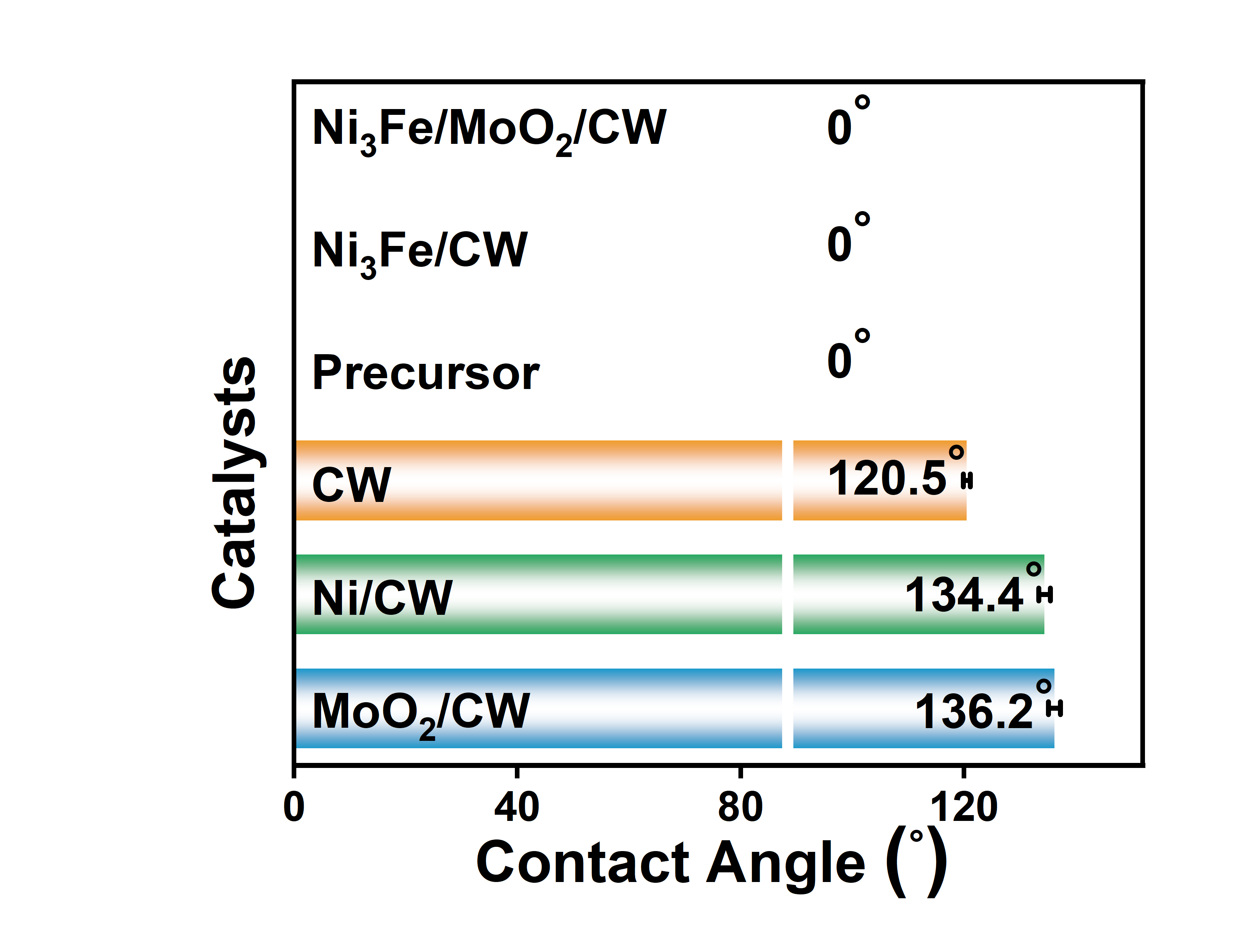


**Figure S56.** Contact angle of catalysts.


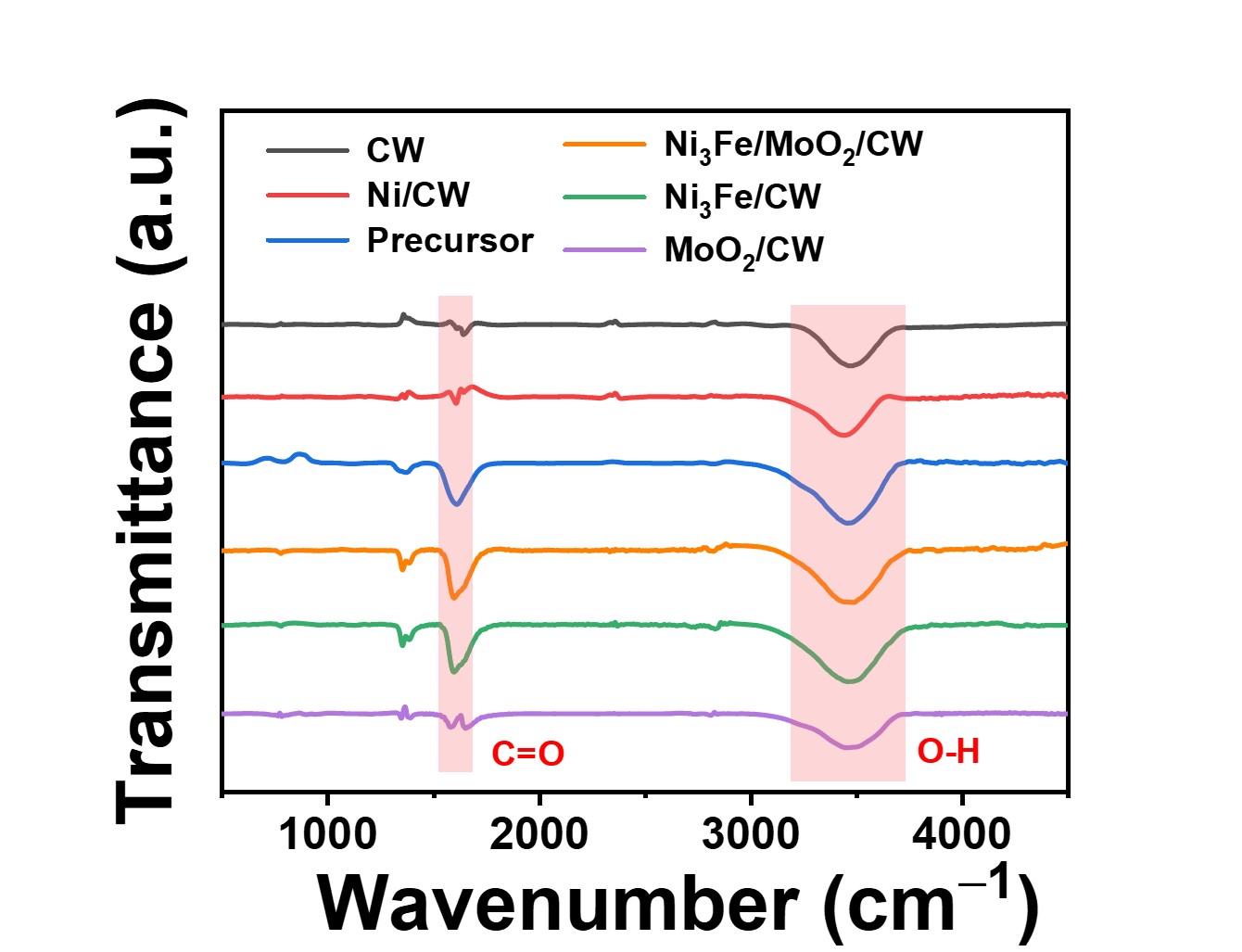


**Figure S57.** The Fourier transform infrared spectroscopy of catalysts.


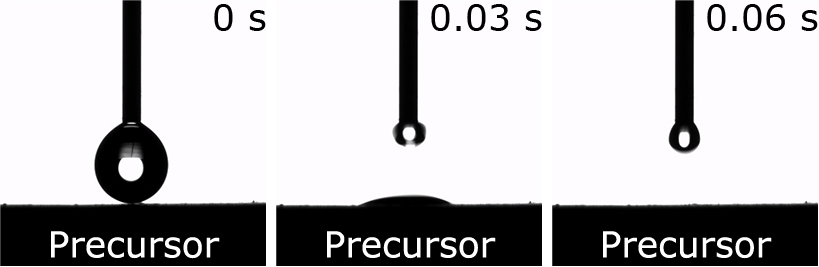


**Figure S58.** Contact angle of Precursor.


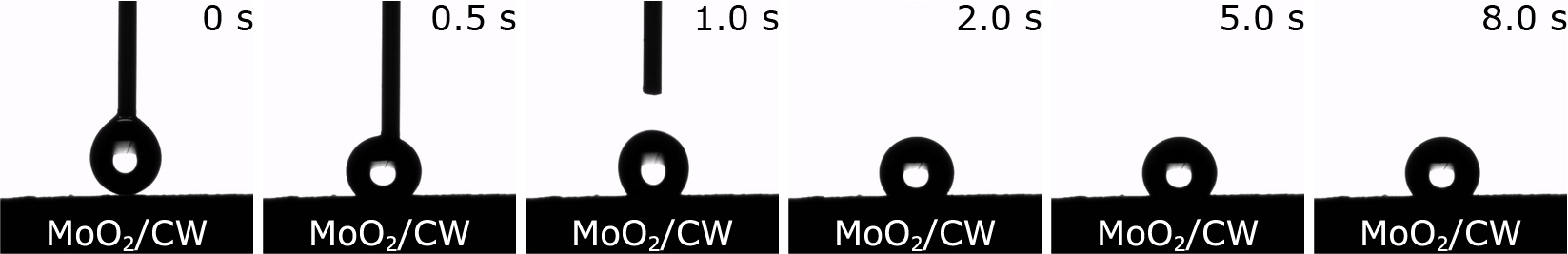


**Figure S59.** Contact angle of MoO_2_/CW.

**
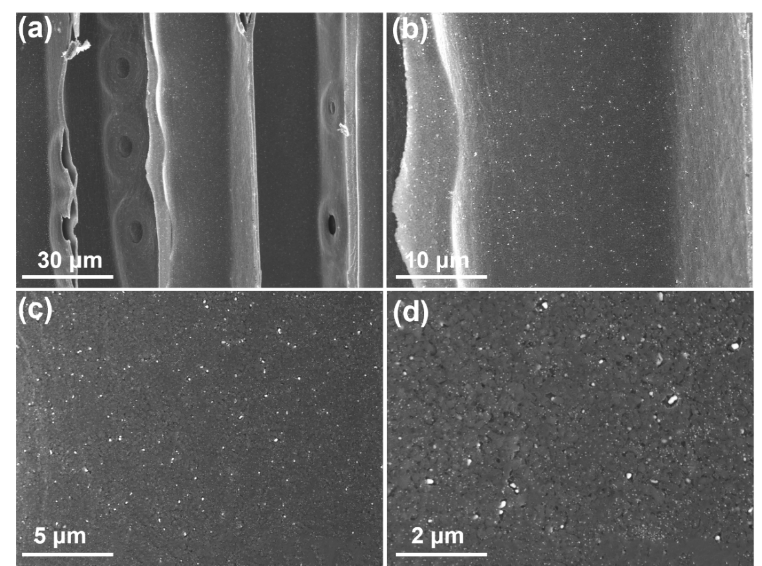
**

**Figure S60.** SEM images of Ni_3_Fe/CW.


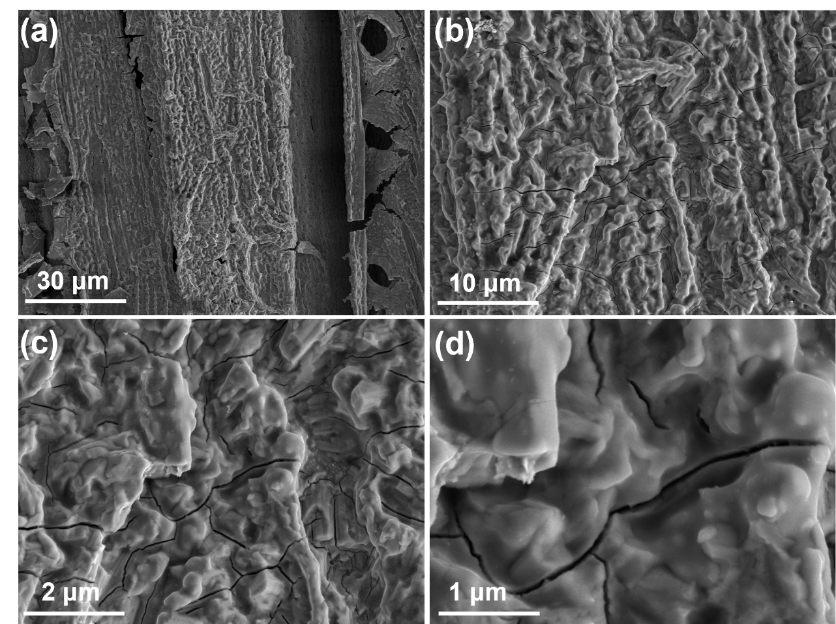


**Figure S61.** SEM images of MoO_2_/CW.

**Effect of Calcination Temperature on Specific Surface Area of Ni_3_Fe/MoO_2_/CW**

The influence of calcination temperature on the specific surface area of Ni_3_Fe/MoO_2_ was systematically evaluated and found to correlate closely with morphological changes observed in SEM images. At a lower temperature (350 °C), insufficient dehydration resulted in a poorly developed porous nanosheet structure, limiting the overall surface area and the exposure of active catalytic sites (**Figure S26** and **Figure S63**). Conversely, excessive calcination at 550 °C led to the collapse of the nanosheet architecture and agglomeration of nanoparticles due to rapid dehydration, which also reduced the accessible surface area (**Figure S28** and **Figure S63**). Notably, the sample calcined at 450 °C exhibited the most well-defined porous nanosheet morphology, as evidenced by SEM, and achieved the highest specific surface area (**Table S8**). This optimized structure maximized the availability of active sites and is considered a key factor in the enhanced catalytic performance observed at this temperature^[3]^.


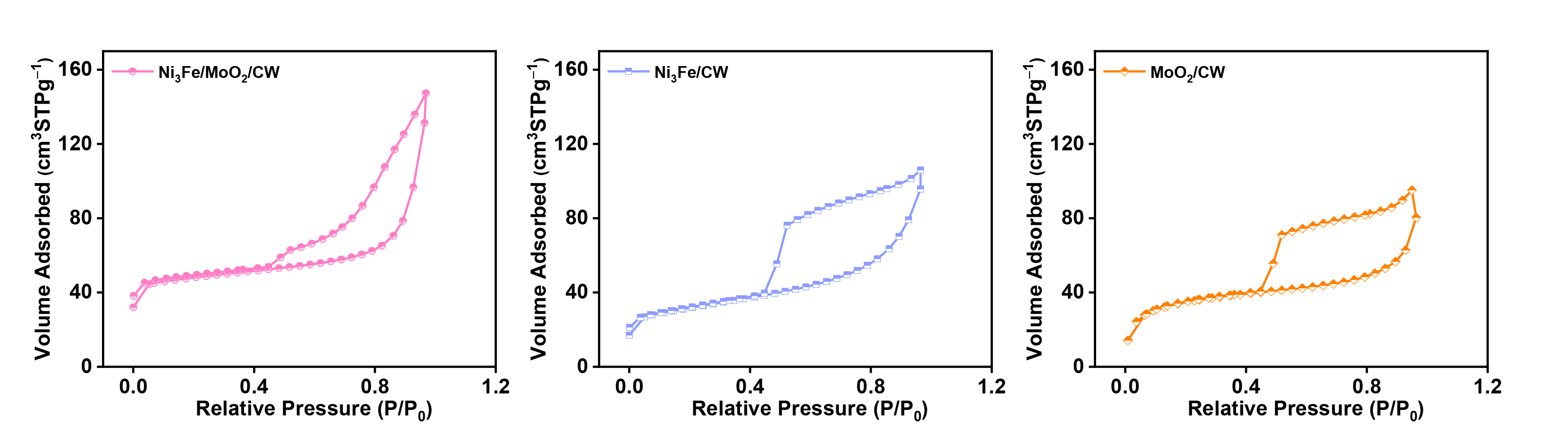


**Figure S62.** N_2_ adsorption and resolution isotherms of all catalysts.


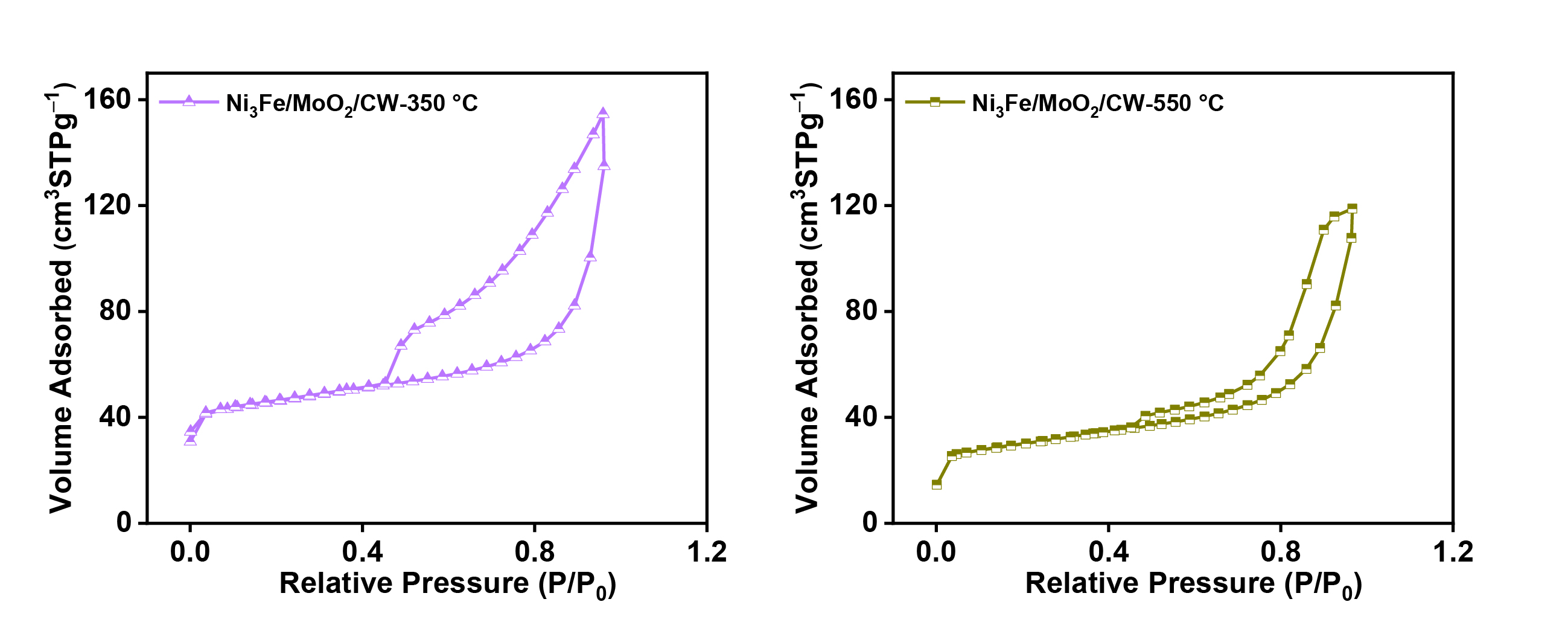


**Figure S63.** N_2_ adsorption and resolution isotherms of Ni_3_Fe/MoO_2_/CW under different calcination temperature.


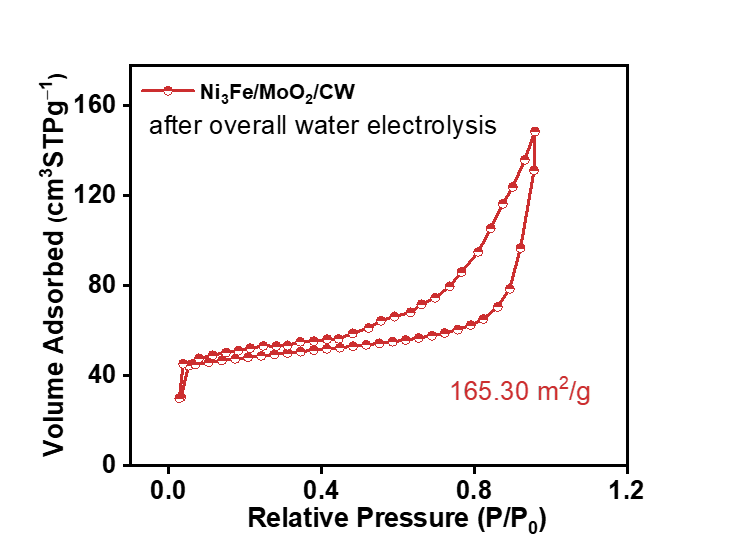


**Figure S64.** N_2_ adsorption and resolution isotherms of Ni_3_Fe/MoO_2_/CW after overall water electrolysis.

1. **Supplementary Tables**

**Table S1.** Elemental composition of the catalysts obtained from ICP-MS.

| **Elements**  **Catalysts** | **Ni** **(wt.%)** | **Fe (wt.%)** | **Mo** **(wt.%)** |
| --- | --- | --- | --- |
| **Ni_3_Fe/MoO_2_/CW** | 20.34 | 6.07 | 73.59 |
| **Ni_3_Fe/CW** | 76.59 | 23.41 | N/A |
| **MoO_2_/CW** | N/A | N/A | 100 |

**Table S2.** EXAFS fitting parameters for various samples

| **Sample** | **Shell** | **CN*^a^*** | **R*^b^* (Å)** | **σ^2^*^c^* (Å^2^)** | **ΔE_0_*^d^* (eV)** | **R factor** |
| --- | --- | --- | --- | --- | --- | --- |
| **Ni-foil** | Ni-Ni | 12.0 | 2.48 ± 0.01 | 0.0061 | 7.4 ± 0.4 | 0.0016 |
| **Fe-foil** | Fe-Fe | 8.0 | 2.48 ± 0.01 | 0.0053 | 6.3 ± 0.6 | 0.0019 |
| **Mo-foil** | Mo-Mo | 8.0 | 2.73 ± 0.01 | 0.0043 | 5.7 ± 0.7 | 0.0025 |
| **MoO_2_** | Mo-O | 6.0 | 2.00 ± 0.01 | 0.0067 | 5.2 ± 0.3 | 0.0033 |
| **Ni_3_Fe/MoO_2_/CW** | Ni-Fe (1st) | 3.5 | 2.39 ± 0.01 | 0.0072 | 7.1 ± 1.2 | 0.0021 |
|  | Fe-Ni (2nd) | 8.7 | 2.41 ± 0.01 | 0.0059 | 6.2 ± 0.8 | 0.0038 |
|  | Mo-O | 6.3 | 2.12 ± 0.01 | 0.0047 | 5.6 ± 2.3 | 0.0027 |
| **Ni_3_Fe/CW** | Ni-Fe (1st) | 3.1 | 2.46 ± 0.01 | 0.0081 | 7.3 ± 1.8 | 0.0044 |
|  | Fe-Ni (2nd) | 9.2 | 2.47 ± 0.01 | 0.0075 | 6.9 ± 1.4 | 0.0071 |

*^a^CN*: coordination numbers; *^b^R*: bond distance; *^c^σ*^2^: Debye-Waller factors; *^d^*Δ*E*_0_: the inner potential correction. R factor: goodness of fit. Error bounds that characterize the structural parameters obtained by EXAFS spectroscopy were estimated as CN±20%; R ± 1%; σ2 ± 20%.

**Table S3.** Average Bader charges of the elements in various electrocatalyst models.

| **Bader Charge** | **Ni_3_Fe/MoO_2_/CW** | **Ni_3_Fe/CW** | **MoO_2_/CW** |
| --- | --- | --- | --- |
| **Mo** | −1.8697 | N/A | −1.8327 |
| **O** |   0.9527 | N/A |   0.9304 |
| **Ni** |    0.0189 | 0.0881 | −0.1795 |
| **Fe** | − 0.4260 | 0.2643 | N/A |

*The positive value indicates the gain of electrons and negative value indicates the loss of electrons.

**Table S4.** Summary of recently reported HER overpotentials corresponding to catalysts in 1.0 M KOH solution.

| **Catalysts** | **Overpotential @*j* (mV)** | **Ref** |
| --- | --- | --- |
| Ni_3_Fe/MoO_2_/CW | 45@−10  128@−100  279@−500 | This work |
| NiMo/Int/NF | 80.2@−1000 | [4] |
| RuMo/Cu_2_O@C | 178@−500 | [5] |
| Co_3_O_4_/NWM | 185@−500 | [6] |
| N-P-B/Ni Foam | 276@−500 | [7] |
| NPCu/Cu_5_Zr | 280@−500 | [8] |
| Nano-KFO/NF | 281@−500 | [9] |
| CuMo_6_S_8_ | 290@−500 | [10] |
| Mo-NiCu_array_/CM | 300@−500 | [11] |
| Cu NWs@NiFe-Pt | 309@−500 | [12] |
| NiFeP@Ni_2_P/MoO_x_ | 331@−500 | [13] |
| NF@NiFe-LDH-1.5-4 | 356@−500 | [14] |
| Mn-doped Ni_2_P/Fe_2_P | 365@−500 | [15] |
| LiCoBPO/NF | 370@−500 | [16] |
| NiMo-Pi | 461@−500 | [17] |
| Mo-Ni_3_S_4_/CW | 270@−100 | [18] |
| V-Co_2_P@HE | 33@−10 | [19] |
| NiFeCo/CW-300 | 35@−10 | [20] |
| Ni(OH)_2_/NiO-C/WO_3_ | 53@−10 | [21] |
| W-NT@NF-3 | 88@−10 | [22] |
| Ni-Mo_2_C/N-CW | 89@−10 | [23] |
| A-NiMoO-P | 65@−100 | [24] |
| Ti64-H/Ni/CN | 92@−10 | [25] |
| O-NiMoP_f_/NF | 97@−10 | [26] |
| Fe_0.074_NiP/NWM | 108@−10 | [27] |
| NSP CCN-rGO | 113@−10 | [28] |
| NiFe-MOF | 116@−10 | [29] |
| Ni_3_Fe@CNTs/CeOx/NF | 125@−10 | [30] |
| La-MoP@NC | 129.3@−10 | [31] |
| SiF_6_@MOF-1 | 148@−10 | [32] |
| NiSe_2_ | 149.7@−10 | [33] |
| CoFe/C-650 | 164@−10 | [34] |
| Co-WS_2_ | 183@−100 | [35] |
| Cu_50_W_50_ | 65@−10 | [36] |
| NV-31-CP | 55@−10 | [37] |
| g-C_3_N_4_/rGO | 183@−10 | [38] |
| PS-Cu | 121@−10 | [39] |
| B_4.7_-Ni_4_Mo/NF | 47.44@−100 | [40] |
| Ni_2_P-Ni_12_P_5_@Ni_3_S_2_ | 32@−10 | [41] |
| CN/CNL/MoS_2_ | 106@−10 | [42] |
| Mo-MoS_2_ MSH | 138@−10 | [43] |
| W-NT@NF | 88@−10 | [23] |
| i-MoN | 136@−1000 | [44] |
| E-Co SAs | ~280@−100 | [45] |
| CoP/CoMoP | 94@−100 | [46] |
| W_1_Mo_1_-NG | ~150@−100 | [47] |
| Cu-FeOOH/Fe_3_O_4_ | 129@−100 | [48] |
| Mn-Co_1.29_Ni_1.71_O_4_ | 203.5@−100 | [49] |
| NiFe_2_O_4_/ZnO/g-C_3_N_4_ | 224@−100 | [50] |
| FeOOH/NIF | 232.5@−100 | [51] |
| FeS_2-x_Se_x_/CoS@CC | 285.2@−100 | [52] |

**Table S5.** Summary of recently reported OER overpotentials corresponding to catalysts in 1.0 M KOH solution.

| **Catalysts** | **Overpotential @*j* (mV)** | **Ref** |
| --- | --- | --- |
| Ni_3_Fe/MoO_2_/CW | 278@500 | This work |
| CAPist-L1 | 210@500 | [53] |
| RuMoNi | 390@500 | [54] |
| (Ni,Co)O-Co_3_O_4_ | 356@10 | [55] |
| LFA | 238@500 | [56] |
| (NiCoMnCrFe)_3_O_4_ | 268@500 | [57] |
| NiFe-S-0.6 | 294@500 | [58] |
| Ag/NiFe LDH | 267@500 | [59] |
| Fe_.92_Co_.08_S@SC/SCWF | 281@500 | [60] |
| CCF-1 | 290@500 | [61] |
| Fe-CoP/NF | 295@500 | [62] |
| NiFe-MOF | 297@500 | [63] |
| FeF@Co-Fe-5 | 298@500 | [64] |
| R-NiFeO_x_H_y_ | 302@500 | [65] |
| d-(Fe,Ni)OOH | 300@500 | [66] |
| SS-AC-SR | 229@500 | [67] |
| NiFe-O NAs/Fe foam | 323.7@500 | [68] |
| Co_9_S_8_@Fe_3_O_4_ | 350@500 | [69] |
| Por-NiP-Cr-BDD | 362@500 | [70] |
| NiFe(OH)_x_/IF | 390@500 | [71] |
| SSM-NC_s_/Ni(Fe)O_x_H_y_ | 280@500 | [72] |
| P-NiCoV-LTH/NF | 340@500 | [73] |
| N/Fe-Ni_2_P/Ni_12_P_5_ | 281@500 | [74] |
| FeNiMn_0.4_-MIL-53/NF | 290@500 | [75] |
| FeP/Ni_2_P | 280@500 | [76] |
| Se-doped FeOOH | 348@500 | [77] |
| Ni_83_Fe_17_-ONCAs | 255@500 | [78] |
| Co_9_S_8_@Fe_3_O_4_ | 350@500 | [69] |
| Ni_0.8_Fe_0.2_–AHNA | 248@500 | [79] |
| CoNi-LDH/Fe MOF/NF | 277@500 | [80] |
| Co_2_C-NiTe/SS | ~470@300  ~520@500 | [81] |
| FeNi-WO_2_/NF | 470@500 | [82] |
| S-HEO | 490@500 | [83] |
| FNF-0.5 | ~330@500  395@1000 | [84] |
| FeNbN | ~360@500  438@1000 | [85] |
| NiFe-LDH/FeOOH | ~300@500  359@1000 | [86] |
| Fe_2_O_3_/NiFe-LDHs | 220@500 | [87] |
| Fe-Ni_3_S_2_ | 332@500 | [88] |
| 0.01 S-FeOOH_+1000_/IF | 324@500  358@1000 | [89] |
| S-NiFeZn LDH/NF | 580@500 | [90] |

**Table S6.** Elemental composition of Ni_3_Fe/MoO_2_/CW after stability test obtained from ICP-MS.

| **Elements**  **Catalysts** | **Ni** **(wt.%)** | **Fe (wt.%)** | **Mo** **(wt.%)** |
| --- | --- | --- | --- |
| **After HER** | 20.23 | 6.16 | 73.61 |
| **After OER** | 20.19 | 6.12 | 73.69 |
| **After Overall Water Electrolysis (Anode)** | 20.42 | 6.17 | 73.41 |
| **After Overall Water Electrolysis (Cathode)** | 20.25 | 5.99 | 73.76 |

**Table S7.** XPS analysis of elemental changes in Ni_3_Fe/MoO_2_/CW before and after stability test.

|  | **Ni^0^** | **Ni^2+^** | **Fe^0^** | **Fe^2+^** | **Fe^3+^** | **Mo^4+^** | **Mo^6+^** |
| --- | --- | --- | --- | --- | --- | --- | --- |
| **Before stability test** | 19.08% | 80.92% | 30.80% | 24.25% | 44.95% | 22.39% | 77.61% |
| **After HER stability test** | 23.02% | 76.98% | 35.31% | 22.67% | 42.02% | 25.06% | 74.94% |
| **After OER stability test** | 18.57% | 81.43% | 27.88% | 25.38% | 46.74% | 18.21% | 81.79% |

**Table S8.** The potential required for the recent reported catalyst in water electrolysis.

| **Catalysts** | **Voltage @*j* (V)** | **Ref** |
| --- | --- | --- |
| Ni_3_Fe/MoO_2_/CW | 1.55@100  1.71@200 | This work |
| Ni_2_P/NiSe_2_@MXene/NF | 1.50@10 | [91] |
| CoP/FeCoP_x_ | 1.53@10 | [92] |
| ZCF-LDH/NF | 1.61@10 | [93] |
| Co-Fe_3_O_4_/IF | 1.62@10 | [94] |
| Co/CoMoN/NF | 1.56@100 | [95] |
| Fe_2_P/Co_2_N | 1.561@100 | [96] |
| Co_8_FeV@CC | 1.65@100 | [97] |
| S, P-(Ni, Mo, Fe)OOH/NiMoP/Wood | 1.69@100 | [98] |
| NC@Fe_0.1_-CoP/Co_2_P/NF | 1.70@100 | [99] |
| Mo-NiP_x_/NiS_y_ | 1.70@100  ~1.96@200 | [100] |
| P-CoMo_2_S_4_/Co_4_S_3_-Co_2_P | 1.74@100 | [101] |
| Co@NCNT/CW | 1.78@100 | [102] |
| NFM-2 | 1.82@100 | [103] |
| NiFe RTNC/CW | 1.92@100 | [104] |
| HP Ni-P | 1.68@100 | [105] |
| Cu_2_O-CoO/CF | 1.78@100 | [106] |
| 0.005-NiCoP@NF | 1.86@100 | [107] |
| Mn-NiCoP | 1.69@100 | [108] |
| Co_4_N-CeO_2_/GP | 1.728@100 | [109] |
| Ni-MoN | 1.613@100 | [44] |
| CoNi-LDH/Fe MOF/NF | 1.61@100 | [80] |
| iMoFeO | 1.69@100 | [110] |
| NiCoP@NF-100 | 1.80@100  1.87@200 | [111] |
| Fe-Ni_2_P@C/NF | 1.66@100  ~1.71@200 | [112] |
| a-CoMoP_x_/CF | 1.70@100  ~1.73@200 | [113] |
| Ni_x_S_y_@MnO_x_H_y_/NF | 1.829@100  1.888@200 | [114] |
| Ni@C-MoO_2_/NF | 1.67@100 | [115] |
| np-NiMnFeMo | ~1.77@100  ~1.93@200 | [116] |
| CoS_x_-Ni_3_S_2_/NF | 1.63@100 | [117] |
| Ni-Fe-S | 1.86@100 | [118] |
| NiP_2_/NiSe_2_ | 1.80@100 | [119] |
| NiS_0.5_Se_0.5_ | ~1.76@100  ~1.88@200 | [120] |
| NiFe-LDH@NiMo-H_2_@NF | 1.52@100  ~1.60@200 | [121] |
| DMC-4 | 2.14@100 | [122] |
| Fe-P-CMO | 1.59@100 | [123] |
| CoPʘNPC/CTs | 1.66@100  1.78@200 | [124] |

**Table S9.** The BET result of Ni_3_Fe/MoO_2_/CW and Precursor.

| **Sample** | **Average Pore Size**  **(nm)** | **S_BET_**  **(m^2^/g)** | **V_micro_**  **(cm^3^/g)** |
| --- | --- | --- | --- |
| **Ni_3_Fe/MoO_2_/CW** | 12.85 | 167.50 | 0.053 |
| **Ni_3_Fe/CW** | 7.69 | 109.08 | 0.022 |
| **MoO_2_/CW** | 4.63 | 83.27 | 0.016 |
| **Ni_3_Fe/MoO_2_/CW (350 °C)** | 10.71 | 161.23 | 0.041 |
| **Ni_3_Fe/MoO_2_/CW (550 °C)** | 10.32 | 106.66 | 0.042 |

1. **References**

[1] L. Li, L. Ge, G. Qian, Y. Wang, J. Li, X. Cao, Y. Xu, R. Zhang, J. Chen, P. Tsiakaras, *Appl. Catal. B: Environ.* **2025**, *377*, 125465.

[2] C. Triolo, K. Moulaee, A. Ponti, G. Pagot, V. Di Noto, N. Pinna, G. Neri, S. Santangelo, *Adv Funct Materials* **2024**, *34*, 2306375.

[3] H. Zhang, Q. An, Y. Su, X. Quan, S. Chen, *J. Hazard. Mater.* **2023**, *448*, 130987.

[4] G. Lin, A. Dong, Z. Li, W. Li, X. Cao, Y. Zhao, L. Wang, L. Sun, *Adv. Mater.* **2025**, *37*, 2507525.

[5] X. Yang, H. Shen, X. Xiao, Z. Li, H. Liang, S. Chen, Y. Sun, B. Jiang, G. Wen, S. Wang, L. Zhang, *Adv. Mater.* **2025**, *37*, 2416658.

[6] C. Li, X. Luo, Y. Wang, M. Zhu, D. Li, S. Guo, W. Wang, X. Xu, *Adv. Sci.* **2025**, e08013.

[7] W. Hao, R. Wu, H. Huang, X. Ou, L. Wang, D. Sun, X. Ma, Y. Guo, *Energy Environ. Sci.* **2020**, *13*, 102.

[8] H. Shi, Y. Zhou, R. Yao, W. Wan, Q. Zhang, L. Gu, Z. Wen, X. Lang, Q. Jiang, *Research* **2020**, *2020*, 2020/2987234.

[9] J. Jian, W. Chen, D. Zeng, L. Chang, R. Zhang, M. Jiang, G. Yu, X. Huang, H. Yuan, S. Feng, *J. Mater. Chem. A* **2021**, *9*, 7586.

[10] H. Liu, R. Xie, Y. Luo, Z. Cui, Q. Yu, Z. Gao, Z. Zhang, F. Yang, X. Kang, S. Ge, S. Li, X. Gao, G. Chai, L. Liu, B. Liu, *Nat. Commun.* **2022**, *13*, 6382.

[11] K. Wei, H. Hu, Y. Song, Y. Wang, Y. Meng, Y. Wang, J. Zhou, F. Gao, *Sep. Purif. Technol.* **2023**, *326*, 124814.

[12] Z. Ni, C. Luo, B. Cheng, P. Kuang, Y. Li, J. Yu, *Appl. Catal. B: Environ.* **2023**, *321*, 122072.

[13] Y. Luo, S. Wu, P. Wang, H. Ranganathan, Z. Shi, *J. Colloid Interface Sci.* **2023**, *648*, 551.

[14] X. Li, C. Liu, Z. Fang, L. Xu, C. Lu, W. Hou, *Small* **2022**, *18*, 2104354.

[15] Y. Luo, P. Wang, G. Zhang, S. Wu, Z. Chen, H. Ranganathan, S. Sun, Z. Shi, *Chem. Eng. J.* **2023**, *454*, 140061.

[16] P. W. Menezes, A. Indra, I. Zaharieva, C. Walter, S. Loos, S. Hoffmann, R. Schlögl, H. Dau, M. Driess, *Energy Environ. Sci.* **2019**, *12*, 988.

[17] G. Yuan, R. Zhang, Y. Wang, H. Xin, J. Wang, M. Chen, *Mol. Catal.* **2023**, *550*, 113541.

[18] Z. Shi, C. Mao, L. Zhong, J. Peng, M. Liu, H. Li, J. Huang, *Appl. Catal. B: Environ.* **2023**, *339*, 123123.

[19] W. Ma, Z. Qiu, J. Li, L. Hu, Q. Li, X. Lv, J. Dang, *J. Energy Chem.* **2023**, *85*, 301.

[20] Y. Qian, M. Hu, L. Li, S. Cao, J. Xu, J. Hong, X. Liu, J. Xu, C. Guo, *Fuel* **2024**, *361*, 130653.

[21] J. Zhao, Y. Zhang, H. Guo, J. Ren, H. Zhang, Y. Wu, R. Song, *Chem. Eng. J.* **2022**, *433*, 134497.

[22] Z. Liu, S. Luo, X. Yin, S. Zhang, M. Guo, *Sep. Purif. Technol.* **2024**, *341*, 126929.

[23] M. Liu, W. Zou, S. Qiu, N. Su, J. Cong, L. Hou, *Adv. Funct. Mater.* **2024**, *34*, 2310155.

[24] Q. Li, C. Chen, W. Luo, X. Yu, Z. Chang, F. Kong, L. Zhu, Y. Huang, H. Tian, X. Cui, J. Shi, *Adv. Energy Mater.* **2024**, *14*, 2304099.

[25] B. Guo, J. Lin, F. Mo, Y. Ding, T. Zeng, H. Liang, L. Wang, X. Chen, J. Mo, D. Li, H. Y. Yang, J. Bai, *Small* **2024**, *20*, 2312216.

[26] H. Jiang, M. Sun, S. Wu, B. Huang, C. Lee, W. Zhang, *Adv. Funct. Mater.* **2021**, *31*, 2104951.

[27] B. Hui, J. Li, Y. Lu, K. Zhang, H. Chen, D. Yang, L. Cai, Z. Huang, *J. Energy Chem.* **2021**, *56*, 23.

[28] R. Su, R. Wang, H. Wang, Y. Liu, Y. Yan, H. Yin, Y. Kong, F. Shaik, B. Jiang, *Int. J. Hydrog. Energy* **2025**, *117*, 24.

[29] B. Dai, X. Wei, L. Chen, X. Bao, Q. Zhong, H. Qu, *J. Colloid Interface Sci.* **2025**, *682*, 80.

[30] M. Sun, S. Zhang, Y. Li, C. Yang, Y. Guo, L. Yang, S. Xu, *Dalton Trans.* **2023**, *52*, 9254.

[31] P. Wei, X. Li, Z. He, Z. Li, X. Zhang, X. Sun, Q. Li, H. Yang, J. Han, Y. Huang, *Appl. Catal. B: Environ.* **2021**, *299*, 120657.

[32] U. Phadikar, R. Sahoo, M. C. Das, T. Kuila, *Int. J. Hydrog. Energy* **2025**, *117*, 135.

[33] S. Zhang, R.-Y. Li, X. Li, Y. Tian, R. Zhao, J. Xiang, F. Wu, D. Zhao, *Mater. Res. Bull.* **2025**, *189*, 113463.

[34] K. Srinivas, Y. Chen, Z. Su, B. Yu, M. Karpuraranjith, F. Ma, X. Wang, W. Zhang, D. Yang, *Electrochim. Acta* **2022**, *404*, 139745.

[35] R.-X. Wang, L. Yang, H.-Y. Chen, N. Wang, W.-J. Zhang, R. Li, Y.-Q. Chen, C. You, S. Ramakrishna, Y.-Z. Long, *J. Colloid Interface Sci.* **2024**, *667*, 192.

[36] X. Jian, W. Zhang, Y. Yang, Z. Li, H. Pan, Q. Gao, H.-J. Lin, *ACS Catal.* **2024**, *14*, 2816.

[37] M. Maji, N. Dihingia, S. Dutta, S. Parvin, S. K. Pati, S. Bhattacharyya, *J. Mater. Chem. A* **2022**, *10*, 24927.

[38] H. Choi, S. Surendran, Y. Sim, M. Je, G. Janani, H. Choi, J. K. Kim, U. Sim, *Chem. Eng. J.* **2022**, *450*, 137789.

[39] W. Kang, Y. Feng, Z. Li, W. Yang, C. Cheng, Z. Shi, P. Yin, G. Shen, J. Yang, C. Dong, H. Liu, F. Ye, X. Du, *Adv. Funct. Mater.* **2022**, *32*, 2112367.

[40] P. Liu, Y. Shi, X. Zhang, J. Yin, D. Zhang, T. Wang, J. Fei, T. Zhan, G. Li, J. Lai, L. Wang, *Appl. Catal. B: Environ.* **2024**, *341*, 123332.

[41] H. Yang, P. Guo, R. Wang, Z. Chen, H. Xu, H. Pan, D. Sun, F. Fang, R. Wu, *Adv. Mater.* **2022**, *34*, 2107548.

[42] J. Dong, X. Zhang, J. Huang, J. Hu, Z. Chen, Y. Lai, *Chem. Eng. J.* **2021**, *412*, 128556.

[43] Z. Sun, L. Lin, M. Yuan, H. Yao, Y. Deng, B. Huang, H. Li, G. Sun, J. Zhu, *Nano Energy* **2022**, *101*, 107563.

[44] L. Wu, F. Zhang, S. Song, M. Ning, Q. Zhu, J. Zhou, G. Gao, Z. Chen, Q. Zhou, X. Xing, T. Tong, Y. Yao, J. Bao, L. Yu, S. Chen, Z. Ren, *Adv. Mater.* **2022**, *34*, 2201774.

[45] X. Liu, L. Zheng, C. Han, H. Zong, G. Yang, S. Lin, A. Kumar, A. R. Jadhav, N. Q. Tran, Y. Hwang, J. Lee, S. Vasimalla, Z. Chen, S. Kim, H. Lee, *Adv. Funct. Mater.* **2021**, *31*, 2100547.

[46] X. Huang, X. Xu, X. Luan, D. Cheng, *Nano Energy* **2020**, *68*, 104332.

[47] Y. Yang, Y. Qian, H. Li, Z. Zhang, Y. Mu, D. Do, B. Zhou, J. Dong, W. Yan, Y. Qin, L. Fang, R. Feng, J. Zhou, P. Zhang, J. Dong, G. Yu, Y. Liu, X. Zhang, X. Fan, *Sci. Adv.* **2020**, *6*, eaba6586.

[48] C. Yang, W. Zhong, K. Shen, Q. Zhang, R. Zhao, H. Xiang, J. Wu, X. Li, N. Yang, *Adv. Energy Mater.* **2022**, *12*, 2200077.

[49] Y. Cheng, X. Guo, Z. Ma, K. Dong, L. Miao, S. Du, *Molecules* **2025**, *30*, 1162.

[50] V. Siva, L. Kumaresan, P. Velusamy, G. Palanisamy, T. Chellapandi, N. Dineshbabu, *Mater. Sci. Semicond. Process.* **2025**, *195*, 109589.

[51] X. Du, J. Zhang, M. Zhang, H. Wei, X. Lin, W. Guo, P. Zhang, Z. Luo, *Green Chem.* **2025**, *27*, 7380.

[52] X. Yin, X. Mu, S. Qi, H. Li, M. Qi, A. Yusuf, *Res. Chem. Intermed.* **2025**, *51*, 3229.

[53] Z. Li, G. Lin, L. Wang, H. Lee, J. Du, T. Tang, G. Ding, R. Ren, W. Li, X. Cao, S. Ding, W. Ye, W. Yang, L. Sun, *Nat. Catal.* **2024**, *7*, 944.

[54] X. Kang, F. Yang, Z. Zhang, H. Liu, S. Ge, S. Hu, S. Li, Y. Luo, Q. Yu, Z. Liu, Q. Wang, W. Ren, C. Sun, H.-M. Cheng, B. Liu, *Nat. Commun.* **2023**, *14*, 3607.

[55] V. D. M. Andrade, S. M. Lopes, R. A. Raimundo, R. F. Alves, A. J. M. Araújo, P. S. Vieira, F. F. Da Silva, M. A. Morales, D. A. Macedo, D. P. Fagg, G. D. S. Souza, *Mater. Sci. Semicond. Process.* **2025**, *195*, 109643.

[56] J. Wang, C. Liang, X. Ma, P. Liu, W. Pan, H. Zhu, Z. Guo, Y. Sui, H. Liu, L. Liu, C. Yang, *Adv. Mater.* **2024**, *36*, 2307925.

[57] J. Fan, X. Xiang, Y. Liu, X. Yang, N. Shi, D. Xu, C. Zhou, M. Han, J. Bao, W. Huang, *SusMat* **2025**, e70010.

[58] L. He, N. Wang, M. Xiang, L. Zhong, S. Komarneni, W. Hu, *Appl. Catal. B: Environ.* **2024**, *345*, 123686.

[59] S. Liu, S. Ren, R.-T. Gao, X. Liu, L. Wang, *Nano Energy* **2022**, *98*, 107212.

[60] B. Zhao, W. Zeng, W. Zhang, S. Chen, H. Xu, Y. Liao, Y. Liao, Y. Qing, Y. Wu, *Appl. Catal. B: Environ.* **2024**, *350*, 123947.

[61] N. K. Dang, J. N. Tiwari, S. Sultan, A. Meena, K. S. Kim, *Chem. Eng. J.* **2021**, *404*, 126513.

[62] L. Cao, Y. Hu, S. Tang, A. Iljin, J. Wang, Z. Zhang, T. Lu, *Adv. Sci.* **2018**, *5*, 1800949.

[63] J. Zhou, Z. Han, X. Wang, H. Gai, Z. Chen, T. Guo, X. Hou, L. Xu, X. Hu, M. Huang, S. V. Levchenko, H. Jiang, *Adv. Funct. Mater.* **2021**, *31*, 2102066.

[64] B. Shao, W. Pang, X.-Q. Tan, C. Tang, Y. Deng, D. Huang, J. Huang, *J. Electroanal. Chem.* **2020**, *856*, 113621.

[65] J. Liu, W. Du, S. Guo, J. Pan, J. Hu, X. Xu, *Adv. Sci.* **2023**, *10*, 2300717.

[66] L. Wu, M. Ning, X. Xing, Y. Wang, F. Zhang, G. Gao, S. Song, D. Wang, C. Yuan, L. Yu, J. Bao, S. Chen, Z. Ren, *Adv. Mater.* **2023**, *35*, 2306097.

[67] C. Hou, L. Xue, J. Li, W. Ma, J. Wang, Y. Dai, C. Chen, J. Dang, *Acta Mater.* **2024**, *277*, 120176.

[68] X. Zhang, H. Zhu, Z. Zuo, M. Jin, O. Peng, Q. Lian, Y. Huang, P. Cheng, Z. Ai, S. Xiang, A. Amini, S. Song, F. Jia, Z. Guo, C. Cheng, *Chem. Eng. J.* **2024**, *490*, 151705.

[69] Q. Ji, Y. Kong, H. Tan, H. Duan, N. Li, B. Tang, Y. Wang, S. Feng, L. Lv, C. Wang, F. Hu, W. Zhang, L. Cai, W. Yan, *ACS Catal.* **2022**, *12*, 4318.

[70] R. Zhu, J. Zang, L. Dong, X. Tian, F. Sun, Q. Fu, Z. Zhuang, Y. Wang, *Carbon* **2025**, *241*, 120392.

[71] S. Niu, W. Jiang, T. Tang, L. Yuan, H. Luo, J. Hu, *Adv. Funct. Mater.* **2019**, *29*, 1902180.

[72] J. Shen, M. Wang, L. Zhao, P. Zhang, J. Jiang, J. Liu, *J. Power Sources* **2018**, *389*, 160.

[73] Q. Liu, J. Huang, X. Zhang, L. Cao, D. Yang, J. Kim, L. Feng, *ACS Sustainable Chem. Eng.* **2020**, *8*, 16091.

[74] X. Jiang, X. Yue, Y. Li, X. Wei, Q. Zheng, F. Xie, D. Lin, G. Qu, *Chem. Eng. J.* **2021**, *426*, 130718.

[75] F. Zheng, W. Zhang, X. Zhang, Y. Zhang, W. Chen, *Adv. Funct. Mater.* **2021**, *31*, 2103318.

[76] F. Yu, H. Zhou, Y. Huang, J. Sun, F. Qin, J. Bao, W. A. Goddard, S. Chen, Z. Ren, *Nat. Commun.* **2018**, *9*, 2551.

[77] S. Niu, W.-J. Jiang, Z. Wei, T. Tang, J. Ma, J.-S. Hu, L.-J. Wan, *J. Am. Chem. Soc.* **2019**, *141*, 7005.

[78] P. Liu, B. Chen, C. Liang, W. Yao, Y. Cui, S. Hu, P. Zou, H. Zhang, H. J. Fan, C. Yang, *Adv. Mater.* **2021**, *33*, 2007377.

[79] C. Liang, P. Zou, A. Nairan, Y. Zhang, J. Liu, K. Liu, S. Hu, F. Kang, H. J. Fan, C. Yang, *Energy Environ. Sci.* **2020**, *13*, 86.

[80] Q.-N. Bian, B.-S. Guo, D.-X. Tan, D. Zhang, W.-Q. Kong, C.-B. Wang, Y.-Y. Feng, *ACS Appl. Mater. Interfaces* **2024**, *16*, 14742.

[81] T. Munawar, A. Bashir, A. El-Marghany, L. Shen, Z. Tu, S. Fatima, A. Ghafoor Abid, S. Alim Khan, M. Koc, C.-F. Yan, F. Iqbal, *Fuel* **2024**, *375*, 132445.

[82] K. Zhang, Y. Luo, H. Wang, J. Li, Y. Wang, X. Du, G. Liu, *Chem. Eng. J.* **2025**, *511*, 161686.

[83] S. C. Karthikeyan, S. Ramakrishnan, S. Prabhakaran, M. R. Subramaniam, M. Mamlouk, D. H. Kim, D. J. Yoo, *Small* **2024**, *20*, 2402241.

[84] P. Tan, Y. Wu, Y. Tan, Y. Xiang, L. Zhou, N. Han, Y. Jiang, S. Bao, X. Zhang, *Small* **2024**, *20*, 2308371.

[85] S. Seenivasan, J. Seo, *Chem. Eng. J.* **2023**, *454*, 140558.

[86] Y. Wang, L. Li, J. Shi, M. Xie, J. Nie, G. Huang, B. Li, W. Hu, A. Pan, W. Huang, *Adv. Sci.* **2023**, *10*, 2303321.

[87] C.-F. Li, L.-J. Xie, J.-W. Zhao, L.-F. Gu, J.-Q. Wu, G.-R. Li, *Appl. Catal. B: Environ.* **2022**, *306*, 121097.

[88] D. Li, W. Wan, Z. Wang, H. Wu, S. Wu, T. Jiang, G. Cai, C. Jiang, F. Ren, *Adv. Energy Mater.* **2022**, *12*, 2201913.

[89] X.-Y. Zhang, F.-T. Li, Y.-W. Dong, B. Dong, F.-N. Dai, C.-G. Liu, Y.-M. Chai, *Appl. Catal. B: Environ.* **2022**, *315*, 121571.

[90] F. Shi, L. Xiao, Z. Zhou, X. Zhao, Y. Liu, J. Mao, J. Qin, Y. Deng, J. Yang, *Adv. Funct. Mater.* **2025**, 2501070.

[91] I. Pathak, D. Acharya, K. Chhetri, Y. R. Rosyara, A. Muthurasu, T. Kim, T. H. Ko, H. Y. Kim, *Compos. B: Eng.* **2025**, *296*, 112238.

[92] J. Zhang, Y. Zhang, J. Zhou, H. Guo, L. Qi, *Small Methods* **2025**, *9*, 2401139.

[93] T. Govindaraj, C. Kanagaraj, E. S. Kumar, M. Navaneethan, *Int. J. Energy Res.* **2025**, *121*, 361.

[94] R. Liao, Z. Peng, X. Yang, J. Liu, J. Zhou, L. Yu, J. Liao, *Int. J. Hydrog. Energy* **2025**, *116*, 32.

[95] H. Ma, Z. Chen, Z. Wang, C. V. Singh, Q. Jiang, *Adv. Sci.* **2022**, *9*, 2105313.

[96] X. Zhou, Y. Mo, F. Yu, L. Liao, X. Yong, F. Zhang, D. Li, Q. Zhou, T. Sheng, H. Zhou, *Adv. Funct. Mater.* **2023**, *33*, 2209465.

[97] J. Lv, P. Liu, R. Li, L. Wang, K. Zhang, P. Zhou, X. Huang, G. Wang, *Appl. Catal. B: Environ.* **2021**, *298*, 120587.

[98] H. Chen, Y. Zou, J. Li, K. Zhang, Y. Xia, B. Hui, D. Yang, *Appl. Catal. B: Environ.* **2021**, *293*, 120215.

[99] J. Huo, Y. Ming, X. Huang, R. Ge, S. Li, R. Zheng, J. Cairney, S. X. Dou, B. Fei, W. Li, *J. Colloid Interface Sci.* **2025**, *678*, 669.

[100] J. Wang, M. Zhang, G. Yang, W. Song, W. Zhong, X. Wang, M. Wang, T. Sun, Y. Tang, *Adv. Funct. Mater.* **2021**, *31*, 2101532.

[101] K. Dong, D. T. Tran, X. Li, S. Prabhakaran, D. H. Kim, N. H. Kim, J. H. Lee, *Appl. Catal. B: Environ.* **2024**, *344*, 123649.

[102] J. Huang, Z. Shi, C. Mao, G. Yang, Y. Chen, *Small* **2024**, 2402511.

[103] N. S. Gultom, T.-S. Chen, M. Z. Silitonga, D.-H. Kuo, *Appl. Catal. B: Environ.* **2023**, *322*, 122103.

[104] X. Sheng, Y. Li, T. Yang, B. J. J. Timmer, T. Willhammar, O. Cheung, L. Li, C. J. Brett, S. V. Roth, B. Zhang, L. Fan, Y. Guo, X. Zou, L. Berglund, L. Sun, *Appl. Catal. B: Environ.* **2020**, *264*, 118536.

[105] D. Song, D. Hong, Y. Kwon, H. Kim, J. Shin, H. M. Lee, E. Cho, *J. Mater. Chem. A* **2020**, *8*, 12069.

[106] L. Xie, Q. Liu, X. He, Y. Luo, D. Zheng, S. Sun, A. Farouk, M. S. Hamdy, J. Liu, Q. Kong, X. Sun, *Chem. Commun.* **2023**, *59*, 10303.

[107] H. Liu, J. Li, Y. Zhang, R. Ge, J. Yang, Y. Li, J. Zhang, M. Zhu, S. Li, B. Liu, L. Dai, W. Li, *Chem. Eng. J.* **2023**, *473*, 145397.

[108] G. Ma, J. Ye, M. Qin, T. Sun, W. Tan, Z. Fan, L. Huang, X. Xin, *Nano Energy* **2023**, *115*, 108679.

[109] H. Sun, C. Tian, G. Fan, J. Qi, Z. Liu, Z. Yan, F. Cheng, J. Chen, C. Li, M. Du, *Adv. Funct. Mater.* **2020**, *30*, 1910596.

[110] R. Zhang, Q. Liu, L. Zhou, L. Wang, L. Cheng, A. Xie, H. Xu, Z. Bai, Y. Tang, P. Wan, *Int. J. Hydrog. Energy* **2024**, *82*, 1341.

[111] L. Chen, Y. Song, Y. Liu, L. Xu, J. Qin, Y. Lei, Y. Tang, *J. Energy Chem.* **2020**, *50*, 395.

[112] D. Li, Z. Li, R. Zou, G. Shi, Y. Huang, W. Yang, W. Yang, C. Liu, X. Peng, *Appl. Catal. B: Environ.* **2022**, *307*, 121170.

[113] H. Huang, A. Cho, S. Kim, H. Jun, A. Lee, J. W. Han, J. Lee, *Adv. Funct. Mater.* **2020**, *30*, 2003889.

[114] P. Wang, Y. Luo, G. Zhang, Z. Chen, H. Ranganathan, S. Sun, Z. Shi, *Nano-Micro Lett.* **2022**, *14*, 120.

[115] G. Qian, G. Yu, J. Lu, L. Luo, T. Wang, C. Zhang, R. Ku, S. Yin, W. Chen, S. Mu, *J. Mater. Chem. A* **2020**, *8*, 14545.

[116] H. Liu, C. Xi, J. Xin, G. Zhang, S. Zhang, Z. Zhang, Q. Huang, J. Li, H. Liu, J. Kang, *Chem. Eng. J.* **2021**, *404*, 126530.

[117] L. Jiang, N. Yang, C. Yang, X. Zhu, Y. Jiang, X. Shen, C. Li, Q. Sun, *Appl. Catal. B: Environ.* **2020**, *269*, 118780.

[118] Y. Wu, Y. Li, M. Yuan, H. Hao, X. San, Z. Lv, L. Xu, B. Wei, *Chem. Eng. J.* **2022**, *427*, 131944.

[119] L. Yang, L. Huang, Y. Yao, L. Jiao, *Appl. Catal. B: Environ.* **2021**, *282*, 119584.

[120] Y. Wang, X. Li, M. Zhang, Y. Zhou, D. Rao, C. Zhong, J. Zhang, X. Han, W. Hu, Y. Zhang, K. Zaghib, Y. Wang, Y. Deng, *Adv. Mater.* **2020**, *32*, 2000231.

[121] Y. Zhang, B. Feng, M. Yan, Z. Shen, Y. Chen, J. Tian, F. Xu, G. Chen, X. Wang, L. Yang, Q. Wu, Z. Hu, *Nano Res.* **2024**, *17*, 3769.

[122] J. Das, S. Mandal, A. Borbora, S. Rani, M. Tenjimbayashi, U. Manna, *Adv. Funct. Mater.* **2024**, *34*, 2311648.

[123] B. Wang, X. Chen, Y. He, Q. Liu, X. Zhang, Z. Luo, J. V. Kennedy, J. Li, D. Qian, J. Liu, G. I. N. Waterhouse, *Appl. Catal. B: Environ.* **2024**, *346*, 123741.

[124] D. Kong, Q. Xu, N. Chu, H. Wang, Y. V. Lim, J. Cheng, S. Huang, T. Xu, X. Li, Y. Wang, Y. Luo, H. Y. Yang, *Small* **2024**, *20*, 2310012.
